# The epidemiology of HIV and prevention needs among men who have sex with men in Africa

**DOI:** 10.7448/IAS.16.4.18972

**Published:** 2013-12-02

**Authors:** R Cameron Wolf, Alison Surdo Cheng, Laurent Kapesa

## Introduction

Men who have sex with men (MSM) in sub-Saharan Africa experience a high burden of HIV infection [1–3]. Strong epidemiological evidence comes from studies in Kenya [4–7], where an estimated 18.9% of MSM are HIV-positive [1]. Kenya's National AIDS Control Council has prioritized HIV programming for MSM in their National HIV Strategic Plan [8], with the aim to support more inclusive health services for MSM [9]. Implementation of Kenya's AIDS policies requires the ability of healthcare workers (HCWs) to deliver appropriate and sensitive services to MSM patients. Effective HCWs must have accurate knowledge of the sexual health issues of MSM, non-prejudicial attitudes and behavioural skills to treat MSM patients [10]. However, HCWs in Kenya, as elsewhere in sub-Saharan Africa, rarely receive specialized training on how to provide care for MSM [11].

To address this gap in training service providers, Kenya's National AIDS and STI Control Programme (NASCOP) developed an education training programme to strengthen HCWs’ skills and capacity to provide non-judgemental counselling and HIV healthcare services for MSM. The training programme incorporated two learning modalities: a computer-facilitated training programme covering eight modules [MSM and HIV in sub-Saharan Africa; stigma; identity, coming out and disclosure; anal sex and common sexual practices; HIV and sexually transmitted infections; mental health, anxiety, depression and substance use; condom and lubricant use; risk-reduction counselling] in addition to facilitated group discussions among programme trainees about the programme content and relevant clinical experiences working with MSM. Both learning modalities offer complementary approaches to educational training. Computer-facilitated training modules can offer a standardized and disseminable approach to improve HCWs’ knowledge and health service delivery skills for MSM patients [12], especially in settings such as Kenya where access to formal medical education is constrained. Supplementing the computer-facilitated training with opportunities for peer discussion and support among HCWs can potentially enhance the transfer of standardized learning to the workplace [13].

We conducted a preliminary pre-post-evaluation of HCWs who participated in the programme [14]. Quantitative findings showed improvements in MSM-related knowledge and reductions in discriminatory attitudes towards MSM. Effects were most pronounced among HCWs who had low levels of knowledge and/or more extreme negative attitudes towards MSM at baseline, and among HCW in clinical roles within governmental settings.

This article reports data from qualitative focus groups with participating HCWs, conducted prior to and three months after completion of the programme. The objectives of this analysis are to explore: (i) how HCWs characterized their professional challenges in serving MSM patients prior to the programme, (ii) how HCWs described the impacts of programme participation on their personal attitudes and professional capacities and (iii) how the computer-facilitated educational training programme can be improved.

## Methods

### Participants and intervention procedures

The study was conducted between October 2011 and March 2012 in four districts in coastal Kenya: Kilifi, Kilindini, Malindi and Mombasa. To recruit trainee participants, NASCOP issued announcements to 49 health facilities providing antiretroviral treatment in the four targeted districts. Announcements described the study as a two-day residential programme involving computer-facilitated training and group discussions on HIV and MSM. Volunteer participants completed informed consent procedures, and those who enrolled received 2000 Kenya shilling (approximately US $24) for travel and lodging adjacent to the training facility in Kilifi.

Participants were 74 HCWs from the four target districts. Fifty were females and 24 males, including 22 clinicians, 43 nurses and counsellors, and nine were administrators/managers. The average age was 34. All participants identified as Kenyan, 84% as Christian and 15% as Muslim. Eighty-six percent had no previous training about MSM or anal sexual practices. Three participants (two females and one male) were transferred to health facilities outside the study area after the initial training and could not participate in the follow-up focus groups.

A total of four groups were convened to participate in the two-day residential training (one group per district), with 18–19 participants per group. During Day 1, participants received a general overview of the programme, and each participant then independently self-administered the first four modules of the standardized, computer-facilitated training. Modules were designed to take up to two hours to complete. At the end of each module, participants answered a series of multiple-choice questions (up to 16 questions); to advance to the next module, participants were required to achieve a minimum score of 71% correct. After every two modules, participants engaged in a group discussion to reflect on the information and identify barriers and facilitators to improve on HIV prevention and other services for MSM in Coastal Kenya. A member of the research team facilitated group discussions. During Day 2, participants completed the final four modules and group discussions. At the end of Day 2, participants were asked to discuss work strategies for improving the quality of clinical care and HIV/STD testing for MSM patients in their districts. Research team members included an MSM counsellor, a community liaison officer, a senior research counsellor and a social scientist; teams were supported by two MSM members from a local non-governmental organization. Research team members received a comprehensive three-day training on the intervention objectives and procedures, including didactics and role-play opportunities for discussion and problem solving.

### Focus group discussions

Eight focus group discussions (FGDs) (each comprising 9–10 participants; two focus groups per training) were conducted with participating HCWs prior to the training and were repeated three months following completion of the training. Focus groups were semi-structured and facilitated by a member of the research team, with a co-facilitator present to observe and take notes. Discussion topics included: identification of subcategories of MSM and their characteristics; sexual practices of MSM and risks for HIV and STI transmission; practices for sexual history taking and sexual health examination with MSM; risk-reduction counselling for MSM; personal values and attitudes towards MSM; strategies to improve communication between HCWs and MSM patients. Most discussions were conducted in English, although participants were also encouraged to speak in Kiswahili depending on their preference and language skills. All discussions were audiotaped, transcribed and entered into NVivo. FGDs conducted in Kiswahili were translated into English.

Analyses of qualitative data followed the “framework approach” described by Ritchie and Spencer [15], which involves systematic coding to identify and define concepts emerging from the data, mapping the concepts, creating typologies, finding associations between concepts and seeking explanations from the data. Data were coded by two independent research team members to ensure that interpretations of quotes were consistent and that data analysis was rigorous and transparent. The main concepts emerging from the data included: secondary stigma, professional training and service barriers to MSM patients; types of and justifications for social discrimination towards MSM in Kenyan culture; invisibility and silence about homosexuality in Kenyan culture; and subjective theories about the origins and nature of homosexuality. Differences among coders were resolved by group discussion involving other members of the research team.

The study procedures were approved by the ethical review board at the Kenya Medical Research Institute. All participants provided written informed consent for the FGD.

## Results

### Discussion of MSM-related attitudes, beliefs and behaviours before training

#### Secondary stigma

For most participants, secondary stigma was a dominant concern. Secondary stigma refers here to negative judgements from peers and community members for being associated with MSM. Participants cautioned that professional trainings focused on MSM would deter many health professionals from participation:

To me, the term MSM is stigmatizing because naturally, a man is not supposed to have sex with another man. As for this training … The [invitation] letter was written, ‘MSM training’. When we informed them, people were like, ‘An MSM training, what is MSM?’ … Some individuals declined to go for the training, ‘I can't go for such training’.

Several participants feared that colleagues would question their willingness to serve MSM patients, and perhaps suspect the HCWs of being MSM themselves:

You know MSM, as he had mentioned, are regarded as outcasts. Therefore, if you offer to treat them in your clinic, the community will perceive it as … the clinicians are also MSM.

Owing to this fear, many HCWs described minimizing the amount of time with MSM patients. For example, one participant described having a basic willingness to serve MSM patients, but would allocate the shortest time possible:

The fear of being associated, that's what is making us spend as little time with MSM clients when they come to our facilities. You will hurriedly clear him out.

However, fear of secondary stigma was not consistently expressed by all members of the discussion. A small subset of participants who had previous education and sensitization on MSM prior to the training reported comfort in attending to MSM patients. Consequently, these HCWs had become MSM patient advocates and educators in their clinics prior to engagement in this research study:

I was trained … on issues to do with MSM. Last week, I met an MSM client who was HIV positive. It was in one of our departments and the nurse was like, ‘… you are the person who deals with these kind of clients’. I told her to refer the client in my office … Actually, I had to take [my colleague] for an MSM training. Her attitude has really changed and she is a now a different person.

#### Inadequate professional training and resources

Participants acknowledged having little or no education about MSM health. Indeed, prior to the training programme, many HCWs expressed a sense of denial about the existence of MSM. For example, one reported that:

I tend to reason differently when it comes to MSM. I sometimes tell myself, no, this doesn't exist; this is not possible.

Across multiple discussions, others questioned whether MSM are present in their local communities:

Some of us are really green, we just hear stories on internet that some men are having sex with other men but we have never had an interaction with the MSM.

MSM are unheard of in the place I come from.

HCWs who acknowledged the presence of MSM patients in their clinics described feeling inadequately prepared to provide services. Those with prior experience consulting MSM patients described specific challenges in diagnosing and treating rectal STIs, and argued for more appropriate guidelines:

Of late, it's only a few individuals who have been trained in our facility. We don't have a guideline, yet we see them daily. We have no idea on how to manage infections affecting men who have sex with other men …

Most of the medical personnel are not sensitized on issues to do with anal STIs and they are also not indicated in the STI charts. They only specify about urethral discharge, cervicitis, urethritis in men, PID [pelvic inflammatory disease] etc. It doesn't mention the anus.

Lacking the knowledge, skills and treatment guidelines for rectal STIs, HCWs often relied on guesswork and assumptions. Participants recognized the likelihood of under-diagnosing or misdiagnosing rectal infections transmitted through anal sex.

And when we are counselling or probing them about sex, we only ask them, ‘Do you usually have sex?’ When they say yes, we don't probe further to know the type of sex i.e., we just assume it is heterosexual. The medics are also not trained and if an individual comes with an anal complaint, they assume that it is haemorrhoids and refer them for surgery.

HCWs described how limitations in assessment forms reinforce the invisibility of MSM in their clinics. By not collecting information about same-sex behaviour or anal sex practices, these topics are reinforced as taboo issues that warrant silence and discomfort.

Most of the tools and the working conditions are not accommodative for this line of sexual orientation. I have never seen a tool in the CCC [comprehensive care centre] or the TB clinic asking for the clients’ sexual orientation. So it's like, ‘I don't need to know of what you do’ … Therefore, the tools should be designed to capture the sexual orientation of a person so that the health workers can have a feel that it is a part of the health issues and not a gossip.

Additional resource limitations for treating MSM were discussed. HCWs reported on the inconsistent supply of lubricants for use during anal sex, and also described how the physical structure of the health facility hinders their ability to provide privacy and confidentiality for sexual health consultations.

The MSM usually come to the clinics and ask for the lubricants or condoms but you will find that the lubricants are not available; it's only the condoms.

I think there is no confidentiality because of the way our health facilities have been structured, i.e., someone can bump in while you are attending to a client. You could be talking of sensitive issues but other staffs won't bother. They will sit on the other side and do their stuff. So the client might not be free to open up.

#### Personal and social homophobia

Many HCWs acknowledged holding prejudiced views towards MSM. A number of participants commented on how negative judgements towards MSM may influence the provision of services.

We perceive them negatively and feel that they don't deserve our services.

Some health workers don't like to examine them. They claim that such infections are self-inflicted.

HCWs reflected on the influences of culture and religion on their treatment of MSM patients. When reminded of their professional obligation to provide effective services to all patients, they described internalized barriers that must be overcome.

I find it abnormal for a man to have sex with another man. It is both culturally and religiously unacceptable … Voices from religion or the community tell me that it is wrong. Professionally, I will have to handle that shock and look at possible ways of helping this person.

Participants reported a tendency to exhibit subtle forms of stigma and discrimination towards MSM patients, such as by maintaining body distance. Other times, HCWs explicitly showed disparaging treatment:

When they seek medical assistance in our facilities, the same providers will shout, ‘Look at him, he is telling me that he is having an anal STI; can you leave my room’. Instead of treating them with respect, they end up drawing their colleagues’ attention.

However, some HCWs challenged those who expressed personal prejudice towards MSM. Participants who had prior exposure to MSM sensitization argued that HCWs have a professional duty and societal obligation to provide non-prejudicial services to MSM.

We as health workers feel that MSM issues need not to be discussed, they are regarded as outcasts. How then would we come up with a constructive discussion about people whom we feel should not be in the society at first place? In my opinion, I think this is the biggest obstacle. If we accept these people and treat them as our clients, then it will be of great help to the society.

### Post-training discussion of HCWs’ attitudes, beliefs and behaviours

#### Recognition of MSM in Kenya

A pervasive theme in post-training focus groups was the explicit recognition of MSM in Kenya. Many reflected on how their prior denial of MSM behaviour, and their previous belief that anal sex among men was negligible in Kenya, had inhibited their capacity to provide services. Participants felt “empowered” by the training to address HIV and other health needs of MSM, as one stated:

I didn't ever believe that MSM were in existence but the training empowered me with a lot of knowledge and information on how to probe about issues of anal sex.

Participants described how the training enhanced their understanding of the complex interplay between homophobia, community denial of MSM and HIV transmission. Some advocated to local colleagues for the acceptance of MSM and educated them about the biological and behavioural circumstances that place MSM at heightened risk for HIV infection. One participant described:

I went and gave the feedback to my colleagues immediately after the training and some were as if they have never heard such a … They used to hear about it but they were not sure whether it was a real, whether such people exist. Therefore, I had to make them understand that the practice is in existence and that's nature.

#### Professional responsibilities as a health provider

During follow-up focus groups, participants described their professional responsibility to treat all patients with equity and respect. They endorsed a basic value of professionalism and treating MSM patients to the best of their ability. For many, this required a suspension of personal judgement in order to provide effective care:

As a professional, I am not supposed to segregate them, whether I support homosexuality or have a different perception or judgment. As a clinician, my duty is to treat without imposing my values on the patient. That's the positive thing I got from [the training program] and it's what I'm doing now.

Some described witnessing discriminatory actions towards MSM in their facilities or observing breaches in patients’ confidentiality. They reflected on how these experiences could foster distrust of HCWs and discourage MSM patients from seeking care when needed, thus perpetuating a cycle of HIV transmission. There was widespread consensus among group members that a concerted effort must be made to establish trusting rapport with MSM patients, and take extra care to employ discretion at all times. As one participant articulated:

I think the problem is that, the individuals we have attended to still want to see if they can trust us, if we can respect their privacy … As for now, it will take time because they are trying to internalize on our missions towards them and they will come out once they are convinced that you don't have an ill motive towards them.

During the follow-up focus groups, HCWs were asked to reflect upon and share their experiences, that is, work practices and attitudes towards MSM in their respective health facilities, and to reflect on strategies to change discriminatory actions towards MSM in their health facilities. Many participants stressed the importance of separating personal and religious values from professional ethics for the sake of HIV prevention in Kenya. While some felt the training had helped to normalize same-sex relations, others adamantly affirmed their aversion to MSM practices, but felt that they could compartmentalize their values to achieve the greater national public health goal.

The key message is almost the same. We are concentrating in breaking the transmission cycle among special groups, neglected groups. The bottom line is: we are not promoting but trying to help.

#### Sophisticated knowledge of risk in MSM

During the follow-up FGDs, participants exhibited a multifaceted understanding of the biological, behavioural and social influences that place MSM at risk for HIV. They described a better understanding of the processes through which unprotected anal sex contributes to HIV and STI transmission in both men and women, and the ways in which condoms and lubricants help to reduce risk. Moreover, many participants identified quality health education and counselling for MSM patients as integral to HIV prevention efforts in Kenya.

Participants generally recognized the societal pressures on MSM to conceal their sexual orientation, which MSM often mitigated by engaging in heterosexual relationships. They discussed the ways in which discrimination and lack of counselling and support services have hampered access to vital health services for MSM. The stigma endured by MSM in Kenya was consistently identified as an impediment to treatment, and many participants emphasized the need for HCWs to be thorough when examining MSM patients, who might not readily disclose their sexual practices:

I think it is good to do an examination as far as STI is concerned. A client might tell you that he is having a problem in his private parts. Such a client will openly tell you the exact location of the problem when you take the initiative to examine him.

Even if they go, they tend to be reluctant to disclose to clinicians that they are having anal infections. They end up getting the wrong medication and suffer in silence.

#### Ongoing challenges

Participants reflected on the challenges they will continue to face in affording appropriate health services to MSM. Many HCWs noted that time constraints and heavy workloads hinder their ability to deliver sensitive health services that MSM patients might require. Despite their desire to provide comprehensive health services to their MSM patients, some of the participants felt this was not always possible in practice:

Sometimes, as much as you would like to give all the attention to the client, there is a workload issue as other patients will be waiting. You may want to give the best, but the patients and the workload are too much.

Secondary stigma was considered an ongoing challenge, and HCWs tasked themselves to confront discrimination and stigma towards MSM expressed by their professional peers. Education, institutional support and other monitoring mechanisms were mentioned as powerful means for mitigating the effects of secondary stigma on service delivery to MSM patients, but all HCWs concurred with the fact that “it begins with openness, respect and understanding.”

HCWs emphasized the social challenges in targeting MSM for HIV preventative care. The marginalization of MSM, the belief that homosexuality runs contrary to cultural values and the fear of secondary stigma and resistance from fellow health professionals were regarded as impediments to the provision of care for MSM. As one participated stated:

Personally, I can say that my values have changed, though not 100%. I am not sure of the exact percentage, but I have positively changed. As much as I would like to live and exercise my changed values, there are still so many challenges in the society. I would like to give comprehensive care to MSM, but the society is too negative about them. This is a very big blow, given the fact that I am the only changed person.

In light of this, many participants noted the need for duplication opportunities for HCWs not yet trained on MSM sensitivity issues. They unanimously remarked that the on-line sensitivity course is very beneficial for skill development and in combination with follow-up group discussions allows for interpreting learning and connecting it to daily practice.

All participating HCWs advocated for community-wide sensitization campaigns to reduce stigma and encourage awareness of HIV risk in MSM, expressing the need for the community at large to engage in ongoing and productive dialogue in the struggle against HIV in Kenya.

## Discussion

This analysis provides qualitative insight into HCWs’ attitudes and experiences with MSM prior to and following a computer-facilitated MSM sensitization training programme [15] that will assist in amending the health workers’ e-learning sensitization course in future. Primary concerns expressed at baseline included fear of secondary stigma, lack of professional education about MSM, and negative influences of personal and social prejudice towards MSM. The nature of discussions changed following the programme, in which participants acknowledged the presence of MSM in their clinics, endorsed the need to treat MSM patients with high professional standards, and demonstrated sophisticated awareness of the social and behavioural risks for HIV among MSM. HCWs advocated for continuing the training and inviting more health professionals to participate, but cautioned that exclusively targeting MSM in the programme title could deter participation. HCWs also commented on the need for ongoing community dialogue about MSM, but recognized that community-level change will take time.

The attitudes and beliefs expressed by participants before versus after the training reveal many of the challenges to service provision for MSM patients. In general, participants’ personal beliefs about MSM and their endorsement of stigmatizing attitudes appear to have transformed following the programme. However, participants expressed ongoing concerns about secondary stigma and the influence of their professional peers’ negative judgements towards MSM patient and, by association, towards themselves. Professional peers’ negative and stigmatizing attitudes can potentially dilute the effects of the training on HCWs. Efforts to train larger cohorts of HCWs, establish networks of trained HCWs across different health clinics and change of institutional norms towards MSM patients may be necessary to counter the effects of secondary stigma and achieve sustainable improvements.

Limitations to this research must be acknowledged. First, due to the nature of qualitative methodology, participants’ responses might be influenced by social desirability and peer influences. Second, the findings reported here do not permit temporal, causal, or quantitative inferences, but indeed correspond with programme evaluation data reported in a related paper [14]. Third, due to the voluntary nature of participation, attitudes expressed by HCWs in this sample might not be representative of their peers and colleagues. Fourth, due to the active role of Kenyan health administrators in supporting this programme, the findings might not be replicable in areas where such support is lacking.

## Conclusions

This is the first known qualitative evaluation study of an MSM sensitivity training in Africa, which suggests that an online MSM sensitization training combined with group discussions can be a promising approach to improving health providers’ awareness, attitudes and beliefs about the health needs of MSM patients. Quantitative evaluation results, which show similar findings, are reported in a companion paper [14]. Further research is needed to evaluate the programme in a controlled study, and examine the implementation processes associated with successful programme delivery. Perspectives and service delivery outcomes from MSM patients would enhance understanding of the impact of this training on patient interaction. A particular strength of the intervention was the incorporation of two complementary training modalities – computer-facilitated training and group discussions – to provide didactic content as well as opportunities for group reflection, feedback and support. In general, participants noted a transformation in their personal attitudes and endorsement of stigma towards MSM following the training. However, their comments revealed the continued challenges to providing services to MSM in the context of broader societal homophobia and secondary stigma among their peers; their comments also highlighted challenges in recruiting larger groups of HCWs into the training due to anxiety around secondary stigma. Findings reported here can inform further adaptations of the training, particularly those domains that might influence HCWs’ willingness to participate and respond to the training (e.g., by emphasizing professional responsibilities of all health providers) and that diminish the effects of secondary stigma (e.g., by providing opportunities for ongoing support among trained HCWs). Findings underscore the need to view HCWs as an integral, but not absolute, component in addressing HIV and other health adversities among Kenyan MSM. Trained HCWs might benefit from continued opportunities for peer support, to counter feelings of professional isolation and motivate engagement in best practices. As participants noted, multi-component programmes and long-term commitments are necessary to achieve the goal of providing appropriate, effective services to MSM.
