# Supplementary material for: The epidemiology of HIV and prevention needs among men who have sex with men in Africa
Source: J Int AIDS Soc. 2013 Dec 2;16(4Suppl 3):18972. doi: 10.7448/IAS.16.4.18972 (PMC3852134; doi:10.7448/IAS.16.4.18972)
Supplement: Related texts

# Sub article: Experiences of Kenyan healthcare workers providing services to men who have sex with men: qualitative findings from a sensitivity training programme

**DOI:** 10.7448/IAS.16.4.18741

**Published:** 2013-12-02

**Authors:** Elise M van der Elst, Evans Gichuru, Anisa Omar, Jennifer Kanungi, Zoe Duby, Miriam Midoun, Sylvia Shangani, Susan M Graham, Adrian D Smith, Eduard J Sanders, Don Operario

**Affiliations:** 1 Centre for Geographic Medicine Research (Coast), Kenya Medical Research Institute, Nairobi, Kenya; 2 Provincial Medical Office, Ministry of Public Health and Sanitation, Mombasa, Kenya; 3 Desmond Tutu HIV Foundation, Cape Town, South Africa; 4 School of Public Health, Brown University Providence, RI, USA; 5 Department of Global Health, University of Washington Seattle, Washington, DC, USA; 6 Department of Public Health, University of Oxford, Headington, Oxford, UK

**Keywords:** on-line computer facilitated MSM sensitivity programme, healthcare worker, stigma, MSM, Kenya, HIV

## Abstract

**Introduction:**

Men who have sex with men (MSM) in Kenya are at high risk for HIV and may experience prejudiced treatment in health settings due to stigma. An on-line computer-facilitated MSM sensitivity programme was conducted to educate healthcare workers (HCWs) about the health issues and needs of MSM patients.

**Methods:**

Seventy-four HCWs from 49 ART-providing health facilities in the Kenyan Coast were recruited through purposive sampling to undergo a two-day MSM sensitivity training. We conducted eight focus group discussions (FGDs) with programme participants prior to and three months after completing the training programme. Discussions aimed to characterize HCWs’ challenges in serving MSM patients and impacts of programme participation on HCWs’ personal attitudes and professional capacities.

**Results:**

Before participating in the training programme, HCWs described secondary stigma, lack of professional education about MSM, and personal and social prejudices as barriers to serving MSM clients. After completing the programme, HCWs expressed greater acknowledgement of MSM patients in their clinics, endorsed the need to treat MSM patients with high professional standards and demonstrated sophisticated awareness of the social and behavioural risks for HIV among MSM.

**Conclusions:**

Findings provide support for this approach to improving health services for MSM patients. Further efforts are needed to broaden the reach of this training in other areas, address identified barriers to HCW participation and evaluate programme effects on patient and HCW outcomes using rigorous methodology.

## References

[1] Beyrer C, Baral SD, van Griensven F, Goodreau SM, Chariyalertsak S, Wirtz AL (2012). Global epidemiology of HIV infection in men who have sex with men. Lancet Infect Dis.

[2] Muraguri N, Temmerman M, Geibel S (2012). A decade of research involving men who have sex with men in sub-Saharan Africa: current knowledge and future directions. SAHARA J.

[3] Smith AD, Tapsoba P, Peshu N, Sanders EJ, Jaffe HW (2009). Men who have sex with men and HIV/AIDS in sub-Saharan Africa. Lancet.

[4] Sanders EJ, Graham SM, Okuku HS, van der Elst EM, Muhaari A, Davies A (2007). HIV-1 infection in high risk men who have sex with men in Mombasa, Kenya. AIDS.

[5] Sanders EJ, Okuku HS, Smith AD, Mwangome M, Wahome E, Fegan G (2013). High HIV-1 incidence, correlates of HIV-1 acquisition, and high viral loads following seroconversion among MSM. AIDS.

[6] Gouws E, Cuchi P, International Collaboration on Estimating HIV Incidence by Modes of Transmission (2012). Focusing the HIV response through estimating the major modes of HIV transmission: a multi-country analysis. Sex Transm Infect.

[7] Price MA, Rida W, Mwangome M, Mutua G, Middelkoop K, Roux S (2012). Identifying at-risk populations in Kenya and South Africa: HIV incidence in cohorts of men who report sex with men, sex workers, and youth. J Acquir Immune Defic Syndr.

[8] NACC (2009). Kenya national AIDS strategic plan 2009/10 – 2012/13: delivering on universal access to services.

[9] NASCOP, Ministry of Health (2012). MARPs surveillance report: unveiling new evidence for accelerating programming.

[10] WHO (2011). Prevention and treatment of HIV and other sexually transmitted infections among men who have sex with men and transgender people: recommendations for a public health apporach.

[11] Eden A, Taegtmeyer M (2003). Kenya national manual for training counsellors in voluntary counselling and testing for HIV.

[12] Ruiz JG, Mintzer MJ, Leipzig RM (2006). The impact of E-learning in medical education. Acad Med.

[13] Zimitat C (2001). Designing effective on-line continuing medical education. Med Teach.

[14] van der Elst EM, Smith AD, Gichuru E, Wahome E, Musyoki H, Muraguri N (2013). Men who have sex with men sensitivity training reduces homoprejudice
and increases knowledge among Kenyan healthcare providers in coastal Kenya. J Int AIDS Soc.

[15] Ritchie J, Spencer L, Bryman A, Burgess RG (1994). Qualitative data analysis for applied policy research. Analyzing qualitative data.

# Sub article: HIV among men who have sex with men in Malawi: elucidating HIV prevalence and correlates of infection to inform HIV prevention

**DOI:** 10.7448/IAS.16.4.18742

**Published:** 2013-12-02

**Authors:** Andrea L Wirtz, Vincent Jumbe, Gift Trapence, Dunker Kamba, Eric Umar, Sosthenes Ketende, Mark Berry, Susanne Strömdahl, Chris Beyrer, Stefan D Baral

**Affiliations:** 1 Center for Public Health and Human Rights, Department of Epidemiology, Johns Hopkins Bloomberg School of Public Health, Baltimore, MD, USA; 2 Department of Emergency Medicine, Johns Hopkins Medical Institute, Baltimore, MD, USA; 3 Department of Community Health, University of Malawi, College of Medicine, Blantyre, Malawi; 4 Centre for Global Health, Trinity College, Dublin, Ireland; 5 Centre for the Development of People, Blantyre, Malawi

**Keywords:** HIV, men who have sex with men (MSM), behavioural risks, stigma, Malawi, prevention

## Abstract

**Introduction:**

There are limited data characterizing the burden of HIV among men who have sex with men (MSM) in Malawi. Epidemiologic research and access to HIV prevention, treatment and care services have been traditionally limited in Malawi by criminalization and stigmatization of same-sex practices. To inform the development of a comprehensive HIV prevention intervention for Malawian MSM, we conducted a community-led assessment of HIV prevalence and correlates of infection.

**Methods:**

From April 2011 to March 2012, 338 MSM were enrolled in a cross-sectional study in Blantyre, Malawi. Participants were recruited by respondent-driven sampling methods (RDS), reaching 19 waves. Trained staff administered the socio-behavioural survey and HIV and syphilis voluntary counselling and testing.

**Results:**

Crude HIV and syphilis prevalence estimates were 15.4% (RDS-weighted 12.5%, 95% confidence interval (CI): 7.3–17.8) and 5.3% (RDS-weighted 4.4%, 95% CI: 3.1–7.6), respectively. Ninety per cent (90.4%, unweighted) of HIV infections were reported as being previously undiagnosed. Participants were predominantly gay-identified (60.8%) or bisexually identified (36.3%); 50.7% reported recent concurrent relationships. Approximately half reported consistent condom use (always or almost always) with casual male partners, and proportions were relatively uniform across partner types and genders. The prevalence of perceived and experienced stigma exceeded 20% for almost all variables, 11.4% ever experienced physical violence and 7% were ever raped. Current age >25 years (RDS-weighted adjusted odds ratio (AOR) 3.9, 95% CI: 1.2–12.7), single marital status (RDS-weighted AOR: 0.3; 95% CI: 0.1–0.8) and age of first sex with a man <16 years (RDS-weighted AOR: 4.3, 95% CI: 1.2–15.0) were independently associated with HIV infection.

**Conclusions:**

Results demonstrate that MSM represent an underserved, at-risk population for HIV services in Malawi and merit comprehensive HIV prevention services. Results provide a number of priorities for research and prevention programmes for MSM, including providing access to and encouraging regular confidential HIV testing and counselling, and risk reduction counselling related to anal intercourse. Other targets include the provision of condoms and compatible lubricants, HIV prevention information, and HIV and sexually transmitted infection treatment and adherence support. Addressing multiple levels of HIV risk, including structural factors, may help to ensure that programmes have sufficient coverage to impact this HIV epidemic among MSM.

## Introduction

Recent years have witnessed an increased awareness of the high burden of HIV among men who have sex with men (MSM) across the globe [1–3]. Emerging research suggests a greater transmission efficiency of HIV through receptive anal intercourse that is approximately 18 times higher than that of penile-vaginal sexual contact, increasing the risk among MSM for acquisition of HIV during sexual intercourse [4,5]. National HIV strategies and funding priorities, however, remain inequitable in many countries [6,7], particularly where structural factors, such as the criminalization of homosexuality, play critical roles in the level of research and programming available to MSM [8,9].

The HIV response in Malawi has focused on the prevention of heterosexual and vertical transmission of HIV to counteract the observed HIV incidence rates of 2–4% among adults in the 1990s. Today, the epidemic remains a generalized one, with an estimated 8.0% HIV prevalence among adult men [10]. Like neighbouring countries, assessments of specific risk factors for the acquisition and transmission of HIV, including transmission among MSM and other populations such as female sex workers, have been limited in the country [11]. Criminalization and stigmatization of homosexuality, as in other settings [8,12,13], are likely underlying factors for the limited targeted research and programming in the Malawian context.

To our knowledge, only two studies in Malawi have assessed sexual and social exposures that place MSM at risk for HIV infection. In 2008, our research team conducted a rapid HIV screening and socio-behavioural assessment among 201 MSM in Lilongwe and Blantyre, Malawi, as part of a comprehensive study across Southern African countries, including Namibia and Botswana, where homosexuality is criminalized [14]. This study documented HIV prevalence at approximately 21% [14], individual risk for HIV infections associated with increased age of the participant and inconsistent condom use [14] and high levels of violence and perceived stigma [15].

Another exploratory study examined socio-demographic and sexual behaviour characteristics among 97 MSM in central and southern Malawi. Although HIV prevalence was not assessed, the study found evidence of high-risk behaviours such as inconsistent condom use (32.5%), transactional sex (23.7%), low exposure to HIV messaging (17.5%) and a low history of HIV testing (58.8% ever tested) [16]. Although these studies were the first and only to elucidate the socio-behavioural factors among MSM in Malawi, they were rapid assessments and served to highlight areas for future research and prevention.

In response to the global epidemic of HIV among MSM, combination prevention packages have been put forth as a key method to curb the HIV epidemic among MSM [17,18]. To inform the content and scale of a combination HIV prevention intervention (CHPI) for MSM in Malawi, we conducted this study to estimate HIV prevalence, characterize associations of prevalent HIV infections, and evaluate barriers and facilitators to uptake of HIV prevention services among MSM in Blantyre, Malawi. Research was conducted in collaboration with a community-based organization, the Centre for the Development of People (CEDEP), and the Malawi College of Medicine, University of Malawi.

## Methods

### Study population and setting

This cross-sectional assessment was conducted from August 2011 to March 2012 in Blantyre, Malawi. Eligibility requirements for participation included being born male, being aged 18 years or older, being fluent in Chichewa or English, having reported anal sex with another man in the last 12 months, having no prior participation in this study, and providing informed verbal consent to participate. Study activities were conducted in private rooms of CEDEP's study site and implemented by staff from CEDEP, which provides HIV prevention activities for MSM in Malawi, and the Malawi College of Medicine. All staff members were trained in confidentiality and human subjects protection, qualitative and survey research and respondent-driven sampling (RDS) methods.

### Recruitment method

Participants were recruited via RDS, a chain recruitment method often used to achieve more representative samples of hard-to-reach populations [19]. Recruitment began with 10 purposively selected seeds who were each provided with three study-specific coupons with which to recruit peer MSM from their social network into the study. Initiation of seeds was staggered over the duration of the study, taking into consideration potential propagation failure by some seeds and eventual die-out of the chains. Seeds were recruited from the pool of MSM who were involved in local HIV prevention programmes or had participated in prior formative research, and they were selected to represent a range of characteristics, including age, education, employment and sexual identity. Individuals who were recruited by seeds and enrolled in the study were then provided with three study coupons for further recruitment of peers. This process continued until the target sample size was reached. Participants were reimbursed K1500.00 (US$5.00) for transportation costs for participation in the study and K500.00 (US$1.50) for recruitment of each peer into the study. A full description of traditional RDS methodology can be found elsewhere [20]. Netdraw software (Analytic Technologies) was used to monitor RDS recruitment [21].

### Sample size

The sample size calculation was powered on the assumed 85% effectiveness of condoms in preventing the transmission of HIV during intercourse [22]. Thus, we assumed that approximately 30% of the sample would be consistent condom users and that they would be 85% less likely to be living with HIV than the 70% who are not consistent condom users. Based on previous research, we estimated that the HIV prevalence in the population would be about 20%, equating to 27% among non-consistent condom users, 4% among consistent condom users and a 30% population prevalence of consistent condom usage. We used a design effect of 1.5 [23], power set at 80% and a significance level of 95%, which resulted in an effective sample size estimate of 345 participants for which we had targeted 350 MSM.

### Measures

Participation included a structured survey instrument and a biological assessment of HIV and syphilis. Trained interviewers administered surveys in the Chichewa language, following pilot testing. Measures included sociodemographic characteristics, substance use, mental health and depression symptoms, sexual relationships and disclosure of orientation or sexual practices to family and peers. Measures of sexual practices included practices with men and women, including anal, oral and vaginal sex; number of sexual partners and partner characteristics; concurrent relationships, defined as “two sexual partnerships at the same time or two ongoing sexual partnerships (male and/or female genders)”; and transactional sex (purchased or sold). We measured HIV knowledge and prevention, including aspects of condom and condom-compatible lubricant use; HIV testing and counselling exposures; and access to and uptake of health services. Human rights measures included experiences of physical and sexual violence, experienced and perceived stigma and history of imprisonment. Recall periods were lifetime, last 12 months or both, and they are specified in the results tables. The development of survey questions, recruitment methods, coupons and study procedures was informed by formative research that was conducted in May–July, 2011 [24].

### Biologic specimens

Following completion of the interview, participants proceeded to HIV and syphilis testing. A trained nurse from the College of Medicine conducted HIV testing, blood specimen collection and pre- and post-test counselling. Blood-based rapid HIV tests were conducted simultaneously using the Determine^®^ HIV-1/2 and Uni-Gold rapid HIV tests (manufactured, respectively, by Inverness Medical, Chiba, Japan; and Trinity Biotech, Bray, Ireland). Participants received their HIV test results and post-test counselling within 15 minutes of collection. Separate specimens were collected for confirmatory testing of discrepant or indeterminate HIV rapid tests using Western blot in accordance with Malawian National Guidelines [25]. Approximately 5 ml of whole blood was collected for TPHA (treponema pallidum haemagglutination test) syphilis testing (Bio-rad, Hercules, CA, USA). Resource constraints prevented the use of the nontreponemal test, which would differentiate active from past syphilis infections. Confirmatory HIV and syphilis tests were analysed at the Malawi College of Medicine laboratory in Blantyre. Participants returned within one to two weeks to receive their syphilis test results. Participants testing positive for HIV and/or syphilis were referred to the local hospital or to the Johns Hopkins antiretroviral therapy and sexually transmitted infection clinic located at Queens Hospital. Participants were provided with information about local health centres that had, as part of the study, received training for the provision of services to MSM. One trained team member (EU) provided counselling services to MSM participants as needed.

### Analysis

Johns Hopkins University conducted secondary data analysis of collected data. The principal outcome of interest was HIV diagnosis with predictor variables that included demographics (education, age, number of children and marital status), socio-economic variables, lifetime residence in urban or rural locations, recent sexual behaviours, human rights exposures, HIV prevention methods, health-seeking behaviour and laboratory markers of syphilis infection. Variable-specific individualized weights, which take into account estimates for individual degrees, were computed by a data-smoothing algorithm using RDS for Stata [26]. The estimated weights were used in univariate RDS-weighted analyses. HIV status individualized weights were used in the bivariate and multivariate RDS-weighted analyses. Bootstrapped 95% confidence intervals (CIs) were computed using 1000 iterations for the estimated descriptive statistics [23]. Homophily, a measure of to what extent respondents prefer to recruit from their own group rather than at random, was estimated where appropriate and presented in the results in Table 1.

**Table 1:** Demographics, identity and health characteristics of RDS-recruited MSM in Blantyre, Malawi (August 2011–March 2012; *n=*338)

		Crude		RDS weighted		
			
Variable	Categories	*n*	(%)	(%)	(95% CI)	Homophily
**Demographics**						
Age	18–25 years	192	56.8	58.5	[50.2–66.8]	0.261
	≥26 years	146	43.2	41.5	[33.2–49.8]	0.231
Highest level of education (completed)	Less than secondary	146	43.2	46.3	[38.9–53.8]	0.115
	Secondary or higher	192	56.8	53.7	[46.2–61.1]	0.147
Employment status	Unemployed	158	46.8	51.7	[44.2–59.3]	0.008
	Employed	136	40.2	36.8	[29.6–44.1]	0.153
	Student	44	13.0	11.5	[0.74–15.5]	0.044
Gender identity	Male	263	77.8	80.2	[74.8–85.6]	0.008
	Female	65	19.2	17.0	[11.8–22.1]	0.153
	Transgender	10	2.9	2.8	[0.8–4.9]	0.044
Sexual orientation	Gay or homosexual	210	62.3	60.8	[53.6–68.1]	0.092
	Bisexual	125	37.1	36.3	[29.1–43.4]	0.070
	Heterosexual or straight	2	0.6	2.9	[0.0–6.7]	−0.029
Marital status (with a woman)	Married or cohabiting	54	16.0	16.2	[9.7–22.7]	0.196
	Single, divorced or separated	284	84.0	83.7	[77.4–90.1]	0.255
Number of children	None	285	84.6	84.9	[78.2–91.6]	0.224
	One or more	52	15.4	15.1	[0.8–21.8]	0.142
Type of location where majority of life was spent	Urban	279	82.5	78.3	[72.1–84.6]	0.231
	Rural	59	17.5	21.7	[15.4–27.9]	0.050
**Health indicators**						
HIV diagnosis	Negative	286	84.6	87.5	[82.2–92.8]	−0.090
	Positive	52	15.4	12.5	[7.2–17.8]	0.022
Syphilis diagnosis	Negative	319	94.7	95.6	[92.9–98.3]	−0.195
	Positive	18	5.3	4.4	[1.7–7.1]	0.009

To develop the statistical model, we first carried out bivariate analysis to assess the association of HIV status with the control variables (Table 4). Demographic variables were included in the multivariate logistic regression model regardless of the estimated strength of their bivariate association with HIV status. Selected non-demographic variables were included in the multivariate model if the chi-square *p*-value of association with HIV status was ≤0.25. Some variables such as HIV testing were not included in the multivariate model due to collinearity. The final model, presented in Table 4, includes demographics and variables left in the final model following goodness-of-fit tests. All statistical analyses were conducted using Stata 12.1 [27]. Results provided in the text report RDS-weighted estimates (unless otherwise specified), while tables display unweighted and RDS-weighted estimates as well as 95% CIs for weighted estimates. Table 4 presents the results of bivariate and final multivariate analyses, including unweighted and RDS-weighted odds ratios (ORs) and adjusted ORs (AORs) for the final multivariate model.

### Human subjects

Research activities were reviewed and approved by the Malawi College of Medicine Ethics and Research Committee and for secondary analysis by the Johns Hopkins Bloomberg School of Public Health Institutional Review Board.

## Results

A total of 338 MSM (including original seeds) were recruited via RDS and enrolled into the study, reaching 19 waves of recruitment. Out of 10 seeds, five recruited participants; one recruitment chain was responsible for the recruitment of 70% of the study population. Three recruitment chains are reflective of later seed initiation. A total of 706 coupons were distributed with a return rate of 48%. The majority of participants reported recruitment by a friend (60.5%) or sex partner (32.3%). Median MSM network size was 8 (range 1 to 800). Figure 1 displays the RDS recruitment diagram, highlighted by HIV diagnosis. We used this method to monitor recruitment and to assess whether HIV diagnosis inhibited recruitment, which appeared not to be the case.

**Figure 1:** RDS recruitment diagram from 338 MSM recruited in Blantyre, Malawi. Large nodes=seeds; small nodes=recruits; grey=HIV+ according to Determine rapid HIV test; black=HIV- on Determine rapid HIV test.

Participants’ median age was 25.1 years (range: 18 to 49). Based on RDS-weighted estimates, 51% were unemployed and 21.6% had ever been in jail or prison. Eighty per cent identified as male gender. Sixty-one per cent identified as gay or homosexual, and 36.3% reported bisexual identity. Sixteen per cent were married or cohabitating with a woman. Table 1 displays sociodemographic characteristics.

The crude prevalence of HIV infection in this population was 15.4%, with an RDS-weighted estimate of 12.5% (Table 1). The majority, 90.4% (unweighted), of these infections were previously undiagnosed; these participants had either self-reported as negative or reported never being tested for HIV infection. Positive syphilis diagnosis was low at 4.4%.

Table 2 presents sexual practices, partner characteristics and social exposures. Only 18.1% had ever disclosed sexual practices or orientation to their family, and equally few (18.9%), had ever disclosed to a health provider. Participants reported a mean of four male partners (range: 1 to 50), and 31% reported having female partners in the last 12 months. Half of the population reported concurrent sexual relationships, and, among those in a relationship, 61.3% believed their partner was also involved in a concurrent relationship. Prevalence of perceived and experienced stigma and discrimination exceeded 20% of the population for almost all variables, 7.0% were ever raped and 11.4% had ever experienced physical violence.

**Table 2:** Sexuality, partnerships and risk exposures of RDS-recruited MSM in Blantyre, Malawi (August 2011–March 2012; *n=*338)

		Unweighted		RDS weighted	
			
Variable	Categories	*n*	(%)	(%)	(95% CI)
Ever disclosed sexual practice to family	Yes	69	20.4	18.1	[12.9–23.2]
Ever disclosed sexual practice to health provider	Yes	70	20.8	18.9	[13.3–24.5]
Gender of first sex partner	Male	226	67.1	69.4	[62.5–76.3]
	Female	111	32.9	30.6	[23.7–37.5]
Age at first sex with another man	<16 Years old	55	16.3	14.9	[8.9–21.0]
	16–20 Years old	174	51.6	53.1	[45.5–60.8]
	21–25 Years old	74	22.0	22.4	[16.1–28.8]
	>25	34	10.1	9.6	[5.3–13.9]
Sexual partners in last 12 months: mean (range)	Male partners [*n=*334]	3.8	(1–50)	N/A	N/A
	Female partners [*n=*107]	2.9	(1–20)	N/A	N/A
Concurrent relationships, last 12 months*	None	160	47.3	49.5	[42.3–56.8]
	Yes, two or more male and/or female partners	178	52.7	50.7	[43.3–57.9]
Believes partner has concurrent relationship* (*n=*76)	Yes	59	77.6	61.3	[30.1–90.9]
Normally has sex with men in a private home	Yes	224	66.3	67.6	[60.5–74.8]
… in bar or clubs	Yes	98	29.0	26.1	[20.2–32.1]
… in a hotel	Yes	78	23.1	20.1	[14.2–26.0]
**Social exposures**					
Ever felt excluded from family gatherings	Yes	87	25.7	26.1	[20.0–32.2]
Ever felt rejected by friends	Yes	118	35.0	27.2	[20.7–34.]
Feels there are safe places to go to socialize with other MSM	Yes	223	66.8	66.0	[59.1–72.9]
Ever felt afraid to seek health services	Yes	68	20.1	21.5	[15.1–27.9]
Ever been in jail or prison	Yes	74	22.1	21.6	[14.1–29.1]
Ever experienced physical violence	Yes	40	11.8	11.4	[6.1–16.8]
Ever raped (*N*=337)	Yes	26	7.7	7.0	[3.5–10.5]

* Concurrent sexual partnership: two or more male or female sexual partners during the same time period.

Responses to questions on knowledge of HIV risk, prevention methods and practices are reported in Table 3. Approximately half of the participants with casual male partners (*n*=256) reported using condoms always or almost always with casual male partners; frequencies were approximately similar across partner type (e.g., casual or main) and partner gender. Approximately 44.3% had never been tested for HIV. Among those ever tested for HIV infection, 45.5% (unweighted) had not been tested within the last year.

**Table 3:** Knowledge of HIV risk, prevention methods and practices of RDS-recruited MSM in Blantyre, Malawi (August 2011–March 2012; *n=*338)

		Unweighted		RDS weighted	
			
Variable	Categories	*n*	(%)	(%)	(95% CI)
Condom use in last sex with *main* male partner (*n=*316)	Yes	119	63.0	59.6	[51.7–67.4]
Condom use in last sex with *casual* male partner (*n=*256)	Yes	174	68.0	66.3	[57.4–75.2]
Condom frequency with *main* male partners (*n=*316)	Never	44	14.1	15.3	[9.9–20.6]
	Almost never	52	16.7	18.4	[11.7–25.0]
	Sometimes	63	20.2	19.9	[13.8–25.9]
	Almost always	41	13.1	10.8	[6.3–15.2]
	Always	112	35.9	35.7	[27.9–43.6]
Condom frequency with *casual* male partners (*n*=256)	Never	34	13.3	15.6	[9.5–21.7]
	Almost never	30	11.7	14.0	[6.2–21.9]
	Sometimes	53	20.7	21.4	[14.1–28.6]
	Almost always	28	10.9	4.8	[1.8–7.8]
	Always	111	43.4	44.2	[34.5–53.8]
Condom use at last sex with *main* female partner (*n=*101)	Yes	45	44.5	50.3	[23.9–76.6]
Condom use at last sex with *casual* female partner (*n=*71)	Yes	46	64.8	58.2	[27.5–88.8]
Condom frequency with *main* female partners	Never	25	25.0	22.5	[2.6–42.4]
	Almost never	27	27.0	27.3	[6.5–48.2]
	Sometimes	15	15.0	3.4	[0.0–15.4]
	Almost always	12	12.0	7.7	[0.0–18.0]
	Always	21	21.0	39.1	[8.3–69.8]
HIV testing (ever; *n=*336)	Never	134	39.9	44.3	[37.0–51.6]
	Once	123	36.6	32.8	[26.4–39.2]
	More than once	79	23.5	22.9	[17.3–28.6]
Considered vaginal sex most “risky” type of sex	Yes	180	53.4	52.8	[45.7–59.9]
Considered anal sex most “risky” type of sex	Yes	59	17.5	15.4	[10.0–20.8]
Considered both equally “risky” types of sex	Yes	119	35.2	35.6	[28.6–42.6]
Ever received information about HIV prevention for sex with men	Yes	75	22.5	18.8	[12.9–24.7]
Ever received information about HIV prevention for sex with women	Yes	183	54.3	53.7	[46.3–61.0]
Knowledge of risk related to positioning	Insertive (top)	56	16.7	15.2	[10.2–20.3]
	Receptive (bottom)	112	33.4	38.0	[30.7–45.3]
	Both carry equal risk	167	49.9	46.7	[39.8–53.6]
Considers safest lubricants to use during anal sex	Petroleum jelly or Vaseline	133	43.6	49.7	[41.2–58.5]
	Water-based lubricant	43	14.1	14.5	[8.0–21.1]
	Others or none	130	42.5	35.6	[27.2–43.9]
Lubricant use	Petroleum jelly or Vaseline	149	45.3	48.3	[40.1–56.0]
	Water-based lubricant	74	22.5	25.2	[18.1–32.3]
	Others or none	106	32.2	26.5	[19.8–33.2]

Several sociodemographic variables were associated with HIV infection in the bivariate analysis (Table 4). These included current age >25 years (RDS-weighted OR: 8.1, 95% CI: 2.9–22.2), single marital status (RDS-weighted OR: 0.2, 95% CI: 0.1–0.4) and having more than one child (RDS-weighted OR: 5.3, 95% CI: 1.8–15.6). Age <16 years at first sex with a man was associated with HIV infection (RDS-weighted OR: 1.7, 95% CI: 0.4–7.5). Considering water-based lubricants to be the safest lubricant (RDS-weighted OR: 0.9, 95% CI: 0.2–3.6) and use of water-based lubricant (RDS-weighted OR: 0.6, 95% CI: 0.2–2.0) were also marginally protective.

**Table 4:** Bivariate and multivariate associations of HIV infection among RDS-recruited MSM in Blantyre, Malawi (August 2011–March 2012; *n=*338)

		HIV negative		HIV positive		Total			OR [unweighted]		OR [RDS weighted]		Multivariate AOR [unweighted]		Mulitvariate RDS-weighted AOR	
									
Variable	Categories	No.	(%)	No.	(%)	No.	(%)	*p*	Estimate	(95% CI)	Estimate	(95% CI)	Estimate	(95% CI)	Estimate	(95% CI)
**Socio-demographics**																
Age	18–25	181	94.2	11	5.7	192	56.8	<0.001	1		1		1		1	
	≥26	105	71.9	41	28.1	146	43.2		6.4	[3.2–13.0]	8.1	[2.9–22.2]	3.2**	[1.3–7.9]	3.9**	[1.2–12.7]
Education	Less than secondary	126	86.3	20	13.7	146	43.2	0.454	1		1		1		1	
	Secondary or higher	160	83.3	32	16.7	192	56.8		1.3	[0.6–2.3]	0.8	[0.3–2.0]	0.6	[0.3–1.4]	0.5	[0.2–1.6]
Employment status	Unemployed	140	88.6	18	11.4	158	46.8	<0.001	1		1		1		1	
	Employed or self-employed	103	75.7	33	24.3	136	40.2		2.5	[1.3–4.7]	2.1	[0.8–5.8]	2.5*	[1.1–5.4]	2.4	[0.9–6.7]
	Student	43	84.7	1	2.3	44	13.0		0.2	[0.1–0.2]	0.2	[0.0–1.5]	0.3	[0.0–2.8]	0.5	[0.0–6.4]
Marital status (with a woman)	Not single, or widowed	35	64.8	19	35.2	54	16.0	<0.001	1		1		1		1	
	Single or never married	251	88.4	33	11.6	284	84.0		0.2	[0.1–0.5]	0.2	[0.1–0.4]	0.7	[0.2–2.0]	0.3*	[0.1–0.8]
Number of children	None	253	88.8	32	11.2	285	84.6	<0.001	1		1		1		1	
	≥1 child	32	61.5	20	38.5	52	15.4		4.9	[2.5–9.6]	5.3	[1.8–15.6]	2.2	[0.7–6.5]	1.2	[0.4–4.1]
Place where respondent grew up	Urban	233	83.5	46	16.5	279	82.5	0.222	1		1		1		1	
	Rural	53	89.8	6	10.2	59	17.5		0.6	[0.2–1.4]	0.7	[0.2–2.4]	0.3	[0.1–1.0]	0.4	[0.1–1.7]
**Sexuality, partnerships and risk exposures**																
Sexual orientation	Gay or homosexual	178	84.8	32	15.2	210	62.3	0.398	1		1					
	Bisexual	106	84.8	19	15.2	125	37.1		1.0	[0.5–1.9]	1.1	[0.5–2.6]				
	Straight or heterosexual	1	50.0	1	50.0	2	0.6		5.6	[0.4–91.2]	24.8	[1.5–420.1]				
Ever disclosed sexual practice to health provider	No	228	85.4	39	14.6	267	79.2	0.414	1							
	Yes	57	81.4	13	18.6	70	20.8		1.3	[0.7–2.7]	1.1	[0.4–3.2]				
Age at first sex with another man	<16 Years old	42	76.4	13	23.6	55	16.3	<0.001	1.6	[0.7–3.8]	1.7	[0.4–7.5]	2.7	[0.9–7.5]	4.3*	[1.2–5.0]
	16–20 Years old	160	92.0	14	8.0	174	51.6		0.5	[0.2–1.0]	0.3	[0.1–0.9]	0.8	[0.3–2.2]	0.6	[0.2–2.2]
	21–25 Years old	62	83.9	12	16.2	74	22.0		1		1		1		1	
	>25	21	61.8	13	38.2	34	10.0		3.2	[0.1–0.4]	3.4	[1.0–11.4]	2.2	[0.7–6.5]	2.6	[0.8–8.6]
Normally has sex with men … in a private home	No	90	79.0	24	21.0	114	33.7	0.039	1		1					
	Yes	196	87.5	28	12.5	224	66.3		0.5	[0.3–1.0]	0.8	[0.3–1.9]				
… in bars or clubs	No	208	86.8	32	13.3	240	71.0	0.102	1							
	Yes	78	79.6	20	20.4	98	29.0		1.7	[0.9–3.1]	0.9	[0.4–2.03]				
… in a hotel	No	225	86.5	35	13.5	260	76.9	0.074	1							
	Yes	61	78.2	17	21.8	78	23.1		1.8	[0.9–3.4]	0.9	[0.4–2.2]				
Condom use at last sex with main male partner	Yes	103	88.0	14	12.0	117	37.0	0.221	1		1					
	No	165	82.9	34	17.1	199	63.0		1.5	[0.8–3.0]	0.9	[0.3–2.6]				
**Social and human rights contexts**																
Feels there are safe places to go to socialize with other MSM	No	88	79.3	23	20.7	111	33.2	0.067	1		1					
	Yes	194	87.0	29	13.0	223	66.8		0.6	[0.3–1.0]	0.4	[0.2–1.1]				
Ever felt afraid to seek health services	No	229	84.8	41	15.2	270	79.9	0.840	1		1					
	Yes	57	83.8	11	16.2	68	20.1		1.1	[0.5–2.2]	0.7	[0.3–2.2]				
Ever in jail	No	227	87.0	34	13.0	261	77.9	0.018	1		1					
	Yes	56	75.7	18	24.3	74	22.1		2.2	[1.1–4.1]	1.4	[0.6–3.4]				
Raped (*N*=337)	No	266	85.5	45	14.5	311	93.3	0.090	1		1		1		1	
	Yes	19	73.1	7	26.9	26	7.7		2.2	[0.9–5.5]	0.6	[0.2–2.0]	2.9	[0.9–9.5]	15	[0.2–9.6]
**Knowledge of HIV risk, prevention methods and practices by HIV diagnosis**																
HIV testing (ever; *n*=336)	Never	113	84.3	21	15.7	134	39.9	0.938	1		1					
	Once	105	85.4	18	14.6	123	36.6		0.9	[0.5–1.8]	0.8	[0.3–2.2]				
	More than once	66	83.5	13	16.5	79	23.5		1.1	[0.5–2.3]	0.5	[0.2–1.5]				
Last 12 months (of ever tested; *N=*202)	Never	79	83.2	16	16.8	95	45.5	0.854	1		1					
	Once	74	86.0	12	14.0	86	41.1		0.8	[0.4–1.8]	0.5	[0.1–1.8]				
	More than once	24	85.7	4	14.3	28	13.4		0.8	[0.3–2.7]	0.5	[0.1–2.3]				
Considered anal sex most “risky” type of sex	No	235	84.2	44	15.8	279	82.5	0.669	1		1					
	Yes	51	86.4	8	13.6	59	17.5		0.8	[0.4–1.9]	0.4	[0.1–1.1]				
Considers safest lubricants to use during anal sex	Petroleum jelly or Vaseline	119	91.5	14	8.49	133	43.5	0.102	1		1					
	Water-based lubricant	104	83.5	26	16.4	130	42.5		1.7	[0.6–4.4]	0.9	[0.2–3.6]				
	Others or none	36	86.7	7	13.2	15	4.9		2.1	[1.1–4.3]	1.2	[0.4–3.2]				
Lubricant use	Petroleum jelly or Vaseline	132	88.6	17	11.4	149	45.3	0.124	1		1		1		1	
	Water-based lubricant	84	79.3	22	20.7	106	32.2		1.5	[0.7–3.3]	0.6	[0.2–2.0]	1.8	[0.7–4.6]	0.9	[0.3–2.4]
	None or others	62	83.8	12	16.2	74	22.5		2.0	[1.1–4.1]	1.3	[0.5–3.4]	2.8*	[1.2–6.5]	2.7	[0.9–8.4]
How many men have you had anal or oral sex with in the past 12 months?													0.9	[0.7–1.1]	0.8	[0.6–1.1]

Exponentiated coefficients; 95% confidence intervals in brackets

* *p*<0.05

** *p*<0.01

analysis sample=318.

The final multivariate model included age, marital status, number of children, knowledge of risk related to positioning (insertive or receptive anal intercourse), lubricant type used, age of first sex with another man, history of rape, number of male anal or oral sex partners and other known confounders such as employment, education and syphilis diagnosis. Of these, current age >25 years (RDS-weighted AOR 3.9, 95% CI: 1.2–12.7), single marital status (RDS adjusted AOR: 0.3, 95% CI: 0.1–0. 8) and age of first sex with a man <16 years (RDS adjusted AOR: 4.3, 95% CI: 1.2–15.0) were independently associated with HIV infection.

## Discussion

This cross-sectional study, the most comprehensive yet conducted among MSM in Malawi, describes the high prevalence of HIV infection as well as the limited uptake of HIV prevention, testing and care services among MSM in Blantyre, Malawi.

HIV prevalence was high among MSM, and nearly all HIV infections were among men who reported being unaware of their status of HIV infection. Only slightly more than half of the population reported ever having been tested, and only half of those were within the last year, potentially explaining this level of undiagnosed HIV infections. Knowing one's status is increasingly more important for HIV prevention. Novel HIV interventions, including pre-exposure prophylaxis for HIV-uninfected men [28,29] and early treatment for people living with HIV [30], represent a new generation of HIV-status-dependent interventions. Awareness of one's HIV status has also been associated with decreased self-reported prevalence of high-risk sexual practices that are associated with HIV transmission [31]. Recent US Centers for Disease Control guidelines have suggested more frequent testing (every 3 or 6 months) based on individual assessment of sexual risk behaviours [32], representing a strategy which may also be relevant for MSM in Malawi.

Young age of first sexual intercourse with a man (<16 years) was independently associated with HIV infection in this population, with almost four times greater odds of HIV infection compared to the referent group. This association may suggest biologic susceptibility during physical development, high-risk sexual behaviours and lack of access to or low use of condoms at a young age, and/or an association with duration of sexual activity [33,34]. Likewise, the association of prevalent HIV infection with older current age may be due to higher cumulative risk exposures for acquisition of HIV. However, estimating the duration of sexual activity is challenging as sexual behaviours are not static, but vary across the life course and as partnerships change [34]. While study-related factors such as low power and potential misclassification of behaviours may partially explain insignificant findings, broader factors such as high background prevalence of HIV in the MSM population [35], biologic susceptibility of rectal mucosa [36] and network-level characteristics may also be more determinative in driving HIV transmission and acquisition risks among these men [35,37]. Nonetheless, this study described a population reporting high-risk behaviours, suggesting the need to ensure accessibility to HIV prevention interventions across ages [38–40]. These behavioural risks, combined with the high proportion of undiagnosed HIV infection in this study, also suggests there is a high likelihood of someone with a high viral load within a sexual network potentially driving onward transmission [41,42]. Future research among MSM in Malawi to better characterize different risk strata among MSM, including reported sexual practices and sexual network characteristics, is needed to better tailor the content of interventions and enable the identification of infection.

While addressing the unique needs of the individual is fundamental, stigma and discrimination have been reported commonly as structural barriers to the uptake of services [43,44]. Experienced and perceived stigma as well as other physical and sexual violence were common among MSM in this study, consistent with earlier quantitative and qualitative studies in Malawi [15,24]. Stigma has been shown to limit health-seeking behaviours and use of HIV prevention methods, disclosure of sexual practices to health providers, and providers’ liberty to provide services to MSM [14,15,24,45,46]. The need to keep male-male partnerships hidden may lead to more frequent, short-term relationships and increased high-risk behaviours [24]. Such responses to stigma and social pressures may explain the high prevalence of concurrency, the high-risk sexual practices reported in this study, the proportion of men who are married or cohabitating with women and the protective effect of single marital status in this analysis. Addressing these social issues is a necessity for improving access to and uptake of effective HIV prevention interventions [8].

Taken together, these data demonstrate that MSM are an underserved and important population for targeted HIV prevention interventions; MSM may specifically benefit from the CHPI that we subsequently developed based on the quantitative results presented here. Mathematical models have shown that high levels of coverage among MSM (i.e., 60–80%) are required to change the trajectory of the HIV epidemic among MSM, and such findings are likely to be relevant in Malawi [2,47,48]. To address low coverage of prevention options among Blantyre MSM and the limitations of single interventions, comprehensive packages of interventions that include behavioural, biomedical and structural approaches may be the most effective approach to reducing HIV among MSM [17]. Such interventions may be feasible in Malawi and may have the same positive impact on sexual transmission that has been observed in other settings, including countries where same-sex practices are criminalized [30,47,49,50].

The method of intervention delivery is critical to the success of HIV prevention programmes in the context of complex social environments. The success of RDS recruitment suggests that interventions leveraging existing peer networks, which have demonstrated efficacy in other settings [51,52], may serve as a feasible approach to providing and supporting HIV prevention interventions for MSM in Malawi. Addressing stigma in healthcare settings may improve provider-patient relationships, facilitate disclosure and meaningful discussion of risk practices, and foster linkage to HIV testing and care [53]. While the subsequent feasibility assessment of the CHPI programme for MSM in Blantyre will be informative for understanding how a comprehensive package may address individual social and behavioural risks for HIV infection, broader social acceptance of MSM may take time and remains a crucial step towards improving the health status of MSM and thus all Malawians [8].

### Limitations

The cross-sectional nature of this study limits the investigation of temporal associations and thus the causality of the exposures and HIV-related outcomes. Additional limitations are related to the ability to fully assess correlates of prevalent HIV infection through behavioural surveys, which may have limited the significance of findings in this study. This may also be amplified by the potential response bias related to asking sensitive questions of a highly stigmatized population. We attempted to address these limitations to the fullest extent possible, including using lifetime and recent recall periods, developing survey questions based on formative research and prior research studies among MSM, and taking measures to ensure the confidentiality and privacy of participants and inform them of these privacy control measures. This study provides equipoise for prospective cohorts of MSM to better characterize HIV incidence and, ultimately, appropriately powered HIV prevention and implementation science studies to assess effective strategies in HIV risk reduction.

There are limitations associated with the use of RDS methodology [54]. Specifically, there is debate around appropriate interpretation of the measures of association and optimal strategies to handle variance in studies using RDS. For example, use of water-based lubricants appeared to be independently protective in the model that did not adjust for RDS, but this association is no longer significant with the introduction of the increased variance associated with RDS adjustment in the model. Despite these analytic challenges, RDS represents a relevant sampling strategy to obtain a diverse sample of a hidden population in the absence of a sampling frame or a sufficient number of established venues [19].

## Conclusions

This study presents an assessment of individual, sexual-network and structural factors and their relationship with prevalent HIV infections among MSM in Blantyre, Malawi. The burden of HIV is high among these men, with the vast majority apparently unaware of their HIV status. Approaches rooted in engagement in the continuum of HIV care will be central moving forward in Malawi [55]. Addressing stigma and discrimination should also represent a core programmatic and policy element of the HIV response, to ensure that these efficacious approaches are translated into effective ones and to optimize the health of MSM living with HIV in Malawi while preventing onward HIV transmission.

## References

[1] Beyrer C, Baral S, Walker D, Wirtz A, Johns B, Sifakis F (2010). The expanding epidemics of HIV-1 among men who have sex with men in low and middle income countries: diversity and consistency. Epidemiol Rev.

[2] Beyrer C, Baral SD, van Griensven F, Goodreau SM, Chariyalertsak S, Wirtz AL (2012). Global epidemiology of HIV infection in men who have sex with men. Lancet.

[3] Muraguri N, Temmerman M, Geibel S (2012). A decade of research involving men who have sex with men in sub-Saharan Africa: current knowledge and future directions. SAHARA J.

[4] Baggaley RF, White RG, Boily MC (2010). HIV transmission risk through anal intercourse: systematic review, meta-analysis and implications for HIV prevention. Int J Epidemiol.

[5] Jin F, Jansson J, Law M, Prestage GP, Zablotska I, Imrie JC (2010). Per-contact probability of HIV transmission in homosexual men in Sydney in the era of HAART. AIDS.

[6] Grosso AL, Tram KH, Ryan O, Baral S (2012). Countries where HIV is concentrated among most-at-risk populations get disproportionally lower funding from PEPFAR. Health Aff (Millwood).

[7] Beyrer C, Wirtz A, Walker D, Johns B, Sifakis F, Baral S (2011). The global HIV epidemics among men who have sex with men.

[8] Altman D, Aggleton P, Williams M, Kong T, Reddy V, Harrad D (2012). Men who have sex with men: stigma and discrimination. Lancet.

[9] amfAR, Johns Hopkins Center for Public Health and Human Rights (2012). Achieving an AIDS-free generation for gay men and other MSM: financing and implementation of HIV programs targeting MSM. http://www.amfar.org/uploadedFiles/_amfar.org/In_The_Community/Publications/MSM-GlobalRept2012.pdf.

[10] Malawi National Government (2012). 2012 Global AIDS response progress report: Malawi country report for 2010 and 2011.

[11] Baral S, Phaswana-Mafuya N (2012). Rewriting the narrative of the epidemiology of HIV in sub-Saharan Africa. SAHARA J.

[12] Penal Code Cap 7:01 Laws of Malawi, Sections 153, 156.

[13] Smith AD, Tapsoba P, Peshu N, Sanders EJ, Jaffe HW (2009). Men who have sex with men and HIV/AIDS in sub-Saharan Africa. Lancet.

[14] Baral S, Trapence G, Motimedi F, Umar E, Iipinge S, Dausab F (2009). HIV prevalence, risks for HIV infection, and human rights among men who have sex with men (MSM) in Malawi, Namibia, and Botswana. PLoS One.

[15] Fay H, Baral S, Trapence G, Motimedi F, Umar E, Iipinge S (2011). Stigma, health care access, and HIV knowledge among men who have sex with men in Malawi, Namibia, and Botswana. AIDS Behav.

[16] Ntata PR, Muula AS, Siziya S (2008). Socio-demographic characteristics and sexual health related attitudes and practices of men having sex with men in central and southern Malawi. Tanzan J Health Res.

[17] Sullivan PS, Carballo-Diéguez A, Coates T, Goodreau SM, McGowan I, Sanders EJ (2012). Successes and challenges of HIV prevention in men who have sex with men. Lancet.

[18] World Health Organization (2009). Prevention and treatment of HIV and other sexually transmitted infections among men who have sex with men and transgender populations: report of a technical consultation.

[19] Magnani R, Sabin K, Saidel T, Heckathorn D (2005). Review of sampling hard-to-reach and hidden populations for HIV surveillance. Aids.

[20] Malekinejad M, Johnston LG, Kendall C, Kerr LR, Rifkin MR, Rutherford GW (2008). Using respondent-driven sampling methodology for HIV biological and behavioral surveillance in international settings: a systematic review. AIDS Behav.

[21] Borgatti S (2002). NetDraw software for network visualization.

[22] Weller S, Davis K (2002). Condom effectiveness in reducing heterosexual HIV transmission. Cochrane Database Syst Rev.

[23] Salganik MJ (2006). Variance estimation, design effects, and sample size calculations for respondent-driven sampling. J Urban Health.

[24] Wirtz A, Trapence G, Jumbe V, Kamba D, Baral S (2012). “We are doing these things in an underground way”: structural barriers to HIV prevention and service utilization among men who have sex with men (MSM) in Malawi (Abstract #TUPE580).

[25] Ministry of Health (2008). Prevention of mother to child transmission of HIV and paediatric HIV care guidelines.

[26] Schonlau M, Liebau E (2012). Respondent-driven sampling. Stata J.

[27] StataCorp (2011). Stata statistical software: release 12.

[28] Kelesidis T, Landovitz RJ (2011). Preexposure prophylaxis for HIV prevention. Curr HIV/AIDS Rep.

[29] Smith D, Grant R, Weidle P, Lansky A, Mermin J, Fenton K (2011). Interim guidance: preexposure prophylaxis for the prevention of HIV infection in men who have sex with men. MMWR Morb Mortal Wkly Rep.

[30] Cohen MS, Chen YQ, McCauley M, Gamble T, Hosseinipour MC, Kumarasamy N (2011). Prevention of HIV-1 infection with early antiretroviral therapy. N Engl J Med.

[31] Marks G, Crepaz N, Senterfitt JW, Janssen RS (2005). Meta-analysis of high-risk sexual behavior in persons aware and unaware they are infected with HIV in the United States: implications for HIV prevention programs. J Acquir Immune Defic Syndr.

[32] Center for Disease Control and Prevention (2011). Sexually transmitted diseases treatment guidelines, 2010: special populations (MSM).

[33] Doyle AM, Mavedzenge SN, Plummer ML, Ross DA (2012). The sexual behaviour of adolescents in sub-Saharan Africa: patterns and trends from national surveys. Trop Med Int Health.

[34] Stockl H, Kalra N, Jacobi J, Watts C (2013). Is early sexual debut a risk factor for HIV infection among women in sub-Saharan Africa? A systematic review. Am J Reprod Immunol.

[35] Kelley CF, Rosenberg ES, O'Hara BM, Frew PM, Sanchez T, Peterson JL (2012). Measuring population transmission risk for HIV: an alternative metric of exposure risk in men who have sex with men (MSM) in the US. PLoS One.

[36] McKinnon LR, Kaul R (2012). Quality and quantity: mucosal CD4+ T cells and HIV susceptibility. Curr Opin HIV AIDS.

[37] Millett GA, Peterson JL, Flores SA, Hart TA, Jeffries WL, Wilson PA (2012). Comparisons of disparities and risks of HIV infection in black and other men who have sex with men in Canada, UK, and USA: a meta-analysis. Lancet.

[38] Baral S, Burrell E, Scheibe A, Brown B, Beyrer C, Bekker LG (2011). HIV risk and associations of HIV infection among men who have sex with men in peri-urban Cape Town, South Africa. BMC Public Health.

[39] Lane T, Raymond HF, Dladla S, Rasethe J, Struthers H, McFarland W (2011). High HIV prevalence among men who have sex with men in Soweto, South Africa: results from the Soweto Men's Study. AIDS Behav.

[40] Hladik W, Barker J, Ssenkusu JM, Opio A, Tappero JW, Hakim A (2012). HIV infection among men who have sex with men in Kampala, Uganda – a respondent driven sampling survey. PLoS One.

[41] Bohl DD, Raymond HF, Arnold M, McFarland W (2009). Concurrent sexual partnerships and racial disparities in HIV infection among men who have sex with men. Sex Transm Infect.

[42] Mah T, Halperin D (2010). Concurrent sexual partnerships and the HIV epidemics in Africa: evidence to move forward. AIDS Behav.

[43] Lorenc T, Marrero-Guillamon I, Llewellyn A, Aggleton P, Cooper C, Lehmann A (2011). HIV testing among men who have sex with men (MSM): systematic review of qualitative evidence. Health Educ Res.

[44] Parry C, Petersen P, Dewing S, Carney T, Needle R, Kroeger K (2008). Rapid assessment of drug-related HIV risk among men who have sex with men in three South African cities. Drug and Alcohol Depend.

[45] Rispel LC, Metcalf CA, Cloete A, Moorman J, Reddy V (2011). You become afraid to tell them that you are gay: health service utilization by men who have sex with men in South African cities. J Public Health Policy.

[46] Lane T, Mogale T, Struthers H, McIntyre J, Kegeles SM (2008). “They see you as a different thing”: the experiences of men who have sex with men with healthcare workers in South African township communities. Sex Transm Infect.

[47] Wirtz A, Walker D, Bollinger L, Sifakis F, Baral S, Johns B (2013). Modelling the impact of HIV prevention and treatment for men who have sex with men on HIV epidemic trajectories in low-and middle-income countries. Int J STD AIDS.

[48] Gray RT, Prestage GP, Down I, Ghaus MH, Hoare A, Bradley J (2013). Increased HIV testing will modestly reduce HIV incidence among gay men in NSW and would be acceptable if HIV testing becomes convenient. PLoS One.

[49] Larmarange J, Wade AS, Diop AK, Diop O, Gueye K, Marra A (2010). Men who have sex with men (MSM) and factors associated with not using a condom at last sexual intercourse with a man and with a woman in Senegal. PLoS One.

[50] Wade AS, Larmarange J, Diop AK, Diop O, Gueye K, Marra A (2010). Reduction in risk-taking behaviors among MSM in Senegal between 2004 and 2007 and prevalence of HIV and other STIs. ELIHoS Project, ANRS 12139. AIDS Care.

[51] Higa D, Crepaz N, Marshall K, Kay L, Vosburgh HW, Spikes P (2013). A systematic review to identify challenges of demonstrating efficacy of HIV behavioral interventions for gay, bisexual, and other men who have sex with men (MSM). AIDS Behav.

[52] Lorenc T, Marrero-Guillamon I, Aggleton P, Cooper C, Llewellyn A, Lehmann A (2011). Promoting the uptake of HIV testing among men who have sex with men: systematic review of effectiveness and cost-effectiveness. Sex Transm Infect.

[53] Mayer KH, Bekker L-G, Stall R, Grulich AE, Colfax G, Lama JR (2012). Comprehensive clinical care for men who have sex with men: an integrated approach. Lancet.

[54] Gelman A (2007). Struggles with survey weighting and regression modeling. Stat Sci.

[55] Gardner EM, McLees MP, Steiner JF, Del Rio C, Burman WJ (2011). The spectrum of engagement in HIV care and its relevance to test-and-treat strategies for prevention of HIV infection. Clin Infect Dis.

# Sub article: Men who have sex with men sensitivity training reduces homoprejudice and increases knowledge among Kenyan healthcare providers in coastal Kenya

**DOI:** 10.7448/IAS.16.4.18748

**Published:** 2013-12-02

**Authors:** Elise M van der Elst, Adrian D Smith, Evanson Gichuru, Elizabeth Wahome, Helgar Musyoki, Nicolas Muraguri, Greg Fegan, Zoe Duby, Linda-Gail Bekker, Bonnie Bender, Susan M Graham, Don Operario, Eduard J Sanders

**Affiliations:** 1 Kenya Medical Research Institute-Wellcome Trust Research Programme, Kilifi, Kenya; 2 Department of Public Health, University of Oxford, Oxford, UK; 3 National AIDS and STI Control Programme, Nairobi, Kenya; 4 Desmond Tutu HIV Foundation, Cape Town, South Africa; 5 International AIDS Vaccine Initiative, Nairobi, Kenya; 6 Department of Global Health, University of Washington, Seattle, WA, USA; 7 Department of Community Health, Brown University, Providence, RI, USA

**Keywords:** sensitivity training, MSM behaviour, Homophobia Scale, homoprejudice, healthcare workers, Kenya

## Abstract

**Introduction:**

Healthcare workers (HCWs) in Africa typically receive little or no training in the healthcare needs of men who have sex with men (MSM), limiting the effectiveness and reach of population-based HIV control measures among this group. We assessed the effect of a web-based, self-directed sensitivity training on MSM for HCWs (www.marps-africa.org), combined with facilitated group discussions on knowledge and homophobic attitudes among HCWs in four districts of coastal Kenya.

**Methods:**

We trained four district “AIDS coordinators” to provide a two-day training to local HCWs working at antiretroviral therapy-providing facilities in coastal Kenya. Self-directed learning supported by group discussions focused on MSM sexual risk practices, HIV prevention and healthcare needs. Knowledge was assessed prior to training, immediately after training and three months after training. The Homophobia Scale assessed homophobic attitudes and was measured before and three months after training.

**Results:**

Seventy-four HCWs (68% female; 74% clinical officers or nurses; 84% working in government facilities) from 49 health facilities were trained, of whom 71 (96%) completed all measures. At baseline, few HCWs reported any prior training on MSM anal sexual practices, and most HCWs had limited knowledge of MSM sexual health needs. Homophobic attitudes were most pronounced among HCWs who were male, under 30 years of age, and working in clinical roles or government facilities. Three months after training, more HCWs had adequate knowledge compared to baseline (49% vs. 13%, McNemar's test *p*<0.001); this was most pronounced in those with clinical or administrative roles and in those from governmental health providers. Compared to baseline, homophobic attitudes had decreased significantly three months after training, particularly among HCWs with high homophobia scores at baseline, and there was some evidence of correlation between improvements in knowledge and reduction in homophobic sentiment.

**Conclusions:**

Scaling up MSM sensitivity training for African HCWs is likely to be a timely, effective and practical means to improve relevant sexual health knowledge and reduce personal homophobic sentiment among HCWs involved in HIV prevention, testing and care in sub-Saharan Africa.

## Introduction

Sub-Saharan Africa has a very high burden of HIV-1 infection, of which a substantial proportion occurs among populations reporting high-risk sexual behaviour such as transactional sex and anal intercourse [1]. Such populations suffer from stigma and rejection, and they have been neglected by many HIV prevention and care programmes [2]. As a result, most African healthcare workers (HCWs) have not been informed about the risk of HIV transmission with regard to heterosexual or homosexual anal sex. In addition, African HCWs may lack understanding of the many challenges that men who have sex with men (MSM) and other key populations face in healthcare facilities [3].

Societal discrimination on the grounds of sexual orientation has been reported frequently among African MSM, taking the form of sexual, physical and verbal assault [4–7], and a number of studies have demonstrated an association between reported experience of discrimination and HIV risk or risk behaviour [8]. Similarly, high levels of internalized homophobia among MSM have been reported in Nigeria [8], South Africa [9,10] and Uganda [11], known to be associated with individual HIV risk-taking behaviour [12]. Overt stigmatization specifically from HCWs in the context of HIV testing and care, such as denial of service [4,3,13] and harassment in clinic spaces [14], has been reported as a key element of perceived discrimination, presenting a deterrent to service access [15] or accurate disclosure of behavioural risk [14]. In the absence of resources targeted to groups at high risk of HIV infection, the marginalization of MSM from public HIV prevention and treatment resources can only hamper the effectiveness of national HIV control efforts [6].

Health worker training, social mobilization and community engagement were prioritized as structural interventions in a recent consultation on priority areas for MSM HIV prevention research involving 69 participants from 17 African countries [16]. HCWs have also been *called to action* to reduce stigma and discrimination, provide integrated services for mental health concerns and substance use, screen MSM routinely for HIV and sexually transmitted infections (STIs) and ensure training for all personnel in clinical settings [3]. As yet, African HCWs lack any evidence-based, culturally adapted training model that is sensitive to MSM needs. This problem likely stems from cultural taboos about anal sex practices, even in opposite-sex couples [17], and strong political, religious and public prejudice against same-sex practices [18].

Since 2005, biomedical research has been ongoing with both HIV-1 negative MSM and MSM living with HIV in coastal Kenya [1,19]. To date, the only incidence data for African MSM derive from our cohort and a related cohort in Nairobi [19,20]. Overall HIV-1 incidence among young MSM in coastal Kenya was as high as 8.6 (95% confidence interval [CI]: 6.7–11.0) per 100 person-years of observation [19]. The majority of these MSM reported sex work, and large numbers of such men have been identified in coastal Kenya [21]. Similarly, our cohort study of MSM living with HIV showed that 40% had less than 95% antiretroviral therapy (ART) adherence, compared to 29% of heterosexual men and 12% of women who were followed in the same research setting [1]. These findings prompted us to brief health authorities and develop materials to help improve care for MSM in Kenya and elsewhere in Africa.

Internet-based learning (e-learning) as a cognitive tool has increasingly been used in health professions in resource-constrained low- and middle-income countries [22]. E-learning technologies offer learners control over content, learning sequence, pace of learning, time and often media, allowing learners to tailor their experiences to meet their personal learning objectives [23]. The internet-based HCW MSM sensitivity training described here represents our attempt to deploy meaningful, clinically relevant material to meet this specific learning need within Kenyan HIV services through adaptation of an existing training curriculum to a web environment.

“MSM: An introductory guide for health workers in Africa” is a paper-based HCW sensitization training first developed in 2010. The content of training was validated and revised through a programme of extensive classroom use in South Africa, and following expert review [24]. The paper-based training guide was electronically converted to a self-directed electronic format and published online in July 2011, a version of which was adapted for use in this study.

The objectives of this study were (1) to assess the feasibility of facilitated self-directed learning of MSM health issues in coastal Kenya and (2) to evaluate the effect of the training intervention upon HCW knowledge and attitudes.

## Methods

### Study site and participants

Seventy-four HCWs involved in HIV prevention, treatment and care services in coastal Kenya were recruited to participate in the study. We mapped 54 ART-providing governmental and nongovernmental health providers in four districts in coastal Kenya (Kilindini, Mombasa, Kilifi and Malindi). An average of two staff representatives from each health-providing facility were invited to the training intervention, including clinicians and counsellors as well as clinic administrators.

Four “district AIDS/STD coordinators” (DASCOs) working within the study districts were trained to lead the MSM sensitization training during a 2-day “training-of-trainers” course similar to the training proper. An additional day was used to prepare focus-group topic guides and organizational matters. The study procedures were approved by the ethical review board at the Kenya Medical Research Institute, and all participants provided written informed consent for impact evaluation. HCWs received Ksh2000 (approximately US$24.00) to cover travel expenses and lodging.

### The training intervention

The training consisted of two consecutive days and included eight modules which were taken in four sessions (i.e., two computer modules per session). Each session was followed by a group discussion. Each group size comprised 18–19 participants. DASCOs were supported by four members of the research team (i.e., a community liaison officer, a research counsellor, an MSM staff-fieldworker and a social scientist) and two members of a local LGBTI (lesbian, gay, bisexual, transgender and intersex) organization. HCWs were introduced to the sensitivity training on MSM health issues and learned that the training consisted of computer-assisted learning (http://www.marps-africa.org) and group discussions. The curriculum consisted of the following modules of study: (1) *MSM and HIV in sub-Saharan Africa*; (2) *Stigma*; (3) *Identity, coming out and disclosure*; (4) *Anal sex and common sexual practices*; (5) *HIV and sexually transmitted infections*; (6) *Mental health, anxiety, depression and substance abuse*; (7) *Condom and lubricant use*; and (8) *Risk reduction counselling*. Modules were designed to be self-completed in 1–2 hours each, including multiple-choice questions (median 12, range 9–16) at the end of each module. A score of 71% correct was required to advance to the next module, and upon successful completion of all eight modules, participants were sent a link to download their course certificate. A post-course evaluation asked for opinions and suggestions for course improvements, using both closed and open-ended questions.

Discussion topics included the identification of subcategories of MSM and their characteristics, sexual practices of MSM and risks for HIV and STI transmission, factors that make MSM vulnerable to STIs and HIV, risk assessment in counselling MSM, best practice for sexual history taking and sexual health examination with MSM, relevant information on safer sex for MSM, personal values and attitudes towards MSM, and addressed stigma and strategies to improve communication with clients who are MSM. At the end of the training, HCWs discussed a work plan on how to strengthen clinical care and uptake of HIV and STD testing for MSM in their day-to-day practice. Study participants with a clinical role were also requested to keep a journal for three months to document and reflect upon their work practices and personal attitudes towards MSM.

### Data collection

Course participants completed an online registration, including socio-demographic characteristics (age, gender and level of education and training), details of working practice (role within, type and location of healthcare organization) and specific experience working with HIV prevention, treatment and care with the most at-risk populations (MARPs) in Africa. To assess baseline levels of knowledge, participants conducted a pre-course 24-item multiple-choice assessment covering key learning outcomes across the course material, and they completed a 25-item Homophobia Scale (HS; adapted from Wright et al. [25]). The same two measures were repeated three months after course completion to assess sustained changes in knowledge of and attitudes towards MSM. Immediate post-course knowledge was assessed using the same pre-knowledge questionnaire upon completion of the eight modules. The results of pre-training and post-training assessments were not communicated to participating HCWs.

### Measurement scales

Knowledge scores of course material were divided into the following categories: Poor (<17 questions correctly answered),Good (17–22 questions correctly answered) and Excellent (>22 questions correctly answered). When 17 or more questions were correctly answered, the immediate post-training knowledge was considered adequate.

The HS, which was developed and standardized among college students in the United States by Wright et al. [25], was adapted for use in Kenya. The HS aims to measure thoughts, feelings and behaviours towards homosexuality and MSM, and it consists of 25 statements to which respondents indicate their level of agreement on a 5-point Likert scale (Table 1). Questions were reviewed and adapted by three Kenyan research staff and HCWs with professional and personal experience working with local MSM. The adapted HS is shown in Table 1 and reflects changes in terminology (e.g., “gay” was replaced with “MSM” and “faggot” with “shoga” in question 9) to reflect local terminology in current use. *I have damaged property of gay persons, such as “keying” their cars*, was replaced with *Homosexuality should be treated as an illness/Homosexuality can be cured* (question 17); *I would feel comfortable with having a gay roommate* was replaced with *Homosexuality is un-African/is something brought by foreigners* (question 18); and *I have rocky relationships with people that I suspect are gay* was replaced with *Gay men have the same rights to public/tax-funded services as straight men* (question 25). Responses to items 1, 2, 4, 5, 6, 9, 12, 13, 14, 15, 17, 18, 19, 21, 23 and 24 were reverse coded (item scores 1=5, 2=4, 3=3 etc.). The total HS score (HSS) was the sum of all item scores, with 25 subtracted from the total. The range is between 0 and 100, with an HSS of 0 being the least homophobic and 100 being the most homophobic.

**Table 1:** Homophobia Scale and MSM sensitivity training for healthcare workers (HCWs), coastal Kenya, 2011–2012

***This questionnaire is designed to measure your thoughts, feelings and behaviours with regards to homosexuality. It is not a test, so there are no right or wrong answers. Answer each item by circling the number after each question**.*						
**1=Strongly agree**						
**2=Agree**						
**3=Neither agree nor disagree**						
**4=Disagree**						
**5=Strongly disagree**						

1	MSM make me nervous	1	2	3	4	5
2	MSM deserve what they get	1	2	3	4	5
3	Homosexuality is acceptable to me	1	2	3	4	5
4	If I discovered a friend was an MSM, I would end the friendship	1	2	3	4	5
5	I think homosexual people should not work with children	1	2	3	4	5
6	I make derogatory remarks about MSM people	1	2	3	4	5
7	I enjoy the company of MSM	1	2	3	4	5
8	Marriage between homosexual individuals is acceptable	1	2	3	4	5
9	I make derogatory remarks like “shoga” or “queer” to people who I suspect are MSM	1	2	3	4	5
10	It does not matter to me whether my friends are MSM or straight	1	2	3	4	5
11	It would upset me if I learned that a close friend was homosexual	1	2	3	4	5
12	Homosexuality is immoral	1	2	3	4	5
13	I tease and make jokes about MSM	1	2	3	4	5
14	I feel that you cannot trust a person who is homosexual	1	2	3	4	5
15	I fear homosexual persons will make sexual advances towards me	1	2	3	4	5
16	Organizations which promote gay rights are not necessary	1	2	3	4	5
17	Homosexuality should be treated as an illness/Homosexuality can be cured	1	2	3	4	5
18	Homosexuality is un-African/is something brought by foreigners	1	2	3	4	5
19	I would hit a homosexual for coming on to me	1	2	3	4	5
20	Homosexual behaviour should not be against the law	1	2	3	4	5
21	I avoid MSM individuals	1	2	3	4	5
22	It bothers me to see two homosexual people together in public	1	2	3	4	5
23	When I see an MSM, I think, “What a waste.”	1	2	3	4	5
24	When I meet someone, I try to find out if he or she is MSM	1	2	3	4	5
25	Gay men have the same rights to public/tax-funded services as straight men	1	2	3	4	5

### Data analysis

Analysis was conducted using Stata 11.0 (StataCorp LP, College Station, TX, USA). Binary and categorical characteristics of study participants, established at baseline, were compared using chi-square tests. Although both knowledge and HS scores before and after training approximated to Gaussian distributions, differences between paired measures were non-normal, and thus unadjusted nonparametric methods were used for analysis. Median differences between pre- and post-training knowledge and homophobia score are reported with an interquartile range (IQR). A Wilcoxon signed rank test for matched pairs was applied to test the statistical significance of differences between pre- and post-training scores. Mann–Whitney and exact McNemar's tests were used to test differences in scores and binary measures, respectively, by HCW characteristics. Spearman's rank was used to assess correlation between pre- and post-training scores, and knowledge and HS scores at both points. Multivariate linear regression models of pre- and post-training score outcomes were explored, but they yielded no additional insight beyond bivariate analysis.

## Results

Seventy-four HCWs were recruited to participate in the training programme, and their characteristics are shown in Table 2. The majority were female, and the mean age of participants was 32 years (range: 23 to 53). Sixty-two participants (84%) worked at a government health facility (hospital or clinic), seven (9%) worked at a local nongovernmental organization (NGO) and three (4%) represented faith-based organizations. Most (74%) were in a clinical role (nurse or clinical officer). Irrespective of job role, 8% had received any previous training on how to counsel MSM clients, and a similarly low proportion (7%) had ever received training on how to counsel on anal sex practices. HCWs who had received training on anal sex practices were more likely to have ever asked their male patients if they had sex with men than HCWs who did not report previous training (86% (6/7) versus 31% (21/67), χ^2^ *p*<0.01).

**Table 2:** Characteristics of 74 healthcare workers (HCWs)

Registration characteristics	HCWs selected for two-day online training *N*=74 *N* (%)
Gender	
Men	24 (32)
Female	50 (68)
Age (years)	
18–29	3 (4)
30–39	44 (59)
40 +	27 (36)
Organization type	
Government	62 (84)
NGO	7 (9)
Other	5 (7)
Education level	
Up to secondary	6 (8)
Tertiary	68 (92)
Job type	
Clinical	52 (70)
Counselling	12 (16)
Administrative	7 (9)
Other	3 (4)
**Prior MSM training experience**	
Counselling clients who are MSM	
No	68 (92)
Yes	6 (8)
Counselling on anal sex practices	
No	67 (91)
Yes	7 (9)
Ever asked male clients about sexual acts with other men	
No	47 (64)
Yes	27 (36)

### Training logistics

Most HCWs (73% (54/74)) required 1 hour or less to complete each module, whilst 11% (8/74) required 2 hours or more per module. While 68% (50/74) considered the course duration to be “just right,” 23% (17/74) said that it was too short, and 9% (7/74) that it was too long. Overall, by the end of training, 60/74 (81%) HCWs reported feeling empowered to discuss MSM behaviour and anal sex in their professional work.

All participants said they would recommend the course to others. Open-ended suggestions for course improvements are presented in Table 3. Study participants recommended that the training should be taken by all health stakeholders dealing with MSM issues and be included in medical training. There was an interest in similar training related to other key populations (e.g., women who have sex with women and sex workers).

**Table 3:** End-of-course suggestions for course improvements

**Study participants (SP) (** ***N*** **=74)**
Selected from 45 (61%) responses
**Theme: Expand training to all healthcare workers (HCWs) and other institutions**
“This online training should be streamed or provided to all institutions and politicians to sensitize them on the need to recognize MSM.”
“Should be introduced in medical training departments.”
“It should be done by all health stake holders dealing with MSM issues.”
**Theme: Advertisement and promotion website**
“Reach more people through media eg Radio and schools.”
“Make it available at facility by providing computer.”
**Theme: Connectivity, internet, computers and mobile phones**
“Improve internet speed.”
“No suggestion, it is that I am still learning how to use a computer.”
**Theme: Duration of course**
“It should also include field work.”
“The course is very interesting, it should be for at least five days, so we get time to discuss more.”
(Nineteen additional participants asked for a longer course.)
**Theme: Improve specific modules and further learning**
“Add women who have sex with women/prostitution.”
“Add more pictures on the STI module. Make the questions more relevant for some are contradictory. Have authoritative literature references.”
“The picture of the actual penetration on page 68 [cartoon of man penetrating a man while using a condom] is somehow too much to view. Better picture can be used to demonstrate the same.”
“We need more testimony clips to narrate how many MSM got to fight stigma and be where they are now.”
“More research should be conducted on whether homosexuality could be reversed.”
“Perhaps translation in Kiswahili.”

### Effect of training on MSM sexual health knowledge among healthcare workers

Table 4 shows knowledge of MSM sexual health issues among participants before the training course, and upon reassessment three months after the course. Prior to the training course, only 10/74 (14%) had an “adequate” level of knowledge of MSM issues (threshold score: 17/24), reflecting a median score of 54% (IQR 49–63%). Levels of knowledge were similar by socio-demographic and workplace characteristics of HCWs, although it was somewhat lower for HCWs in administrative roles compared to other roles (median 42 vs. 54, Mann–Whitney *p*=0.293).

**Table 4:** Change in MSM sexual health knowledge from baseline to three months post-training

					Difference between pre-training and post-training multiple-choice questions %†		
					
		Healthcare workers (HCWs) *N*	Pre-training (baseline) Median (%)	Post-training (three month) Median (%)	Median difference (%)	Interquartile range	*P*-value (Wilcoxon)
All		74	54	67	+12	4 to 21	<0.001
Gender	Male	24	56	71	+16	5 to 21	<0.001
	Female	50	54	65	+11	1 to 21	<0.001
	Under 30	23	54	71	+16	0 to 21	0.001
Age group	30 to 39	34	58	63	+9	0 to 21	0.002
	40 or over	17	54	71	+12	4 to 21	0.002
	Clinical	52	54	71	+13	8 to 21	<0.001
	Counselling	12	58	56	0	−9 to 13	0.813
Job type	Admin	7	42	75	+13	4 to 37	0.022
	Other	3	54	73	+19	9 to 29	0.190
	Government	62	54	71	+13	5 to 21	<0.001
Facility	NGO	7	58	67	+12	−8 to 17	0.267
	Other	5	54	58	+4	−5 to 25	0.414

† Limited to 71 HCWs with paired observations.

At the end of training, 70/74 (95%) HCWs had adequate course knowledge (exact McNemar's χ^2^ *p*<0.001 vs. pre-training). At three months after the course, 35 (49%) of the 71 HCWs reassessed had retained “adequate” knowledge compared to 9/71 (13%) at pre-training (exact McNemar's χ^2^ *p*<0.001). This represented a significant increase in the median assessment score of 12% (IQR 4–21%) between baseline and three-month knowledge assessments (Wilcoxon signed test for matched pairs *p*<0.001). Significant sustained improvements in knowledge were apparent for all HCW age groups and genders, those with clinical or administrative roles and those from governmental health providers.

Pre-training and three-month post-training scores were negatively correlated (Spearman's rho −0.51, *p<* 0.001), indicating that improvements in knowledge tended to be highest among HCWs with lower pre-training knowledge. There were no significant differences in the degree of knowledge gain by the gender or age group of HCWs; however, participants in counselling roles achieved significantly lower gains in sustained knowledge than other HCWs (median difference: 0% vs. +13%, Mann–Whitney *p*=0.0163).

### Effect of training on personal attitudes toward MSM among healthcare workers

Table 5 shows HS scores among HCWs prior to training and at reassessment three months later. Overall, the median HS score prior to training was 68/100, representing extensive agreement with homophobic statements and disagreement with statements indicating tolerance of MSM (see Table 5). Male HCWs had slightly higher HS scores at baseline than female HCWs, while HS scores declined with increasing age group but differences were not statistically significant. HCWs in clinical roles (medical and nursing) had higher HS scores than other staff (median 71 vs. 66 respectively, Mann–Whitney *p*=0.116), and HCWs working in government facilities had significantly higher HS scores than HCWs in NGOs (Table 5, Mann–Whitney *p*=0.037).

**Table 5:** Change in Homophobia Score (HS) from baseline to three months post-training

					Difference between pre-training and post-training HS†		
					
		Healthcare workers (HCWs) *N*	Pre-training (baseline) Median HS (0–100)	Post-training (three month) Median HS score (0–100)	Median difference (score)	Interquartile range	*P*-value (Wilcoxon)
All		74	68	60	−8	−2 to −15	<0.001
Gender	Male	24	70	60	−9	−4 to −21	0.001
	Female	50	68	60	−7	−1 to −13	<0.001
	Under 30	23	71	62	−9	−3 to −14	<0.001
Age group	30 to 39	34	68	59	−6	−1 to −13	<0.001
	40 or over	17	67	61	−10	−4 to −24	0.001
	Clinical	52	71	61	−10	−3 to −20	<0.001
Job type	Counselling	12	64	60	−5	+1 to −9	0.031
	Administrative	7	68	55	−11	−6 to −19	0.022
	Other	3	66	61	−2	0 to −4	0.317
	Government	62	71	60	−9	−2 to −15	<0.001
Facility	Nongovernmental organization	7	62	56	0	+1 to −13	0.444
	Other	5	78	60	−9	−5 to −21	0.043

† Limited to 71 HCWs with paired observations.

The majority of HCWs reported lower HS scores three months post-training (80.3%, 57/71) compared to their baseline HS score; in four (5.6%), HS scores were unchanged; and in 14.1% (10/71), HS scores were higher after training than before. Overall, the median decrease in individual HS score after training was 8 points (IQR 2–15), which was statistically significant. These findings did not change in a sensitivity analysis omitting the three HS questions that were culturally adapted (data not shown). Individual pre-training and post-training HS scores were negatively correlated (Spearman's rho=− 0.71, *p*<0.001), reflecting the tendency for HCWs with high pre-training HS scores to exhibit greater decreases in this measure as a result of training (Figure 1).

**Figure 1:** Baseline HS score and difference three months after training (71 participants).

Male HCWs and those working in clinical roles and in governmental institutions recorded the most pronounced reductions in HS score subsequent to training, although differences in median reduction comparing HCWs’ gender, age group, staff role and institution were not statistically significant. More modest declines in HS score were apparent for counsellors (median reduction after training: 4 points) and staff of NGOs (median reduction after training: 0 points); however, it is notable that these groups reported relatively low HS scores prior to training. Collectively, there was some evidence for correlation between scale of increase in individual knowledge and scale of decline in HS score, and this was of borderline statistical significance (Spearman's rho=−.21, *p*=0.087).

## Discussion

This formal evaluation of a training course aimed specifically to improve knowledge and awareness of MSM sexual health needs among healthcare staff involved in frontline HIV prevention, treatment and care to adult populations in sub-Saharan Africa. Specific and accurate knowledge relevant to the management of behavioural and clinical risks for MSM clients prior to training was poor.

Whilst this may not be surprising in the face of long-standing neglect of Kenyan MSM within HIV policy and resource allocation and a lack of attention to MSM within medical, nursing and HIV counselling training in Kenya, it draws focus to the challenge of maintaining and extending the professional competence of the existing HIV workforce to match the epidemiological realities of the Kenyan HIV epidemic – especially since the National AIDS & Sexually Transmitted Diseases Control Programme (NASCOP) requires Kenyan HCWs to document the number and category of MSM using HIV services.

Whilst targeted services may well be necessary for subpopulations of MSM, such as male sex workers, they are unlikely to replace the need for MSM-specific clinical care among general health services. MSM-specific programmes have aroused considerable social antipathy in Kenya to date [26] and may in any case not be perceived as accessible to men who covertly engage in homosexual behaviour [27]. Furthermore, surveillance of key populations, including MSM, and strategic information on service coverage to these groups are now an international requirement [28].

The combination of self-directed, modular computer-based learning supplemented by group discussions facilitated by trainers identified from within the existing workforce may offer a relatively sustainable and mobile model for episodic health professional training in this context. The learning content of this course is freely available as a web resource, yet reliable access to internet services remains elusive and expensive in most parts of the country. Even where it is available, the narrative reflections by participants who undertook this training emphasize the importance of the sanction provided by facilitated group discussions to share and explore personal and professional issues arising from the training content that may well be lost in self-directed learning [29].

The brief training programme described here resulted in significant improvements in knowledge of MSM sexual health issues pertinent to day-to-day prevention and clinical practice, and it was sustained by most trainees until at least three months after training. Increase in knowledge was accompanied by a reduction in negative attitudes toward MSM over the same period. Encouragingly, the positive effect of training upon knowledge and personal attitudes toward MSM was strongest among HCWs who had poor levels of knowledge and/or more extreme negative attitudes toward MSM prior to training. That positive changes were most marked among HCWs in clinical roles within governmental settings, which represent the backbone of Kenyan HIV services, is cause for particular optimism. Studies to date of perceived barriers to healthcare access identified by MSM in Kenya [7,30] and elsewhere in sub-Saharan Africa [31,32] have reported denial of service, lack of confidentiality, ignorance and verbal abuse from governmental HIV services as central challenges in accessing sexual and general health services. The finding of this study, albeit preliminary, suggests both that members of this workforce are willing to learn about MSM sexual health and that their knowledge and attitude toward MSM are responsive to this learning.

This study has a number of limitations. The HS, which was originally developed and validated among college students in the United States [25], required amendment to preserve its face validity in a markedly different research context. Whereas the modified scale was responsive to change with training, and these changes were robust to sensitivity analysis excluding modified scale items, the objective meaning of absolute scores and the convergent and divergent validity of this scale in this population remain to be established. Furthermore, although the assessment of training effects upon knowledge and homophobic sentiment was assessed at an endpoint long after the training itself, the longer term effect of training cannot be assumed from this study. Finally, this study lacked a control group which, ideally, would have consisted of HCWs not receiving the intervention and HCWs only participating in the self-directed learning.

We report qualitative narratives among HCWs returning to their workplace after training but finding little support for new perspectives amongst (untrained) colleagues [29]. In a recent qualitative assessment of counselling challenges regarding MSM that are experienced by Kenyan counsellors and clinicians in coastal Kenya, all felt that lack of training and supervisory support impacted their ability to serve MSM [33]. These findings may suggest that either longer term support of trained HCWs and/or more extensive facility-based training of all staff may be prerequisites to longer term changes in institutional practice. While knowledge of same-sex practices is a first step to improve services to MSM, serving MSM in day-to-day practice will further improve services. Follow-up of health workers trained in this study is planned and may provide insights in current care services provided to MSM at two years post-training. Additionally, although the training itself was conducted by facilitators from governmental services that were specially trained for the role, the study was run by a team who was unusually experienced in working with Kenyan MSM, which may threaten generalizability to other settings.

Finally, the ultimate goals of improving knowledge of MSM sexual health needs and reducing prejudicial attitudes toward MSM in healthcare settings are to enhance the accessibility of population-based, public health services to MSM themselves. Although surely a prerequisite to accessible HIV prevention, treatment and care for MSM, the extent to which changes in the attitudes and practices of healthcare providers are reflected in the perceived and practical accessibility and acceptability of services to MSM themselves is unknown. Further study will be required to establish the effect of this brief intervention on long-term attitudes and professional practices towards MSM, and what practical contribution such strategies might make to addressing unmet HIV-related needs among MSM.

## Conclusions

In summary, we developed, implemented and evaluated a brief training intervention addressing knowledge and attitudes toward MSM and their sexual health needs in Kenya. The training, which combined self-directed and facilitated group learning, increased health worker knowledge and reduced homophobic attitudes up to three months after training. Scaling up such interventions offers a straightforward response to the immediate need to support HCWs in offering accessible and informed services to address the largely sexual health needs among MSM in Kenya.

## References

[1] Graham SM, Mugo P, Gichuru E, Thiong'o A, Macharia M, Okuku HS (2013). Adherence to antiretroviral therapy and clinical outcomes among young adults reporting high-risk sexual behavior, including men who have sex with men, in coastal Kenya. AIDS Behav.

[2] Smith AD, Tapsoba P, Peshu N, Sanders EJ, Jaffe HW (2009). Men who have sex with men and HIV/AIDS in sub-Saharan Africa. Lancet.

[3] Beyrer C, Sullivan PS, Sanchez J, Dowdy D, Altman D, Trapence G (2012). A call to action for comprehensive HIV services for men who have sex with men. Lancet.

[4] Geibel S, Luchters S, King'Ola N, Esu-Williams E, Rinyiru A, Tun W (2008). Factors associated with self-reported unprotected anal sex among male sex workers in Mombasa, Kenya. Sex Transm Dis.

[5] Baral S, Trapence G, Motimedi F, Umar E, Iipinge S, Dausab F (2009). HIV prevalence, risks for HIV infection, and human rights among men who have sex with men (MSM) in Malawi, Namibia, and Botswana. PLoS One.

[6] Kajubi P, Kamya MR, Raymond HF, Chen S, Rutherford GW, Mandel JS (2008). Gay and bisexual men in Kampala, Uganda. AIDS Behav.

[7] Onyango-Ouma W, Burungi H, Geibel S (2005). Understanding the HIV/STI risks and prevention needs of men who have sex with men in Nairobi, Kenya.

[8] Adebajo SB, Eluwa GI, Allman D, Myers T, Ahonsi BA (2012). Prevalence of internalized homophobia and HIV associated risks among men who have sex with men in Nigeria. Afr J Reprod Health.

[9] Vu L, Tun W, Sheehy M, Nel D (2012). Levels and correlates of internalized homophobia among men who have sex with men in Pretoria, South Africa. AIDS Behav.

[10] Knox J, Sandfort T, Yi H, Reddy V, Maimane S (2011). Social vulnerability and HIV testing among South African men who have sex with men. Int J STD AIDS.

[11] Ross MW, Kajubi P, Mandel JS, McFarland W, Raymond HF (2013). Internalized homonegativity/homophobia is associated with HIV-risk behaviours among Ugandan gay and bisexual men. Int J STD AIDS.

[12] Ross MW, Berg RC, Schmidt AJ, Hospers HJ, Breveglieri M, Furegato M (2013). Internalised homonegativity predicts HIV-associated risk behavior in European men who have sex with men in a 38-country cross-sectional study: some public health implications of homophobia. BMJ Open.

[13] Centre for the Right to Health (2008). Assessing the HIV/AIDS burden among men who have sex with men in Abuja, Nigeria.

[14] Lane T, Stuthers SH, Hamilton R, Gray G, McIntyre J (2004). HIV risk behaviours among black MSM in Johannesburg, South Africa. XV International Conference on AIDS.

[15] Rispel L, Metcalf MC, Cloete A, Reddy V (2011). You become afraid to tell them you are gay: availability and utilisation of health services by men who have sex with men (MSM) in the Johannesburg/Thekwini Men's Study (JEMS). Journal of Public Health Policy.

[16] Baral S, Scheibe A, Sullivan P, Trapence G, Lambert A, Bekker LG (2013). Assessing priorities for combination HIV prevention research for men who have sex with men (MSM) in Africa. AIDS Behav.

[17] Halperin DT (1999). Heterosexual anal intercourse: prevalence, cultural factors, and HIV infection and other health risks, Part I. AIDS Patient Care STDS.

[18] Semugoma P, Nemande S, Baral SD (2012). The irony of homophobia in Africa. Lancet.

[19] Sanders EJ, Okuku HS, Smith AD, Mwangome M, Wahome E, Fegan G (2013). High HIV-1 incidence, correlates of HIV-1 acquisition, and high viral loads following seroconversion among MSM. AIDS.

[20] Price MA, Rida W, Mwangome M, Mutua G, Middelkoop K, Roux S (2012). Identifying at-risk populations in Kenya and South Africa: HIV incidence in cohorts of men who report sex with men, sex workers, and youth. J Acquir Immune Defic Syndr.

[21] Geibel S, van der Elst EM, King'ola N, Luchters S, Davies A, Getambu EM (2007). “Are you on the market?”: a capture-recapture enumeration of men who sell sex to men in and around Mombasa, Kenya. AIDS.

[22] Frehywot S, Vovides Y, Talib Z, Mikhail N, Ross H, Wohltjen H (2013). E-learning in medical education in resource constrained low- and middle-income countries. Hum Resour Health.

[23] Ruiz JG, Mintzer MJ, Leipzig RM (2006). The impact of E-learning in medical education. Acad Med.

[24] Brown B, Duby Z, Schiebe A, Sanders E (2011). Men who have sex with men: an introducory guide for health care workers in Africa. Revised edition.

[25] Wright LE, Adams HE, Bernat JA (1999). Development and validation of the homophonbia scale. J Psychopathol Behav Assess.

[26] BBC News (2010). Gay pride and prejudice in Kenya [Internet]. http://www.bbc.co.uk/news/10320057.

[27] Smith AD (2013). Men who have sex with men in sub-Saharan Africa: a case study in Kenya.

[28] National AIDS Control Council, Office of the President, Kenya, 2008 UNGASS 2008 - Country Report for Kenya.

[29] van der Elst EM, Gichuru E, Omar A, Kanungi J, Duby Z, Midoun M (2013). Experiences of Kenyan health care workers providing services to men who have sex with men: qualitative findings from a sensitivity training program. J Int AIDS Soc.

[30] Sharma A, Bukusi E, Gorbach P, Cohen CR, Muga C, Kwena Z (2008). Sexual identity and risk of HIV/STI among men who have sex with men in Nairobi. Sex Transm Dis.

[31] Lane T, Mogale T, Struthers H, McIntyre J, Kegeles SM (2008). “They see you as a different thing”: the experiences of men who have sex with men with healthcare workers in South African township communities. Sex Transm Infect.

[32] Fay H, Baral SD, Trapence G, Motimedi F, Umar E, Iipinge S (2011). Stigma, health care access, and HIV knowledge among men who have sex with men in Malawi, Namibia, and Botswana. AIDS Behav.

[33] Taegtmeyer M, Davies A, Mwangome M, van der Elst EM, Graham SM, Price MA (2013). Challenges in providing counselling to MSM in highly stigmatized contexts: results of a qualitative study from Kenya. PLoS One.

# Sub article: “They are human beings, they are Swazi”: intersecting stigmas and the positive health, dignity and prevention needs of HIV-positive men who have sex with men in Swaziland

**DOI:** 10.7448/IAS.16.4.18749

**Published:** 2013-12-02

**Authors:** Caitlin E Kennedy, Stefan D Baral, Rebecca Fielding-Miller, Darrin Adams, Phumlile Dludlu, Bheki Sithole, Virginia A Fonner, Zandile Mnisi, Deanna Kerrigan

**Affiliations:** 1 Department of International Health, Johns Hopkins Bloomberg School of Public Health, Baltimore, MD, USA; 2 Department of Epidemiology, Johns Hopkins Bloomberg School of Public Health, Baltimore, MD, USA; 3 Department of Health Behavior, Emory University School of Public Health, Atlanta, GA, USA; 4 Futures Group, Washington, DC, USA; 5 Mbabane, Swaziland; 6 University of Stellenbosch, Stellenbosch, South Africa; 7 Ministry of Health and Social Welfare, Mbabane, Swaziland; 8 Department of Health, Behavior and Society, Johns Hopkins Bloomberg School of Public Health, Baltimore, MD, USA

**Keywords:** men who have sex with men, positive health dignity and prevention, people living with HIV, qualitative research, Swaziland

## Abstract

**Introduction:**

Despite the knowledge that men who have sex with men (MSM) are more likely to be infected with HIV across settings, there has been little investigation of the experiences of MSM who are living with HIV in sub-Saharan Africa. Using the framework of positive health, dignity and prevention, we explored the experiences and HIV prevention, care and treatment needs of MSM who are living with HIV in Swaziland.

**Methods:**

We conducted 40 in-depth interviews with 20 HIV-positive MSM, 16 interviews with key informants and three focus groups with MSM community members. Qualitative analysis was iterative and included debriefing sessions with a study staff, a stakeholders’ workshop and coding for key themes using Atlas.ti.

**Results:**

The predominant theme was the significant and multiple forms of stigma and discrimination faced by MSM living with HIV in this setting due to both their sexual identity and HIV status. Dual stigma led to selective disclosure or lack of disclosure of both identities, and consequently a lack of social support for care-seeking and medication adherence. Perceived and experienced stigma from healthcare settings, particularly around sexual identity, also led to delayed care-seeking, travel to more distant clinics and missed opportunities for appropriate services. Participants described experiences of violence and lack of police protection as well as mental health challenges. Key informants, however, reflected on their duty to provide non-discriminatory services to all Swazis regardless of personal beliefs.

**Conclusions:**

Intersectionality provides a framework for understanding the experiences of dual stigma and discrimination faced by MSM living with HIV in Swaziland and highlights how programmes and policies should consider the specific needs of this population when designing HIV prevention, care and treatment services. In Swaziland, the health sector should consider providing specialized training for healthcare providers, distributing condoms and lubricants and engaging MSM as peer outreach workers or expert clients. Interventions to reduce stigma, discrimination and violence against MSM and people living with HIV are also needed for both healthcare workers and the general population. Finally, research on experiences and needs of MSM living with HIV globally can help inform comprehensive HIV services for this population.

## Introduction

Globally, men who have sex with men (MSM) have substantially higher levels of HIV infection than men in the general population [1]. This is true even in the generalized HIV epidemics of sub-Saharan Africa, where MSM have more than three times the HIV prevalence of general population adult males on average [1]. Despite the knowledge that MSM are more likely to be infected with HIV across settings, there has been little investigation of the experiences of MSM who are living with HIV in sub-Saharan Africa.

Positive health, dignity and prevention is a framework used to highlight health and social justice issues for people living with HIV (PLHIV) [2,3]. The primary goals of positive health, dignity and prevention are “to improve the dignity, quality, and length of life of people living with HIV; which, if achieved will, in turn, have a beneficial impact on their partners, families, and communities, including reducing the likelihood of new infections” [2]. This framework builds upon earlier concepts of “positive prevention” and “prevention with positives,” which highlighted the importance of ensuring the health of PLHIV and engaging PLHIV in HIV-prevention efforts [4–6]. However, positive health, dignity and prevention situates living with HIV within a human rights framework and focuses on the importance of understanding and addressing structural constraints. It also considers the role of stigma and discrimination, which Parker and Aggleton [7] describe as social processes related to social inequality, power and oppression through which some groups are structurally excluded in society. Stigma has often been defined based on the classic work of Goffman as the social devaluation of a person based on a “significantly discrediting” attribute [8], while discrimination has been defined as behaviour resulting from prejudice [9]. Both stigma and discrimination are common in relation to both HIV and same-sex relationships.

In Swaziland, HIV prevalence in reproductive-age adults is among the highest in the world at 26.1% [10]. UNAIDS classifies Swaziland as a generalized HIV epidemic and, to date, the response to HIV in Swaziland has largely focused on the general population. Recently, the first surveillance of HIV prevalence and associated risk factors among MSM in Swaziland was conducted and showed a high burden of HIV among Swazi MSM, comparable to that of men in the general population [11]. However, same-sex behaviour is criminalized in Swaziland, and little attention has focused on the experiences of MSM who are living with HIV in this setting. Indeed, we identified just one peer-reviewed article focusing on HIV-positive MSM in sub-Saharan Africa. Cloete *et al*. [12] conducted a survey on HIV-related stigma and discrimination among a convenience sample of both HIV-positive MSM and men who have sex with women in Cape Town, South Africa. The survey found that internalized HIV-related stigma was high among all participants. Overall, MSM reported slightly greater social isolation and discrimination due to their HIV status, but these differences generally did not reach statistical significance.

In this study, we sought to explore the positive health, dignity and prevention needs of MSM who are living with HIV in Swaziland to inform HIV prevention, care and treatment services for this population. To our knowledge, this is one of the first qualitative studies to examine these issues among HIV-positive MSM in sub-Saharan Africa. As such, findings could inform the design and implementation of programmes for MSM living with HIV in Swaziland and similar settings.

## Methods

A qualitative approach was used to address the study aims. Methods included key informant interviews, in-depth interviews with HIV-positive MSM, and focus groups with MSM community members.

Key informants were selected if they had experience with MSM and lesbian, gay, bisexual and transgender (LGBT) populations or with HIV-related services in Swaziland. Sixteen key informants were interviewed, including HIV programme planners, policy makers, clinicians and LGBT community leaders. Interviews were semi-structured and employed a field guide to direct the conversation and stimulate probing. Participants were asked to describe the situation of MSM in their communities, their knowledge of existing services for MSM and PLHIV and their suggestions for how services could better meet the needs of MSM.

In-depth interviews were conducted with 20 MSM living with HIV interviewed twice each for a total of 40 interviews. Recruitment was conducted through a variety of settings and organizations, including HIV clinics; PLHIV networks; LGBT and MSM community organizations; and HIV prevention, care and treatment services. Participants were asked about the experiences of MSM generally in their communities; MSM social networks; personal and community experiences with HIV prevention, care and treatment services; experiences with stigma and discrimination; and suggestions for how services, interventions and messages could be better tailored for MSM.

Focus groups were conducted with MSM to gather a broader community perspective on the study topics; HIV status was not asked for reasons of confidentiality. Three focus groups were conducted with 26 MSM (4, 9 and 13 participants in each group). Topics covered were similar to interviews.

All interviews and focus groups were conducted in a private setting in either English or SiSwati and lasted approximately one to two hours. MSM were interviewed by a Swazi familiar with the local LGBT community who received training in qualitative research, while key informants were interviewed by an American masters-level research assistant with qualitative training living in Swaziland.

### Qualitative data analysis

Analysis of qualitative data was conducted through identification of recurrent patterns and themes following Crabtree and Miller's five steps in qualitative data analysis, or the “interpretive process” [13]. These steps are: (i) describing, (ii) organizing, (iii) connecting, (iv) corroborating and (v) representing. These steps form part of an iterative process which starts by re-examining the goals of the research and considering questions of reflexivity, then moves towards ways of highlighting, arranging and reducing texts to make connections through the identification of recurrent patterns and themes.

All interviews and focus groups were recorded, transcribed and translated into English. Debriefing notes immediately following each interview captured the interview context, theoretical issues, methodological issues and follow-up topics. Weekly meetings were held with all interviewers to discuss emerging themes and identify topics for further exploration to ensure an iterative process. After all data were collected, a full-day data analysis workshop was attended by representatives from LGBT groups, Ministry of Health (MOH) and National Emergency Response Council on HIV and AIDS (NERCHA) representatives, interviewers, clinicians and other stakeholders. This workshop devoted individual time to read de-identified transcripts to identify themes, then group time to categorize and discuss emerging themes and implications. Following the workshop, a codebook was developed by four study team members working together until agreement was reached. Codes were selected based on *a priori* topics of interest (research questions), themes identified during the data analysis workshop and emergent themes from transcripts. Codes were then applied using the computer software package Atlas.ti (version 5.2, Scientific Software Development GmbH, Eden Prairie, MN). The coded text was read to identify further themes or patterns and memos were created for key themes, which were developed into the findings presented here.

### Ethical considerations

All participants provided oral informed consent prior to participation, and referrals to clinical and counselling services were provided as needed. Study staff members were trained on sensitivity issues around HIV and MSM. A study advisory board, including representation from the LGBT community, implementing partners and government, reviewed the study protocol and interview guides and provided ongoing advice to the management and execution of the study. Ethical review and approval for this study was received from the Scientific and Ethics Committee of the Swaziland MOH and the Johns Hopkins Bloomberg School of Public Health in the United States.

## Results

### Dual stigma and disclosure of sexual identity and HIV status

The predominant theme across interviews was the significant and multiple forms of stigma and discrimination faced by MSM living with HIV in Swaziland. MSM reported experiencing stigma and discrimination related to both their HIV status as well as their sexual identity.

Same-sex behaviour is both criminalized and heavily stigmatized in Swaziland. MSM reported experiencing significant stigma, discrimination and rejection as a result of their sexual identity. One man, when asked if he had ever experienced stigma or discrimination as a result of being gay, responded, “A lot, several times, too many times.”

As a result of these experiences, and fear of similar stigma and rejection, many participants said they had not disclosed their sexual identity to anyone except other MSM. “That is my secret and I'm not planning to tell anyone in my family,” explained one. Participants worried about negative reactions, rejection and abuse if they disclosed. One man, when asked what would happen if he disclosed his sexuality to his friends or family, responded, “I would not even dare. It would be like being in a devil's den.” Others worried more about disappointing their loved ones by not conforming to social norms. One MSM asked,

Do you know this SiSwati saying that goes, ‘you have to have a heart for the other person’? … We always put the next person before [ourselves] … So we hardly want to disappoint the next person with being me, being myself and being comfortable with myself and insisting that I should be accepted, you know. We want to always conform [to] what society expects.

However, some participants had disclosed their sexual identity to family members or friends and had found acceptance, often after some initial difficulty.

Men also described stigma related to their HIV status. One participant described “the abuse we are subjected to” as “stigma, you see, that once you are HIV-positive, people think that you have AIDS. And also, that people have not accepted and they still do not know what HIV is.” Experiences or fear of HIV-related stigma prevented many MSM from disclosing their HIV status to family, friends and sexual partners. Lack of disclosure led to challenges with antiretroviral drug (ARV) adherence, hiding medications and a lack of social support for care-seeking and adherence to care and ARVs.

Participants selectively disclosed either their HIV status or their sexual identity to different individuals based on their anticipated reaction. For example, participants said they might disclose their HIV status to family members as they anticipated receiving some material or emotional support as a result, but they might not disclose their sexual identity to those same family members due to fear of rejection or a negative reaction.

### Violence and lack of police protection

Violence was also a common experience for MSM. MSM reported violence from a range of individuals. One man noted that some MSM “are killed for being gay, others are assaulted and others are chased away from home and disowned.” Due to the criminalized nature of same-sex behaviour in Swaziland, many MSM felt they had no recourse to bring incidents of discrimination or violence to the authorities. Furthermore, many had experienced a lack of police protection as a result of their sexuality. One participant described such an incident:

Participant (P): I was actually with a friend of mine in Manzini and we went to the butchery for a braai [barbecue], and when we got there, umm, there were these people who were, like, sitting outside at the car park. They were just rude and they started insulting us and we didn't try to defend ourselves, try to explain anything, and they went on, like, we are gay, we have to be beaten up, the gayness should be beaten out of us. We just ignored them and they attacked one of my friends we were with, they started beating him and he was bleeding.

Interviewer (I): Really.

P: Like for real, he bled to the point where we had to go to the hospital and we obviously went to lay a charge. And the police were kind of ‘occupied’, they didn't have the time to go and find these people that have beaten my friend.

I: Why did the police act in that way? Did you narrate to them what happened?

P: We sure did, but I think it's because we told them how the whole thing started – they called us names because they say we are gay. And I think also the police could tell that we are [gay], so they thought there was no case there.

### Stigma from healthcare settings

The stigma associated with being an MSM was the predominant barrier to accessing healthcare services for MSM living with HIV. Both perceived and experienced stigma in healthcare settings led to a lack of care-seeking behaviour. As one participant described it,

When they say ‘bring your partner’, and then you bring the same sex partner, they are like, ‘yah, this is why you are having this [HIV], this is why’, and they will be throwing words at you … so then you get embarrassed, sometimes you'll decide to leave without being treated, and where are you taking that sickness to?

Another participant, when asked how the needs of MSM differed from PLHIV in general, explained that the main difference was how forthright MSM could be about issues related to their sexuality:

I think they are different in the sense that for those who are straight they are open and they communicate easily about sex issues. As for us gays, it's difficult unless you have someone you can talk to and give you advice as to what you can do when you have some health issues. As for people in general, with them it's easy for them to go to hospital, but with us it's difficult. You can't say it's painful in your anus – what will you say the cause for that is?

This participant continued by noting that this influenced care-seeking behaviour, as he would delay care-seeking or self-medicate to avoid disclosure:

I: What happens, so you end up not going there [to the hospital]?

P: I just stay at home and you find that this thing becomes complicated. When this thing becomes complicated, you find that maybe you go to the pharmacy and they tell you that this thing is at an advanced stage.

Other men said they travelled long distances to seek HIV care at clinics where they either were not known personally or where they did not experience stigma and discrimination.

P: Even at the hospital, they interviewed me, then there were changes and I could tell that they wanted me to reveal what type of person I am. Since then I stopped fetching my drugs there. I now go to another clinic which is far away from home. I drive all the way to fetch my tablets instead of taking them locally.

I: Really, why is it so?

P: Because I thought there is problem at the local clinic since I am gay. So I decided to change … They treat us like small devils, as if we are the one who are spreading the HIV virus.

However in a few cases, MSM did disclose their sexual identity to healthcare providers and reported positive and supportive reactions, particularly from non-governmental HIV testing and counselling sites.

Fear of stigma also shaped the type and nature of counselling that MSM received in healthcare settings, particularly regarding offering services to sexual partners. MSM, as well as key informants, noted that in clinical services such as HIV testing and treatment, providers’ questions about HIV prevention generally assume heterosexuality. Providers would ask MSM to bring their wives into the clinic to be tested for HIV. Due to fear of stigma, MSM would often simply state that they did not have a wife, but would not mention their male sexual partners.

Finally, participants reported mistreatment by staff and lack of confidentiality at clinics due to being HIV-positive. These negative experiences were particularly experienced when picking up ARVs, leading one MSM to say that “the ARVs end up being an inconvenience [rather] than helping you.” Some men felt that PLHIV in general were treated poorly by healthcare workers. “You really feel that you are different from other people,” explained one. However, others felt that at least some healthcare workers provided high-quality care to PLHIV, and that MSM were not necessarily treated any differently from other PLHIV.

### Mental health challenges

Many MSM said that living with a stigmatized sexual identity and a challenging, stigmatized disease led to feelings of depression as well as self-stigma or shame. “To be like this to me seems like I was created for nothing on earth,” said one, “because there is nobody who is happy about me at home and at school.”

The initial receipt of an HIV-positive diagnosis was emotionally devastating for many participants. Participants described feelings of depression and anger. They also said that others had even more difficult coping. “Some of them they commit suicide because they can't accept their status,” said one MSM, “because no one can accept them as they are gay and positive.” Some participants said feelings of self-stigma led them to drink alcohol as a coping mechanism.

[After testing HIV-positive], I was very much hurt so much that I decided to devote myself to drinking alcohol. I was drinking every day, and there was not a day that went by without me drinking.

However, over time, many participants said they came to accept their HIV status and learn to cope with the disease. MSM also reported that they had difficulty accepting their sexuality. Some described shame related to having sexual feelings for other men.

Participants reported receiving emotional support from a variety of sources. One MSM said he went to his pastor for support, while another derived comfort from religion but had not disclosed or discussed his life with his church. Only one participant mentioned going to formal counselling services, saying he and his partner saw a private counsellor who knew they were gay. However, most received support from partners, friends or family to whom they had disclosed either their HIV status or their sexual identity.

### Preventing HIV transmission to sexual partners and the context of MSM relationships

MSM in this study were very aware of the need to prevent onward HIV transmission to sexual partners. Many discussed how they had changed their behaviour after being diagnosed with HIV in order to reduce transmission risk to others by using condoms and reducing the number of partners. However, others reported continued risk behaviour, often linked to alcohol use. As one participant put it, “most of the time we have sex without a condom it is when we are drunk.”

Poverty and lack of economic opportunities also shaped risk behaviours. Participants reported that some members of the MSM community were not necessarily gay, but engaged in transactional sex with men to support themselves financially. However, the majority of our participants identified as gay, and many said they were in long-term, monogamous partnerships with other men.

Some MSM felt that the clandestine nature of MSM relationships in Swaziland may lead to greater numbers of and more casual types of partnerships. MSM described many of their partners as bisexual or having female girlfriends and wives, possibly to fulfil cultural expectations. Furthermore, MSM said that their relationships are often kept secret and therefore families do not play a role in relationship counselling and peacekeeping as they might for heterosexual couples.

Usually in our community we have short-term relationships. These relationships are caused by the fact that there is nothing bonding those people. And maybe the community, the parents or relatives are not involved in our relationships. And then if I have got a problem with my boyfriend, if I say it's over, it's over … you are not able to go tell your parents or relatives … if people are informed either way about such people [MSM] in the community, if there is a relationship going on with his parent, the parent will be able to intervene either way, and those relationships will sustain.

### Improving positive health, dignity and prevention services for MSM

MSM said that societal acceptance and stigma reduction would be the most important way to improve services for MSM living with HIV. As one man stated, “If we can be recognized and they can know that there are people who are living this kind of life and they can know how they can reach us in terms of programmes and services.” Participants knew that same-sex relationships were more accepted in neighbouring South Africa and hoped that social norms in Swaziland might shift in a similar direction. They also discussed the organizations working openly for LGBT health and rights in South Africa and noted that the lack of such formal organization in Swaziland limited the ability to develop an effective and appropriate response to HIV for MSM.

Participants held a variety of opinions on how best to tailor existing interventions and services for MSM. Some participants suggested developing special clinics or services for HIV-positive MSM. Others worried that targeted services would reinforce stigma. One potential consideration was including MSM living with HIV as “expert clients” to help navigate HIV treatment services. Participants said less about mental health services; just a handful of interviewees said that increasing access to counsellors would be helpful, as existing HIV care and treatment providers were overworked and did not have time to provide in-depth counselling for PLHIV.

Currently, as there are essentially no HIV-prevention services for MSM in Swaziland, participants suggested a “training of trainers” model, whereby trusted MSM community members could be trained in HIV-prevention messages particularly relevant for MSM and could then share those messages with others in their community. MSM also suggested continued or expanded distribution of condoms and particularly lubricant to prevent condom breakage.

Several participants, both MSM and key informants, said that healthcare workers should be trained on issues related to MSM. As one key informant explained, “Even their procedures manuals should have information on how to handle MARPS [most at-risk populations, including MSM].” Importantly, key informants in this study consistently said that regardless of personal belief, they had an ethical responsibility to provide services to everyone, equally. “As a [member of the] health sector, my belief is non-discriminatory services to all the members of the population, and issues of legality and everything rest with the Ministry of Justice,” said one. Another stated,

Even though I don't approve of what they are doing … as a public health officer, I have to make sure that they have access to health services. I don't have to judge them. I don't have to give my views on what they are doing. But my duty is to make sure that they have access to services … whatever their sexual orientation is, they are human beings, they are Swazi.

## Discussion

This study is among the first studies to examine the positive health, dignity and prevention needs of HIV-positive MSM in sub-Saharan Africa. We found that a social and structural context characterized by significant and multiple stigmas was key to understanding these needs. Dual stigma related to both sexual identity and HIV status led to selective disclosure or lack of disclosure of both identities, and consequently a lack of social support for care-seeking and medication adherence. Perceived and experienced stigma from healthcare settings, particularly around sexual identity, also led to delayed care-seeking, travel to more distant clinics and missed opportunities for appropriate services. These findings support and extend findings from other sub-Saharan African settings that discrimination reduces the willingness of MSM to access services [14–16]. The lack of support from friends, relatives and society for same-sex relationships was described as weakening these relationships, leading to greater numbers of sexual partners as well as relationships with women to “hide” same-sex behaviours, potentially further increasing HIV risk. This finding similarly echoes research from the United States suggesting that psychosocial health problems may increase HIV risk among MSM, leading to a “syndemic” [17]; such findings highlight the need to approach HIV prevention within the context of overlapping health problems [18].

Intersectionality is a theoretical framework that examines the relationship or “intersection” between multiple forms of oppression and discrimination due to social categorizations such as race, class or gender [19]. MSM living with HIV experience the dual stigma of being a sexual minority and having a stigmatizing illness. Intersectionality posits that these multiple stigmas are not experienced independently, but that they interact in complex ways to create disparity and social inequality in health outcomes [19]. We found that MSM living with HIV described dual stigma as an overwhelming burden in their lives which influenced multiple aspects of their health and relationships. Considering the needs of MSM living with HIV in this intersectionality framework provides the deepest understanding of their experience.

Intersectionality also highlights the ways in which individual experiences with stigma reflect larger social structures that create and sustain inequality. Participants in our study experienced outright discrimination, stigma and violence against MSM and PLHIV. However, because sexual identity can be concealed, they often encountered situations in which they were assumed to be heterosexual – assumptions which they did not correct due to fear of discrimination. For example, in healthcare settings, many providers assumed their clients were heterosexual and provided services accordingly. For our participants, these assumptions led to missed opportunities for appropriate counselling services tailored to their individual needs and risks, as well as missed opportunities for offering important services, such as HIV testing and counselling, to their sexual partners. Although the World Health Organization couples HIV testing and counselling guidelines support offering these services to same-sex couples [20], in practice, most couples HIV testing and counselling services in sub-Saharan Africa focus exclusively on steady, heterosexual partnerships and fail to consider same-sex relationships. Although individual providers may offer supportive services for same-sex partners, a more comprehensive approach is needed to incorporate training on same-sex relationships into couples HIV testing and counselling programmes.

Currently, services for MSM living with HIV in Swaziland are essentially non-existent. This is unsurprising, given the lack of data on MSM and HIV risk in Swaziland until very recently and the criminalization of same-sex behaviour in Swaziland. Research has documented a strong correlation between criminalization of same-sex behaviour and lack of investment in services for MSM globally [21]. However, MSM have unique healthcare needs [22], and even in rights-constrained settings, comprehensive HIV services for MSM can and should be provided [23]. Our findings suggest the beginnings of political will among healthcare workers, key stakeholders at the government and local levels and the MSM community to provide these services. Key informants in particular reflected on their duty to provide services to all Swazis in a non-discriminatory manner. These beliefs can provide a foundation for establishing comprehensive HIV services, including both prevention *and* care and treatment services, for MSM. In fact, this research helped, in part, to catalyze the official registration of an NGO, Rock of Hope, dedicated to key population rights in Swaziland, including LGBT rights, which has been invited to engage with the country's key population policy technical working group addressing HIV among MSM and other key populations. The technical working group is under the auspices of the Swaziland National AIDS Programme (SNAP), a programmatic body under the MOH. Other implementing partner organizations providing HIV-related services have indicated they would be open to developing services for MSM. In this changing political and institutional context, there appears to be a genuine possibility of government, NGO and civil society collaboration to develop an effective and comprehensive response to the HIV epidemic among MSM in Swaziland.

This study provides unique information about the needs of MSM living with HIV in a sub-Saharan African context with high HIV disease burden. Conducting multiple interviews with MSM living with HIV and working closely with local LGBT groups increased the comfort level of our participants and their willingness to participate in this study. However, MSM participants were still discussing very sensitive, stigmatized and illegal behaviours, and they may not have fully opened up to interviewers. Data were collected largely from MSM in urban centres due to reliance on existing networks; this may limit transferability of the findings to rural MSM or those without strong MSM social networks.

## Conclusions

The intersecting stigmas of sexual identity and HIV status shaped multiple facets of the lives of MSM living with HIV in Swaziland. Intersectionality provides a framework for understanding these experiences and highlights how programmes and policies should consider the specific needs of this population when designing HIV prevention, care and treatment services. In Swaziland, programmes should consider tailored multi-level interventions that address these unique needs at the policy, societal and healthcare delivery levels. At the policy level, the health sector in Swaziland is already initiating important research to examine the epidemiology and service delivery needs of MSM; findings from this research should be incorporated into the national HIV response. For Swazi society in general as well as healthcare providers, interventions to reduce stigma, discrimination and violence against MSM and PLHIV are needed. The health sector should also consider distributing condoms and lubricant for MSM, training healthcare providers in the specific health needs of MSM and engaging MSM as peer outreach workers or expert clients in both prevention activities and clinical services. Finally, further research examining the experiences and needs of MSM living with HIV globally is required to improve comprehensive HIV services for this population.

## References

[1] Beyrer C, Baral SD, van Griensven F, Goodreau SM, Chariyalertsak S, Wirtz AL (2012). Global epidemiology of HIV infection in men who have sex with men. Lancet.

[2] GNP+, UNAIDS (2011). Positive health, dignity and prevention: a policy framework.

[3] GNP+, UNAIDS (2009). Positive health, dignity and prevention. Technical consultation report.

[4] Kennedy CE, Medley AM, Sweat MD, O'Reilly KR (2010). Behavioural interventions for HIV positive prevention in developing countries: a systematic review and meta-analysis. Bull World Health Organ.

[5] Auerbach JD (2004). Principles of positive prevention. J Acquir Immune Defic Syndr.

[6] Kalichman SC (2004). Positive prevention: reducing HIV transmission among people living with HIV/AIDS.

[7] Parker R, Aggleton P (2003). HIV and AIDS-related stigma and discrimination: a conceptual framework and implications for action. Soc Sci Med.

[8] Goffman E (1963). Stigma: notes on the management of a spoiled identity.

[9] Earnshaw VA, Chaudoir SR (2009). From conceptualizing to measuring HIV stigma: a review of HIV stigma mechanism measures. AIDS Behav.

[10] World Bank (2009). Swaziland HIV prevention response and modes of transmission analysis.

[11] Baral S, Grosso A, Mnisi Z, Adams D, Fielding-Miller R, Mabuza X (2013). Examining prevalence of HIV infection and risk factors among female sex workers (FSW) and men who have sex with men (MSM) in Swaziland.

[12] Cloete A, Simbayi LC, Kalichman SC, Strebel A, Henda N (2008). Stigma and discrimination experiences of HIV-positive men who have sex with men in Cape Town, South Africa. AIDS Care.

[13] Crabtree BF, Miller WL (1999). Doing qualitative research.

[14] Fay H, Baral SD, Trapence G, Motimedi F, Umar E, Lipinge S (2011). Stigma, health care access, and HIV knowledge among men who have sex with men in Malawi, Namibia, and Botswana. AIDS Behav.

[15] Lane T, Mogale T, Struthers H, McIntyre J, Kegeles SM (2008). ‘They see you as a different thing’: the experiences of men who have sex with men with healthcare workers in South African township communities. Sex Transm Infect.

[16] Rispel LC, Metcalf CA, Cloete A, Moorman J, Reddy V (2011). You become afraid to tell them that you are gay: health service utilization by men who have sex with men in South African cities. J Public Health Policy.

[17] Parsons JT, Grov C, Golub S (2012). Sexual compulsivity, co-occurring psychosocial health problems, and HIV risk among gay and bisexual men: further evidence of a syndemic. Am J Public Health.

[18] Halkitis PN, Woliski RJ, Millett GA (2013). A holistic approach to addressing HIV infection disparities in gay, bisexual, and other men who have sex with men. Am Psychol.

[19] Bowleg L (2012). The problem with the phrase women and minorities: intersectionality – an important theoretical framework for public health. Am J Public Health.

[20] World Health Organization (2012). Guidance on couples HIV testing and counselling including antiretroviral therapy for treatment and prevention in serodiscordant couples: recommendations for a public health approach.

[21] AMFAR, JHSPH (2012). Achieving an AIDS-free generation for gay men and other MSM: financing and implementation of HIV programs targeting MSM.

[22] Mayer KH, Bekker LG, Stall R, Grulich AE, Colfax G, Lama JR (2012). Comprehensive clinical care for men who have sex with men: an integrated approach. Lancet.

[23] Beyrer C, Sullivan PS, Sanchez J, Dowdy D, Altman D, Trapence G (2012). A call to action for comprehensive HIV services for men who have sex with men. Lancet.

# Sub article: Epidemiology of HIV among female sex workers, their clients, men who have sex with men and people who inject drugs in West and Central Africa

**DOI:** 10.7448/IAS.16.4.18751

**Published:** 2013-12-02

**Authors:** Erin Papworth, Nuha Ceesay, Louis An, Marguerite Thiam-Niangoin, Odette Ky-Zerbo, Claire Holland, Fatou Maria Dramé, Ashley Grosso, Daouda Diouf, Stefan D Baral

**Affiliations:** 1 Department of Epidemiology, Johns Hopkins Bloomberg School of Public Health Baltimore, MD, USA; 2 Joint United Nations Programme on HIV/AIDS, Banjul, Gambia; 3 Programme de Lutte Contre le SIDA Chez Les Populations Hautement Vulnérables (PLS-PHV) Ministère de la Sante et de la lutte contre le SIDA, Abidjan, Côte d'Ivoire; 4 Programme d'Appui au Monde Associatif et Communautaire (PAMAC), Ouagadougou Burkina Faso; 5 Enda Santé, Dakar, Senegal

**Keywords:** men who have sex with men, sex work, people who inject drugs, HIV epidemiology, West Africa, Central Africa, prevalence, risk factors

## Abstract

**Introduction:**

The West and Central Africa (WCA) sub-region is the most populous region of sub-Saharan Africa (SSA), with an estimated population of 356 million living in 24 countries. The HIV epidemic in WCA appears to have distinct dynamics compared to the rest of SSA, being more concentrated among key populations such as female sex workers (FSWs), men who have sex with men (MSM), people who inject drugs (PWID) and clients of FSWs. To explore the epidemiology of HIV in the region, a systematic review of HIV literature among key populations in WCA was conducted since the onset of the HIV epidemic.

**Methods:**

We searched the databases PubMed, CINAHL and others for peer-reviewed articles regarding FSWs, MSM and PWID in 24 countries with no date restriction. Inclusion criteria were sensitive and focused on inclusion of any HIV prevalence data among key populations. HIV prevalence was pooled, and in each country key themes were extracted from the literature.

**Results:**

The search generated 885 titles, 214 abstracts and 122 full articles, of which 76 met inclusion and exclusion criteria providing HIV prevalence data. There were 60 articles characterizing the burden of disease among FSWs, eight for their clients, one for both, six for MSM and one for PWID. The pooled HIV prevalence among FSWs was 34.9% (*n=*14,388/41,270), among their clients was 7.3% (*n=*435/5986), among MSM was 17.7% (*n=*656/3714) and among PWID from one study in Nigeria was 3.8% (*n=*56/1459).

**Conclusions:**

The disproportionate burden of HIV among FSWs appears to be consistent from the beginning of the HIV epidemic in WCA. While there are less data for other key populations such as clients of FSWs and MSM, the prevalence of HIV is higher among these men compared to other men in the region. There have been sporadic reports among PWID, but limited research on the burden of HIV among these men and women. These data affirm that the HIV epidemic in WCA appears to be far more concentrated among key populations than the epidemics in Southern and Eastern Africa. Evidence-based HIV prevention, treatment and care programmes in WCA should focus on engaging populations with the greatest burden of disease in the continuum of HIV care.

## Introduction

The sub-region of West and Central Africa (WCA) is the most populous of sub-Saharan Africa (SSA), with a combined population of roughly 356 million [1]. The region possesses a distinct cultural, economic and historical diversity. The majority of countries purport French as their national language, while English is the state language for four countries, and Spanish and Portuguese are both spoken within the region. Fifteen of the countries in WCA are classified by the World Bank Atlas method as low income (>US$1025), including Benin, Burkina Faso, Cape Verde, Central African Republic, Chad, the Democratic Republic of Congo (DRC), the Gambia, Guinea, Guinea-Bissau, Liberia, Mali, Mauritania, Niger, Sierra Leone and Togo [2]. Côte d'Ivoire, Cameroon, Ghana, Nigeria, the Republic of Congo, Senegal and São Tomé and Príncipe are categorized as low-middle income (US$1026 to US$4035) [2]. One country in the region is upper-middle income (Gabon), and one is ranked as a high-income country (Equatorial Guinea), mainly due to newly found oil reserves and a population under 1 million [2].

Historically and economically multifarious, the region has not been immune to the HIV epidemic. The first reported cases of HIV emerged in the mid-1980s, and national surveillance bodies such as National AIDS Committees (NACs) were established over the subsequent decade [3]. Early phylogenetic subtyping revealed unique regional dynamics, with both HIV-1 and HIV-2 circulating, and the majority of global cases of HIV-2 found in West Africa. Concurrently, the origins and greatest subtype diversity of HIV-1 were reported in Central Africa [4] (Figure 1).

**Figure 1:** Map of West and Central Africa.

Nevertheless, regional epidemiological reporting has traditionally been immersed in the overall context of SSA. Trends in the HIV epidemic show that SSA possesses the highest burden of HIV, and 69% of the global population of people living with HIV reside within its borders [23.5 million (22.1–24.8 million)] [5, 6]. While these statistics show an important burden of disease on the continent, they mask disparities in HIV epidemics regionally [7]. Countries in East and South Africa report consistently generalized epidemics among reproductive-age adults (ages 15–49), which is defined through the Joint United Nations Programme on HIV/AIDS (UNAIDS) criteria as HIV prevalence consistently higher than 1% in antenatal clinics [8, 9]. Nine out of the 15 Southern African Development Community (SADC) members report national prevalence over 10% [5, 6, 10]. Reproductive-age adult estimates are as high as 25.9% in Swaziland and 24.8% in Botswana [11]. Comparatively, national prevalence in WCA has remained low or moderate since HIV surveillance reporting began, with current general-population estimates ranging from 0.02 to 4.5% [5, 6, 12]. Twelve countries in the sub-region report national prevalence under 2% [5]. Consequently, the majority of these countries’ HIV epidemics are classified as mixed, concentrated or borderline generalized [6, 12].

The international community has recently noted that classifications of the HIV epidemic based on prevalence data often limit understanding of the complexity of transmission and appropriate prevention strategies. However, concentrated epidemics have historically been defined as occurring in countries where HIV prevalence is consistently higher than 5% in at least one subgroup within the population, but less than 1% in antenatal clinics [7, 9]. These subgroups are generally considered to be female sex workers (FSWs), men who have sex with men (MSM) and people who inject drugs (PWID) [7, 13]. There is less clarity around mixed epidemics, although these are generally agreed to be low-level generalized epidemics ranging from 2 to 5% HIV prevalence in the general population, and high transmission rates in subgroups of the population [7]. Based on this, the HIV epidemics in countries in WCA are predominantly mixed or concentrated.

Researchers have suggested that the complexity of the regional dynamics in WCA has not been dissected adequately [12, 14–16]. Underlying drivers such as migration patterns, subtype diversity, significant regional variations of the disease and at-risk populations are understudied [11, 12, 16–19]. In an era where the global spread of HIV is on the decline, data are progressively emerging to show sustained or expanding transmission in populations at high-risk for HIV [15, 20–22]. However, national surveillance systems, particularly in low and middle-income countries, remain constructed on population-level studies such as the Demographic and Health Survey and antenatal care surveillance data [6, 13]. These methods provide a global overview of basic risk factors associated with transmission, but they do not capture data characterizing sex work and other transactional or compensated sex, same-sex practices and drug use outside of alcohol consumption, all of which are demonstrated high-risk factors and contributors to the acquisition and transmission of HIV [11, 21, 23].

Globally, surveillance shows that groups such as FSWs, their clients, MSM and PWID sustain a higher burden of disease in concentrated epidemics and substantially contribute to new infections annually [4, 7, 18, 22, 24]. In settings such as Southeast Asia and Latin America, general-population HIV prevalence remains similar to that of WCA, and a higher burden of disease is observed among key populations. For example, Pakistan and Indonesia report 25% and 35% prevalence among PWID, respectively [25]. Vietnam and Chile report an HIV prevalence rate of 15% and 20% among MSM, respectively [25, 26]. Myanmar (Burma) reports a prevalence of 10% among FSWs, and Brazil reports 4.9% [25, 26]. All of these reported levels are roughly five to thirty times higher than general-population prevalence in the specific countries listed [25, 26]. National-level responses on these continents have included programmes for key populations, and noteworthy advances in the reduction of new infections have been reported over time [27, 28]. In contrast, WCA reports partial or sporadic data for key populations and limited government-level policies defining key population treatment and prevention needs [5]. National surveillance and programming in WCA subsequently remain rooted in broad HIV prevention messaging and approaches similar to those seen across East and South Africa such as prevention of mother-to-child transmission (PMTCT) and non-targeted community-based behaviour change programmes [5, 7].

Lessons learned from other contexts such as Southeast Asia and Latin America, where limited prevalence of HIV among average-risk reproductive-age adults also exists, require us to examine the epidemiology of the HIV epidemic in WCA [11, 29]. This systematic review aims to complete a historic, situational and epidemiological analysis of the burden of disease among key populations in 24 countries located in WCA.

## Methods

The US National Library of Medicine's MEDLINE database, one of the most comprehensive sources of healthcare information in the world, was searched using the PubMED interface to obtain biomedical markers for any of the three key populations: FSWs, MSM or PWID. The study objectives specified the need for epidemiologic studies that report biological endpoints (HIV prevalence) with defined methods; thus, it was decided a priori that MEDLINE would be sufficient. However, a sensitivity assessment was employed using the same search strategy to explore EBSCOhost CINAHL Plus, PsycINFO, Ovid, SocioFile and Popline, and no additional data points were obtained which met the defined inclusion and exclusion criteria. Google and Google Scholar were searched for contextual information and non-peer-reviewed literature. The Preferred Reporting Items for Systematic Reviews and Meta-Analyses (PRIMSA) guidelines were referenced for the development of the search protocol and study reporting structure [30, 31].

The medical subject headings (MeSH terms) for HIV and AIDS and key terms relating to “sex work,” “men who have sex with men” and “intravenous drug use” were cross-referenced with terms associated with 16 West African countries: the 15 countries of the Economic Community of West African States (ECOWAS: Benin, Burkina Faso, Cape Verde, Gambia, Ghana, Guinea (Conakry), Guinea-Bissau, Cote d'Ivoire, Liberia, Mali, Niger, Nigeria, Senegal, Sierra Leone and Togo) plus Mauritania. Eight Central African countries were included in the search: those in the Economic Community of Central African States (CEMAC: Cameroon, Chad, Equatorial Guinea, Central African Republic, Republic of Congo and Gabon), the Democratic Republic of Congo (DRC) and São Tomé and Príncipe. The search protocol was developed based on the objectives of this study and can be accessed as a Supplementary file with this manuscript.

The inclusion criteria for this study included reported HIV prevalence data for any of the three key populations, as well as clients of FSWs, in any of the 24 countries defined for this review. Publications were included if prevalence was listed in the article with sample size and sampling and HIV-testing methods described, regardless of the overall aim or topic of the study. Date of publication was not used as an inclusion criterion. Exclusion criteria included manuscripts not published in French, English or Spanish. Articles were downloaded and organized using Endnote (version X5), and data collection was finalized in April 2013.

### Screening and data abstraction

A title and abstract search protocol was utilized based on previously validated methods for systematic reviews [32]. At each step in the search protocol, the titles, abstracts and available data were appraised by two independent reviewers (LA and EP), and compiled and synthesized using standardized forms. During the title and abstract reviews, if either of the two reviewers considered the article relevant, it was included. Articles classified as relevant at the title review stage were downloaded for abstract and full-text evaluation. Data were independently extracted by two reviewers (LA and EP), then compared and consolidated for analysis.

Data, including sampling methods, HIV-1, HIV-2 and dual HIV-1 and -2 (HIV-1/2) infections with sample size and number of participants living with HIV, were detailed and coded by the two independent reviewers (LA and EP). Information was categorized by key population studied, sampling techniques, country or countries, sample size, number of study participants living with HIV and notes. Discrepancies in abstracted data from the two reviewers were assessed by a third reviewer independently evaluating the article (SB), as was the final consolidated database (CH).

## Results

Our search generated 995 citations, including 885 unique titles with dates of publication from 1987 to 2013 (Figure 2). Based on the inclusion criteria, 122 full articles were reviewed for data extraction, and 76 of these contained relevant data for at least one of the key populations defined. HIV prevalence data for at least one key population existed in 13 of the 24 countries included in the search (54.2%). Eleven of these countries were located in West Africa, and two countries were in Central Africa (DRC and Cameroon).

**Figure 2:** Flow chart of search findings and included studies.

The majority of publications were assessments regarding FSWs (78.9%, 60/76), and another 10.5% (8/76) provided HIV prevalence data for their clients. One publication provided prevalence data for FSWs and well as clients of FSWs in Togo [33]. Thus, 90.8% (69/76) of the publications included in this study were related to FSWs, representing 41,270 FSWs across 13 countries and 5,986 clients of FSWs across 6 countries.

Two countries (Senegal and Nigeria) had published HIV prevalence data among MSM, and one seroprevalence study was conducted among male sex workers (MSWs) in Côte d'Ivoire, which was included in the MSM pooled data for analysis [34]. A total of six publications combined for the three countries were found for MSM (7.9%, 6/76), and one publication was available with HIV prevalence data for PWID, totalling 3,714 MSM from three countries and 1,459 PWID represented in the region.

Results presented in Table 1 show a pooled HIV prevalence for the relevant key population(s) in each country, the 95% confidence interval (CI), and the date(s) of the publications retrieved per country. We include both HIV-1 and HIV-2 infections in the pooled prevalence data for the country, and, when possible, we display the division of HIV-1, HIV-2 and HIV-1/2 infections. The far-left data column in Table 1 displays the overall HIV prevalence among reproductive-age adults (15–49) per country as reported by UNAIDS’ most recent country-level surveillance data [6].

**Table 1:** Pooled prevalence data for female sex workers (FSWs), clients of FSWs, MSM and PWID per country

Country	Year of publication(s)	Key population	Pooled HIV prevalence % (95% confidence interval)	Pooled HIV prevalence (sample size *N*=)	HIV-1 prevalence % (sample size *N*=)*	HIV-2 prevalence % (sample size *N*=)	HIV 1 and 2 prevalence % (Sample size *N*=)	HIV prevalence % Among adults 15–49**	Reference
Benin	1992, 1997, 2001, 2002, 2007, 2009, 2012	FSWs	45.8 (44.2–47.4)	3,885	41.8 (*N*=498)	3.2 (*N*=498)	11.2 (*N*=498)	1.1	[35–41]
	2000, 2007	Clients	6.7 (5.6–7.8)	1,996					[42,43]
Burkina Faso	1998, 2002	FSWs	45.8 (42.5–49.1)	873				1.0	[44,45]
Cameroon	1991, 1995, 1998, 1998, 2001, 2009	FSWs	23.6 (22.4–24. 8)	4,679	22.9 (*N*=2260)	0.04 (*N*=2260)		4.5	[41,46–50]
Cote d'Ivoire	1987, 1988, 1992, 1995, 1995, 1997, 1998, 2000, 2002, 2012	FSWs	57.3 (56.1–58.5)	7,014	40.0 (*N*=5204)	2.7 (*N*=5204)	21.1 (*N*=5204)	3.2	[17,29,66–73]
	2003	Clients	13.5 (10.2–16.8)	423					[74]
	2012	Male sex workers	50.0 (40.0–60.0)	96					[34]
DRC	1988, 1988, 1991, 1998, 2007	FSWs	26.3 (24.6–28.0)	2,518				1.1	[51–55]
Gambia	1991, 1991, 1993	FSWs	28.5 (25.0–32.0)	627	1.3 (*N*=627)	25.2 (*N*=627)	2.1 (*N*=627)	1.3	[56–58]
	1992	Clients	6.1 (4.1–8.1)	558					[59]
Ghana	2000, 2001, 2012	FSWs	60.4 (58.3–62.6)	1,982	46.7 (*N*=1348)	2.2 (*N*=1348)	6.7 (*N*=1348)	1.4	[40,60,61]
	2004	Clients	12.3 (9.4–15.2)	497					[62]
Guinea	2010, 2010, 2011	FSWs	36.9 (34.5–39.3)	1,577				1.7	[63–65]
Mali	1988, 1998	FSWs	42.1 (37.3–46.9)	406	35.8 (*N*=176)	3.9 (*N*=176)	6.2 (*N*=176)	0.9	[75, 76]
Niger	1994, 1998, 2006, 2006	FSWs	31.2 (28.4–34.1)	1,017	29.2 (*N*=767)	0.9 (*N*=529)	2.0 (*N*=767)	0.5	[77–80]
Nigeria	1989, 1993, 1993, 1993, 1998, 2002, 2008, 2011, 2012, 2012, 2013	FSWs	24.3 (23.5–25.1)	10,769	13.5 (*N*=2291)	1.9 (*N*=2041)	1.8 (*N*=610)	3.2	[81–91]
	2013	PWID	3.8 (2.8–4.8)	1,459					[92]
	2011, 2012, 2013	MSM	15.1 (13.7–16.5)	2,676					[93–95]
Senegal	1992, 1996, 1997, 2003, 2007, 2009	FSWs	19.0 (17.9–20.1)	4,612	7.6 (*N*=4008)	10.1 (*N*=4008)	1.1 (*N*=4008)	0.5	[96–101]
	1997, 2003	Clients	4.6 (3.6–5.7)	1,515					[102,103]
	2005, 2009, 2010	MSM	21.7 (19.1–24.3)	942	18.1 (*N*=442)	0.5 (*N*=442)	2.9 (*N*=442)		[104,105]
Togo	2009	FSWs	36.2 (33.6–38.8)	1,311				2.9	[18]
	2009	Clients	7.9 (6.2–9.6)	997					[18]

* Where available, the distribution of HIV1, HIV2 and dual HIV1/2 infections in the available study or pooled per country is listed.

** UNAIDS country prevalence data 2012 (6).

### Female sex workers and their clients

Behavioural and seroprevalence studies in FSWs were conducted consistently over time; however, there was a significant lull in published data between 2002 and 2007. When pooled, the overall HIV prevalence for FSWs in WCA was 34.9% (95% CI 34.4–35.4) (Table 2). In the five countries with six or more publications, pooled HIV prevalence was high: 57.3% (*N*=7,014) in Côte d'Ivoire, 24.3% (*N*=10,769) in Nigeria, 45.8% (*N*=3,885) in Benin, 23.6% (*N*=4,679) in Cameroon and 19.0% (*N =*4,612) in Senegal. The pooled prevalence found among clients of FSWs was 7.3% (95% CI 6.6–8.0) (Table 2). Six countries had at minimum of one study reporting prevalence data for this demographic, with publications as early as 1992 and as late as 2009 (Table 1).

**Table 2:** Pooled HIV prevalence data for female sex workers, clients of FSWs, MSM and PWID in West and Central Africa

Key population	Pooled HIV prevalence (%)	95% Confidence interval (%)	Pooled sample size, *N*=	*n*=Living with HIV
Female sex workers (FSWs)	34.9	34.4–35.4	41,270	14,388
Men who have sex with men (MSM)	17.7	16.5–18.9	3,714	656
People who inject drugs (PWID)	3.8	2.8–4.8	1,459	56
Clients of FSWs	7.3	6.6–8.0	5,986	435

### Men who have sex with men

While this review revealed a paucity of data for MSM, the pooled HIV prevalence in this review was 17.7% (95% CI 16.5–18.9) for MSM in WCA (Table 2). No studies included were published earlier than 2005, and all but one were published after 2010. Three relevant Nigerian studies showed a pooled prevalence of 15.1% compared to 3.2% in adults of reproductive age [6, 93–95, 106]. Senegal's pooled prevalence was 21.7% compared to 0.5% in the adults of reproductive age [6, 104, 105, 107]. The study conducted in Côte d'Ivoire among MSWs reported 50.0% prevalence among a sample of 96 men in Abidjan [34]. Snowball, convenience, purposive and respondent-driven sampling were the primary recruitment methods used to obtain these data.

### People who inject drugs

One study included directly sampled PWID. The study found a slightly higher prevalence of HIV at 3.8% (95% CI 2.8–4.8), compared to 3.2% in the general population in Nigeria [6, 92]. The sample was recruited through respondent-driven sampling and mainly compromised of men (>90%) [6, 92].

#### Limitations

This study was conducted as a systematic review to understand the prevalence of key populations in WCA and compare historical HIV prevalence to general-population statistics. Data were obtained from peer-reviewed literature, and while this ensures some quality control, we acknowledge that some relevant data that exist in grey literature and other programmatic data may have been overlooked. Programmatic data were not included in this review as it was not possible to implement a standardized assessment of the quality of the methods used and to ascertain an overview of research sampling and testing methods. However, the grey literature obtained through this review played a key role in the contextual analysis and discussion section of this study. Certain limitations also include the use of only English, French and Spanish, as other publications in other languages may have relevant data not captured in these inclusion criteria. The study among MSWs from Côte d'Ivoire was included in the overall analysis; however, the sampling method directly recruited these individuals from an established sex worker clinic, and thus HIV prevalence may be overestimated in this subpopulation. Also, while the authors noted that the majority of MSWs in the Abidjan area were MSM, they did not collect data on types of partner [34]. The contextual description from the authors is supported by evidence from other contexts where partners of MSWs are male [108, 109]. Concurrently, systematic review methods were applied; however, sensitivity analysis and meta-analyses were not utilized. While odds ratios or aggregated comparison data were not generated, the overall analysis provides an overview of HIV prevalence among key populations and details of the epidemiology of key populations since the debut of HIV research in this region.

## Discussion

Epidemiologic literature over the past 30 years has demonstrated a consistent and disproportionate burden of HIV among key populations in WCA. From the first published study in 1987 to the most recent in 2013, elevated levels of HIV among FSWs and their clients were consistently reported. In recent years, studies emerged to display an elevated burden of HIV among MSM within the region, although the number of studies in this subpopulation remains limited. Concurrently, there is nascent but growing evidence of the existence of PWID and, consequently, HIV infections in this subpopulation [92].

### HIV prevalence

The elevated HIV prevalence among MSM, FSWs and clients of FSWs is important based on the determinants of the HIV epidemic in WCA and even more broadly across SSA. Surveillance has shown that women carry the highest burden of HIV on the continent, with national-level statistics constantly reporting that women have a higher HIV prevalence and incidence than men [13, 110]. While programmes are designed to address the various risks associated with female HIV acquisition, the results of this study demonstrate that HIV risks are significantly higher among FSWs than women who do not sell sex in WCA. These results are substantiated by a systematic review of FSWs in low and middle-income countries, which showed that FSWs in SSA have a pooled prevalence of 36.9% (95% CI 36.2–37.5) with a background prevalence on the continent of 7.42% in females [15]. Globally, FSWs were 13.5 (95% CI 10.0–18.1) times more likely to be living with HIV than women of reproductive age [15]. Thus, the results of this review and the epidemiology of HIV among FSWs worldwide suggest that inclusion of and significant focus on these women and their clients are of importance to address these populations’ high HIV acquisition and transmission risks in WCA [11, 72, 81, 84, 96].

On a continent where women are disproportionately burdened with HIV, prevalence of 17.7% (95% CI 16.5–18.9) among MSM demonstrates a potentially concentrated epidemic in this key population. A prevalence of 7.3% (95% CI 6.6–8.0) in clients of FSWs is also elevated compared to the general male population of the region and calls into question prevention programmes targeting this population. For clients of FSWs, male acquisition is linked to behavioural risks associated with multiple sexual partners, limited condom use and concomitant infection of an STI, amongst other determinants that are specific to men who engage in transactional sex [16, 74, 111]. For MSM, recent research has emerged that displays the increased transmission of HIV during anal sex, as well as sexual role versatility during same-sex practices that increases individual HIV risks and drives transmission within sexual networks of MSM [21]. Thus, the acknowledgement of a heightened burden of disease in these populations is important for the design and implementation of specialized HIV prevention, treatment and care programmes regionally [16, 26].

The heightened HIV prevalence in the MSM community found in these results is not unexpected, although the lack of data in WCA is noteworthy. The high prevalence reported in this review is comparable to other continents, with research indicating that MSM around the world are 19 times more likely to be infected with HIV than their adult male counterparts [18]. Interestingly, same-sex practices in WCA were reported as early as 1996 in a published population-based review [112]. The authors noted that the cumulative number of positive cases had exponentially increased from 1985 to 1995, and the primary modes of transmission were heterosexual practices (73.0%), homosexual practices (0.8%) and mother-to-child transmission (6.0%) [112]. More recent behavioural studies equally noted homosexual behaviour in different demographic studies. In Nigeria, 11.4% of sexually active secondary school students reported same-sex practices, and 12.4% reported anal sex [113]. In two Ghanaian studies in 2006 and 2008, prison inmates reported same-sex practices or identified as homosexual at 30.8% and 29.5%, respectively [114, 115]. While sporadic reports of same-sex practices and elevated HIV prevalence have been reported in the region, there is limited targeted programme activity for these men [5, 116, 117]. What does exist is limited in scale, based on community-driven initiatives, and functioning in highly stigmatized settings [33, 117, 118].

While HIV prevalence in PWID was found to be relatively low, the Nigerian study provides two important details for programming in WCA. Firstly, while it has generally been assumed that PWID constitute a minimal presence in WCA, the study's ability to generate a sample size of 1459 through respondent-driven sampling indicates that this population does exist. Secondly, while HIV prevalence appears low, we know from other contexts that once HIV is introduced into this specific subpopulation, the possibilities of rapid spread and sustained transmission are great [119, 120]. Contextually, policy makers are becoming aware of an increase of drug trafficking in the region, with large quantities of drugs confiscated in the past few years, and the recent conflict in Mali ascribed mainly to this trade [121]. Further supporting evidence of this regional trade was found in behavioural data in prisoners. In the same Ghanaian study in 2006, 41% of inmates reported imprisonment for narcotics; 7.3% had used cocaine, 5.2% heroin and 4.2% phencyclidine [114]. In the 2008 Ghanaian prison study, 35% of 1336 prisoners reported ever injecting drugs [115]. As was seen in Afghanistan as well as Thailand, Cambodia and other Southeast Asian countries, migration, trafficking, drug use and the HIV epidemic are intrinsically linked [119, 120, 122]. Thus, this is an important population to identify and appropriately engage in WCA in the coming decade of HIV prevention and control.

### Historical perspective

This review also indicates that knowledge of HIV prevalence among key populations and the proportion of HIV infections attributable to key populations in WCA are not representative of new or changing dynamics of HIV transmission. In 1995, Djomand *et al*. noted that the male:female ratio of HIV infection in Côte d'Ivoire had declined over time and the gender ratio had shown females to be 4.8 times more likely to be infected than men in 1988, compared to 1.9 times more likely in 1991 [20]. The authors asserted that this decline displayed that the HIV epidemic was initially concentrated in a core group of FSWs and their male partners, and was potentially expanding in broader populations with less identifiable risk factors, similar to dynamics observed in other regions outside of SSA [20, 122–124]. In 2004, Côté *et al*. conducted a study of adult males (15–59) in Accra, Ghana, and attributed 84% of existing cases of HIV to sex work and other transactional sex [125]. A study in 2008 based on Demographic and Health Surveys across four countries in SSA, including Ghana, showed that men who ever paid for sex were more likely to have HIV than men who had not (odds ratio 1.89, 95% CI 1.57–2.28) [126].

In the capital city of Lomé, Togo, researchers estimated the attributable fraction of current HIV cases to sex work and other transactional sex was 32%, in contrast to only 2% of cases outside of Lomé [18]. Finally, recently in Nigeria, a modes of transmission study asserted that 23% of HIV infection was attributable to key populations, including 10% of new infections amongst MSM [93]. Despite high HIV prevalence among key populations and a high number of HIV in 2009, cases attributable to behaviours such as sex between men and sex work, systematic prevention and treatment programmes for key populations have not been implemented regionally [5]. While prevention programmes for FSWs and their clients have been noted in countries including Ghana, Côte d'Ivoire, Nigeria and Cameroon, the appropriate scale of these programmes and collected surveillance data are limited, and HIV prevention, treatment and care programming for key populations has failed to become a standard of best practices in the region [5].

### Economic and regional migration

Underlying dynamics of the epidemic indicate external, economic and urban-centred disparities have contributed to the complexity of the HIV epidemic in WCA over time. Domestic and international migration patterns were repeatedly reported and significantly mirrored economic crises and fluctuations in specific countries. For example, a study in Côte d'Ivoire documenting the FSW population that accessed health clinics between 1991 and 1998 noted a major shift in country of origin over time, with Nigerian women surveyed increasing from 2 to 56% between 1992 and 1998, and Ghanaian women decreasing from 82 to 9% in the same time period [29]. Other studies reported the migration of Ghanaian FSWs to other countries in the 1990s and asserted that the significant economic and political crises in the country at that time contributed to this migration [3, 35]. The proportion of Liberian FSWs included in the same Ivorian study was shown to have increased from 0% in 1992 to 15% in 1995, and then to have declined to 2% in 1998 [94]. This evolution reflects the first internal conflict experienced in Liberia in the 1990s (1989–1996) [127, 128]. In a study reviewing the spread of HIV among FSWs in four cities across SSA, researchers noted that Cameroonian FSWs were more likely to have migrated internally to urban centres, while in Benin 86% of the FSWs sampled were from another country [41]. The only MSM study to discuss countries of origin was the MSW study in Côte d'Ivoire. Of the 96 MSWs sampled in Abidjan, 7.3% (7/96) reported a different country of origin [34].

The importance of these findings is revealed in the HIV prevalence among immigrants in the various studies. Nigerian and Ghanaian FSWs in the 2002 Côte d'Ivoire study were 1.03 (0.47–2.23) and 3.69 (2.28–5.97) times more likely to be infected than their counterparts from Côte d'Ivoire, Liberia and other West African countries [106]. In Lomé, two-thirds of FSWs were immigrants, and Ghanaian FSWs were 1.68 (1.06–2.66) times more likely to be living with HIV [126]. Addressing the needs of migrating populations at risk for or living with HIV is crucial, as these populations have less access to health services, are less likely to understand their human rights, and are more likely to contract a disease [129]. These populations are also more likely to be mobile; thus, successful prevention services for immigrant or mobile FSWs could potentially have an important impact in the overall reduction of HIV transmission and acquisition in the region [129].

Concurrently, disparity of HIV prevalence per locality was repeatedly reported in the various studies reviewed. In the same study that cited higher HIV levels among Ghanaian FSWs in Lomé, the prevalence among Lomé FSWs in 2005 was reported at 45.4% compared to 17.7% in the rest of Togo [18]. In two studies in Benin, there was significant spatial variation in the burden of HIV. For example, a study conducted in six cities in 2005 showed prevalence for HIV as high as 48.2% in Parakou, compared to 16.4% in Abomey/Bohicon [36]. A similar study found HIV prevalence in Cotonou, Benin, among FSWs to be 38.5%, compared to a pooled prevalence in three other large cities of the country of 58.9% [35]. Therefore, from an HIV prevention perspective, cross-border initiatives, effective community-based networking and standardized programmes across urban and regional landscapes for key populations are relevant for the WCA region.

#### Ways forward

Our review makes clear that there is a significant gap in the literature and subsequent HIV programmes for key populations in WCA. This may be ascribed to the application of the HIV response model of SSA to WCA epidemiological and prevention approaches. However, as reports of high HIV prevalence among key populations have existed in the literature since 1987, it also calls into question the structural barriers to healthcare for populations that engage in these defined sexual behaviours in this region. As in other contexts, sex work and other transactional sex, same-sex practice, and drug use are either criminalized or highly stigmatized in this region, and public policies have ignored or generally declined to address the specific health needs of key populations [5, 130, 131]. Research has shown that macro-level policies that impede or deter health service delivery for key populations ultimately increase vulnerability to disease acquisition [23, 130, 132].

Data presented here provide a useful framework for HIV programming in the region. The inclusion of relevant sexual history and behavioural questions in large-scale surveillance surveys, such as DHS, may also be of benefit in obtaining a better overview of the epidemiology of key populations, both in WCA and worldwide. While the delivery of sensitive questions such as engagement in sex work, transactional sex, same-sex practices and drug use must be carefully administered (ideally not within the household setting), standardized national data collection would go far to inform country and regional policy development in WCA.

Subsequently, emerging data have shown that addressing the epidemic in key populations requires combined behavioural, biomedical and structural approaches [23, 133]. Limited condom use with regular sexual partners, unawareness of HIV status and co-infections with genital ulcerative diseases are contributing factors to heightened prevalence [10, 21, 116]. High prevalence among key populations concurrently has implications for prioritized biomedical interventions [21, 134].

While the knowledge that these populations have a higher risk for transmission and acquisition of HIV and other STIs is acknowledged, the method in which prevention and treatment programmes address these risks has yet to be firmly cemented in HIV prevention programming [13]. Researchers in the United States and elsewhere have demonstrated the importance of engaging populations in the continuum of HIV care – from undiagnosed cases to testing and diagnosis, followed by linkage to ongoing care and treatment [135]. The continuum of HIV care significantly reduces the viral load among people living with HIV and ultimately reduces transmission [135, 136]. In two recent studies in the United States, researchers found that due to advances in antiretroviral regimes, with 70–80% adherence to antiretroviral therapy (ART) by participants, durable viral suppression occurred in most individuals, lowering the possibility for onward HIV transmission [136, 137]. The findings indicate that the key to community viral suppression is early diagnosis of the disease, well-developed referral systems to clinical services, and care and support programmes that encourage adherence and access to treatment [136]. This approach has been shown to be effective in contexts with both high and low prevalence, and recent research from South Africa affirms that adequate ART coverage at the community level reduces incidence over time [138]. Thus, prevention programmes are beginning to show that distribution of prevention commodities and messages should be in concert with interventions that address the virology and biomedical aspects of care and treatment [135]. This is even more relevant for key populations who carry a significant burden of disease and ultimately are people living with HIV.

Structural factors acting at the macro- and meso-levels should not be ignored in WCA and are essential when building combination biomedical programmes [23, 139]. Criminalization and public policy neglect substantially inhibit key populations’ ability to access appropriate, life-sustaining and prevention-oriented health services. Policy-level gaps and community-level stigma must be addressed if programmes are to adequately confront the needs of these populations [140, 141]. Studies from other countries on the continent indicate the stigma experienced within their communities and at health services, significantly deters the uptake at clinical services for key populations [130, 142]. Public policies that adequately address the intricate health needs, reduce stigma and discrimination, and facilitate community and provider level HIV care and treatment delivery will highly benefit the overall control and prevention of HIV among key populations in WCA [23].

## Conclusions

This systematic review suggests that the concentrated HIV epidemic in WCA more closely resembles the epidemics in Southeast Asia and Latin America than those in the rest of SSA. This not only calls into question the response to the HIV epidemic in WCA but indicates that the region has an opportunity to adapt and develop region-specific prevention and treatment strategies. Targeted, cost-effective programmes that address not only behavioural but also biological and structural risk factors associated with HIV acquisition and transmission key populations should be engaged to reduce the onward spread of HIV. Prevention programmes should model strategies on appropriate programmes that reduce community viral loads, increase uptake of treatment among key populations and address the barriers to healthcare that exist in highly stigmatized settings. Ensuring that programmes rooted in community-based approaches address the continuum of HIV care, from diagnosis to viral suppression, will be a challenge but also a possible victory for HIV prevention and control in WCA.

## References

[1] Bank TW (2012). Population estimates 2008–2012. GPE Open Data Project 2012.

[2] Bank TW (2013). Economic assessment of lending groups, world atlas method. GPE Open Data Project.

[3] Agyei-Mensah S (2001). Twelve years of HIV/AIDS in Ghana: puzzles of interpretation. Can J Afr Stud.

[4] Gisselquist D (2003). Emergence of the HIV type 1 epidemic in the twentieth century: comparing hypotheses to evidence. AIDS Res Hum Retroviruses.

[5] UNAIDS (2008). West and Central Africa: towards universal access to prevention, care and treatment.

[6] UNAIDS (2013). Report on the global AIDS epidemic.

[7] Wilson D, Halperin DT (2008). “Know your epidemic, know your response”: a useful approach, if we get it right. Lancet.

[8] UNAIDS Practical guidelines for intensifying HIV prevention: towards universal access [Internet]. http://dataunaidsorg/pub/Manual/2007/20070306_2007.;Prevention_Guidelines_Towards_Universal_Access_en.pdf.

[9] Unaids W (2000). Guidelines for second generation HIV surveillance.

[10] Espirito Santo ME, Etheredge GD (2002). How to reach clients of female sex workers: a survey by surprise in brothels in Dakar, Senegal. Bull World Health Organ.

[11] Unaids (2008). Sub-Saharan Africa: AIDS epidemic update regional summary.

[12] Lowndes CM, Alary M, Belleau M, Kofi Bosu W, Federic Kintin D, Asonye Nnorom (2008). West Africa HIV/AIDS epidemiology and response synthesis: characterisation of the HIV epidemic and reponse in West Africa: implications for prevention.

[13] Baral S, Phaswana-Mafuya N (2012). Rewriting the narrative of the epidemiology of HIV in sub-Saharan Africa. SAHARA J.

[14] Drame FM, Peitzmeier S, Lopes M, Ndaw M, Sow A, Diouf D (2013). Gay men and other men who have sex with men in West Africa: evidence from the field. Cult Health Sex.

[15] Baral S, Beyrer C, Muessig K, Poteat T, Wirtz AL, Decker MR (2012). Burden of HIV among female sex workers in low-income and middle-income countries: a systematic review and meta-analysis. Lancet Infect Dis.

[16] Lowndes CM, Alary M, Meda H, Gnintoungbe CA, Mukenge-Tshibaka L, Adjovi C (2002). Role of core and bridging groups in the transmission dynamics of HIV and STIs in Cotonou, Benin, West Africa. Sex Transm Infect.

[17] Djomand G, Greenberg AE, Sassan-Morokro M, Tossou O, Diallo MO, Ekpini E (1995). The epidemic of HIV/AIDS in Abidjan, Cote d'Ivoire: a review of data collected by Projet RETRO-CI from 1987 to 1993. J Acquir Immune Defic Syndr Hum Retrovirol.

[18] Sobela F, Pepin J, Gbeleou S, Banla AK, Pitche VP, Adom W (2009). A tale of two countries: HIV among core groups in Togo. J Acquir Immune Defic Syndr.

[19] Scott Kellerman M, Holtz S, Dutta A, Aliou S, Diallo I, Redding S (2012). The epidemiology of HIV epidemics in the 21-country West and Central Africa region: the impact of most at risk populations (MARPs). USAID AWARE II project.

[20] Baral S, Sifakis F, Cleghorn F, Beyrer C (2007). Elevated risk for HIV infection among men who have sex with men in low- and middle-income countries 2000–2006: a systematic review. PLoS Med.

[21] Beyrer C, Baral SD, van Griensven F, Goodreau SM, Chariyalertsak S, Wirtz AL (2012). Global epidemiology of HIV infection in men who have sex with men. Lancet.

[22] Mathers BM, Degenhardt L, Ali H, Wiessing L, Hickman M, Mattick R (2010). HIV prevention, treatment and care for people who inject drugs: a systematic review of global, regional and country level coverage. Lancet.

[23] Baral S, Logie CH, Grosso A, Wirtz A, Beyrer C (2013). Modified social ecological model: a tool to guide the assessment of the risks and risk contexts of HIV epidemics. BMC Public Health.

[24] Nelson KE, Celentano DD, Eiumtrakol S, Hoover DR, Beyrer C, Suprasert S (1996). Changes in sexual behavior and a decline in HIV infection among young men in Thailand. N Engl J Med.

[25] UNAIDS (2012). Regional response fact sheet: Asia and the Pacific.

[26] UNAIDS (2012). Regional fact sheet: Latin America and the Caribbean.

[27] UNAIDS (2010). Report on the global AIDS epidemic 2010.

[28] Hammett TM, Kling R, Johnston P, Liu W, Ngu D, Friedmann P (2006). Patterns of HIV prevalence and HIV risk behaviors among injection drug users prior to and 24 months following implementation of cross-border HIV prevention interventions in northern Vietnam and southern China. AIDS Educ Prev.

[29] Ghys PD, Diallo MO, Ettiegne-Traore V, Kale K, Tawil O, Carael M (2002). Increase in condom use and decline in HIV and sexually transmitted diseases among female sex workers in Abidjan, Cote d'Ivoire, 1991–1998. AIDS.

[30] Liberati A, Altman DG, Tetzlaff J, Mulrow C, Gøtzsche PC, Ioannidis JPA (2009). The PRISMA statement for reporting systematic reviews and meta-analyses of studies that evaluate health care interventions: explanation and elaboration. J Clin Epidemiol.

[31] Lipsey MW, Wilson DB (2001). Practical meta-analysis. Applied Social Research Methods Series, Volume 49.

[32] Mateen FJ, Oh J, Tergas AI, Bhayani NH, Kamdar BB (2013). Titles versus titles and abstracts for initial screening of articles for systematic reviews. Clin Epidemiol.

[33] Beyrer C (2012). Lesbian, gay, bisexual, and transgender populations in Africa: a social justice movement emerges in the era of HIV. SAHARA J.

[34] Vuylsteke B, Semde G, Sika L, Crucitti T, Ettiegne Traore V, Buve A (2012). High prevalence of HIV and sexually transmitted infections among male sex workers in Abidjan, Cote d'Ivoire: need for services tailored to their needs. Sex Transm Infect.

[35] Ahoyo AB, Alary M, Meda H, Ndour M, Batona G, Bitera R (2007). Female sex workers in Benin, 2002. Behavioural survey and HIV and other STI screening. Sante.

[36] Ahoyo AB, Alary M, Ndour M, Labbe AC, Ahoussinou C (2009). HIV and sexually transmitted disease among female sex workers in Benin. Med Trop.

[37] Alary M, Mukenge-Tshibaka L, Bernier F, Geraldo N, Lowndes CM, Meda H (2002). Decline in the prevalence of HIV and sexually transmitted diseases among female sex workers in Cotonou, Benin, 1993–1999. AIDS.

[38] Baganizi E, Alary M, Guedeme A, Padonou F, Davo N, Adjovi C (1997). HIV infection in female prostitutes from Benin: association with symptomatic but not asymptomatic gonococcal or chlamydial infections. AIDS.

[39] Bigot A, Bodeus M, Burtonboy G, Ahouignan G, Zohoun I (1992). Prevalence of HIV infection among prostitutes in Benin (West Africa). J Acquir Immune Defic Syndr.

[40] Labbe AC, Pepin J, Khonde N, Dzokoto A, Meda H, Asamoah-Adu C (2012). Periodical antibiotic treatment for the control of gonococcal and chlamydial infections among sex workers in Benin and Ghana: a cluster-randomized placebo-controlled trial. Sex Transm Dis.

[41] Morison L, Weiss HA, Buve A, Carael M, Abega SC, Kaona F (2001). Commercial sex and the spread of HIV in four cities in sub-Saharan Africa. AIDS.

[42] Lowndes CM, Alary M, Gnintoungbe CA, Bedard E, Mukenge L, Geraldo N (2000). Management of sexually transmitted diseases and HIV prevention in men at high risk: targeting clients and non-paying sexual partners of female sex workers in Benin. AIDS.

[43] Lowndes CM, Alary M, Labbe AC, Gnintoungbe C, Belleau M, Mukenge L (2007). Interventions among male clients of female sex workers in Benin, West Africa: an essential component of targeted HIV preventive interventions. Sex Transm Infect.

[44] Lankoande S, Meda N, Sangare L, Compaore IP, Catraye J, Sanou PT (1998). Prevalence and risk of HIV infection among female sex workers in Burkina Faso. Int J STD AIDS.

[45] Nagot N, Ouangre A, Ouedraogo A, Cartoux M, Huygens P, Defer MC (2002). Spectrum of commercial sex activity in Burkina Faso: classification model and risk of exposure to HIV. J Acquir Immune Defic Syndr.

[46] Kaptue L, Zekeng L, Djoumessi S, Monny-Lobe M, Nichols D, Debuysscher R (1991). HIV and chlamydia infections among prostitutes in Yaounde, Cameroon. Genitourin Med.

[47] Mauclere P, Mahieux R, Garcia-Calleja JM, Salla R, Tekaia F, Millan J (1995). A new HTLV type II subtype A isolate in an HIV type 1-infected prostitute from Cameroon, Central Africa. AIDS Res Hum Retroviruses.

[48] Ryan KA, Roddy RE, Zekeng L, Weir SS, Tamoufe U (1998). Characteristics associated with prevalent HIV infection among a cohort of sex workers in Cameroon. Sex Transm Infect.

[49] Bestetti G, Renon G, Mauclere P, Ruffie A, Mbopi Keou FX, Eme D (1998). High seroprevalence of human herpesvirus-8 in pregnant women and prostitutes from Cameroon. AIDS.

[50] Mosoko JJ, Macauley IB, Zoungkanyi AC, Bella A, Koulla-Shiro S (2009). Human immunodeficiency virus infection and associated factors among specific population subgroups in Cameroon. AIDS Behav.

[51] Mann JM, Nzilambi N, Piot P, Bosenge N, Kalala M, Francis H (1988). HIV infection and associated risk factors in female prostitutes in Kinshasa, Zaire. AIDS.

[52] Mulanga-Kabeya C, Nzilambi N, Edidi B, Minlangu M, Tshimpaka T, Kambembo L (1998). Evidence of stable HIV seroprevalences in selected populations in the Democratic Republic of the Congo. AIDS.

[53] Nzila N, Laga M, Thiam MA, Mayimona K, Edidi B, Van Dyck E (1991). HIV and other sexually transmitted diseases among female prostitutes in Kinshasa. AIDS.

[54] Nzilambi N, De Cock KM, Forthal DN, Francis H, Ryder RW, Malebe I (1988). The prevalence of infection with human immunodeficiency virus over a 10-year period in rural Zaire. N Engl J Med.

[55] Vandepitte JM, Malele F, Kivuvu DM, Edidi S, Muwonga J, Lepira F (2007). HIV and other sexually transmitted infections among female sex workers in Kinshasa, Democratic Republic of Congo, in 2002. Sex Transm Dis.

[56] Pepin J, Morgan G, Dunn D, Gevao S, Mendy M, Gaye I (1991). HIV-2-induced immunosuppression among asymptomatic West African prostitutes: evidence that HIV-2 is pathogenic, but less so than HIV-1. AIDS.

[57] Pepin J, Dunn D, Gaye I, Alonso P, Egboga A, Tedder R (1991). HIV-2 infection among prostitutes working in The Gambia: association with serological evidence of genital ulcer diseases and with generalized lymphadenopathy. AIDS.

[58] Pickering H, Quigley M, Pepin J, Todd J, Wilkins A (1993). The effects of post-test counselling on condom use among prostitutes in The Gambia. AIDS.

[59] Pickering H, Todd J, Dunn D, Pepin J, Wilkins A (1992). Prostitutes and their clients: a Gambian survey. Soc Sci Med.

[60] Asamoah-Adu C, Khonde N, Avorkliah M, Bekoe V, Alary M, Mondor M (2001). HIV infection among sex workers in Accra: need to target new recruits entering the trade. J Acquir Immune Defic Syndr.

[61] Deceuninck G, Asamoah-Adu C, Khonde N, Pepin J, Frost EH, Deslandes S (2000). Improvement of clinical algorithms for the diagnosis of *Neisseria gonorrhoeae* and *Chlamydia trachomatis* by the use of Gram-stained smears among female sex workers in Accra, Ghana. Sex Transm Dis.

[62] Côté AM, Sobela F, Dzokoto A, Nzambi K, Asamoah-Adu C, Labbe AC (2004). Transactional sex is the driving force in the dynamics of HIV in Accra, Ghana. AIDS.

[63] Aho J, Koushik A, Diakite SL, Loua KM, Nguyen VK, Rashed S (2010). Biological validation of self-reported condom use among sex workers in Guinea. AIDS Behav.

[64] Aho J, Nguyen VK, Diakite S, Sow A, Koushik A, Rashed S (2012). High acceptability of HIV voluntary counselling and testing among female sex workers: impact of individual and social factors. HIV Med.

[65] Diallo BL, Alary M, Rashed S, Barry A (2011). HIV prevalence, associated risk factors and evolution among truck drivers from 2001 to 2007 in Guinea. Med Trop.

[66] Denis F, Barin F, Gershy-Damet G, Rey JL, Lhuillier M, Mounier M (1987). Prevalence of human T-lymphotropic retroviruses type III (HIV) and type IV in Ivory Coast. Lancet.

[67] Ettiegne-Traore V, Ghys PD, Maurice C, Hoyi-Adonsou YM, Soroh D, Adom ML (1998). Evaluation of an HIV saliva test for the detection of HIV-1 and HIV-2 antibodies in high-risk populations in Abidjan, Cote d'Ivoire. Int J STD AIDS.

[68] Ghys PD, Diallo MO, Ettiegne-Traore V, Yeboue KM, Gnaore E, Lorougnon F (1995). Dual seroreactivity to HIV-1 and HIV-2 in female sex workers in Abidjan, Cote d'Ivoire. AIDS.

[69] Ghys PD, Fransen K, Diallo MO, Ettiegne-Traore V, Coulibaly IM, Yeboue KM (1997). The associations between cervicovaginal HIV shedding, sexually transmitted diseases and immunosuppression in female sex workers in Abidjan, Cote d'Ivoire. AIDS.

[70] Koffi K, Gershy-Damet GM, Peeters M, Soro B, Rey JL, Delaporte E (1992). Rapid spread of HIV infections in Abidjan, Ivory Coast, 1987–1990. Eur J Clin Microbiol Infect Dis.

[71] Nkengasong JN, Kestens L, Ghys PD, Koblavi-Deme S, Otten RA, Bile C (2000). Dual infection with human immunodeficiency virus type 1 and type 2: impact on HIV type 1 viral load and immune activation markers in HIV-seropositive female sex workers in Abidjan, Ivory Coast. AIDS Res Hum Retroviruses.

[72] Vuylsteke B, Semde G, Sika L, Crucitti T, Ettiegne Traore V, Buve A (2012). HIV and STI prevalence among female sex workers in Cote d'Ivoire: why targeted prevention programs should be continued and strengthened. PLoS One.

[73] Ouattara SA, Diallo D, Meite M, Aron Y, Akran V, Gody M (1988). Epidemiology of infections caused by human immunodeficiency viruses HIV-1 and HIV-2 in the Ivory Coast. Med Trop.

[74] Vuylsteke BL, Ghys PD, Traore M, Konan Y, Mah-Bi G, Maurice C (2003). HIV prevalence and risk behavior among clients of female sex workers in Abidjan, Cote d'Ivoire. AIDS.

[75] Peeters M, Koumare B, Mulanga C, Brengues C, Mounirou B, Bougoudogo F (1998). Genetic subtypes of HIV type 1 and HIV type 2 strains in commercial sex workers from Bamako, Mali. AIDS Res Hum Retroviruses.

[76] Pichard E, Guindo A, Grossetete G, Fofana Y, Maiga YI, Koumare B (1988). Human immunodeficiency virus (HIV) infection in Mali. Med Trop.

[77] Gragnic G, Julvez J, Abari A, Alexandre Y (1998). HIV-1 and HIV-2 seropositivity among female sex workers in the Tenere Desert, Niger. Trans R Soc Trop Med Hyg.

[78] Mamadou S, Laouel Kader A, Rabiou S, Aboubacar A, Soumana O, Garba A (2006). Prevalence of the HIV infection and five other sexually-transmitted infections among sex workers in Niamey, Niger. Bull Soc Pathol Exot.

[79] Develoux M, Meynard D, Dupont A, Delaporte E (1994). Hepatitis C virus antibodies in prostitutes in Niger. Trans R Soc Trop Med Hyg.

[80] Tohon Z, Garba A, Amadou Hamidou A, Sidikou F, Ibrahim ML, Elhadj Mahamane A (2006). Behaviour and HIV seroprevalence investigation in sex workers of Dirkou, Niger, 2002. Bull Soc Pathol Exot.

[81] Ahmed S, Delaney K, Villalba-Diebold P, Aliyu G, Constantine N, Ememabelem M (2013). HIV counseling and testing and access-to-care needs of populations most-at-risk for HIV in Nigeria. AIDS Care.

[82] Dada AJ, Ajayi AO, Diamondstone L, Quinn TC, Blattner WA, Biggar RJ (1998). A serosurvey of Haemophilus ducreyi, syphilis, and herpes simplex virus type 2 and their association with human immunodeficiency virus among female sex workers in Lagos, Nigeria. Sex Transm Dis.

[83] Dada AJ, Oyewole F, Onofowokan R, Nasidi A, Harris B, Levin A (1993). Demographic characteristics of retroviral infections (HIV-1, HIV-2, and HTLV-I) among female professional sex workers in Lagos, Nigeria. J Acquir Immune Defic Syndr.

[84] Eluwa GI, Strathdee SA, Adebajo SB, Ahonsi B, Azeez A, Anyanti J (2012). Sexual risk behaviors and HIV among female sex workers in Nigeria. J Acquir Immune Defic Syndr.

[85] Imade G, Sagay A, Egah D, Onwuliri V, Grigg M, Egbodo C (2008). Prevalence of HIV and other sexually transmissible infections in relation to lemon or lime juice douching among female sex workers in Jos, Nigeria. Sex Health.

[86] Lawan UM, Abubakar S, Ahmed A (2012). Risk perceptions, prevention and treatment seeking for sexually transmitted infections and HIV/AIDS among female sex workers in Kano, Nigeria. Afr J Reprod Health.

[87] Obi CL, Ogbonna BA, Igumbor EO, Ndip RN, Ajayi AO (1993). HIV seropositivity among female prostitutes and nonprostitutes: obstetric and perinatal implications. Viral Immunol.

[88] Olaleye OD, Bernstein L, Ekweozor CC, Sheng Z, Omilabu SA, Li XY (1993). Prevalence of human immunodeficiency virus types 1 and 2 infections in Nigeria. J Infect Dis.

[89] Bakare RA, Oni AA, Umar US, Adewole IF, Shokunbi WA, Fayemiwo SA (2002). Pattern of sexually transmitted diseases among commercial sex workers (CSWs) in Ibadan, Nigeria. Afr J Med Sci.

[90] Chikwem JO, Mohammed I, Ola T (1989). Human immunodeficiency virus type 1 (HIV-1) infection among female prostitutes in Borno State of Nigeria: one year follow-up. East Afr Med J.

[91] Fayemiwo SA, Odaibo GN, Oni AA, Ajayi AA, Bakare RA, Olaleye DO (2011). Genital ulcer diseases among HIV-infected female commercial sex workers in Ibadan, Nigeria. Afr J Med Sci.

[92] Eluwa GI, Strathdee SA, Adebayo SB, Ahonsi B, Adebajo SB (2013). A profile on HIV prevalence and risk behaviors among injecting drug users in Nigeria: should we be alarmed?. Drug Alcohol Depend.

[93] Adebajo SB, Eluwa GI, Allman D, Myers T, Ahonsi BA (2012). Prevalence of internalized homophobia and HIV associated risks among men who have sex with men in Nigeria. Afr J Reprod Health.

[94] Merrigan M, Azeez A, Afolabi B, Chabikuli ON, Onyekwena O, Eluwa G (2011). HIV prevalence and risk behaviours among men having sex with men in Nigeria. Sex Transm Infect.

[95] Vu L, Adebajo S, Tun W, Sheehy M, Karlyn A, Njab J (2013). High HIV prevalence among men who have sex with men in Nigeria: implications for combination prevention. J Acquir Immune Defic Syndr.

[96] Kane CT, Diawara S, Ndiaye HD, Diallo PA, Wade AS, Diallo AG (2009). Concentrated and linked epidemics of both HSV-2 and HIV-1/HIV-2 infections in Senegal: public health impacts of the spread of HIV. Int J STD AIDS.

[97] Kanki P, M'Boup S, Marlink R, Travers K, Hsieh CC, Gueye A (1992). Prevalence and risk determinants of human immunodeficiency virus type 2 (HIV-2) and human immunodeficiency virus type 1 (HIV-1) in west African female prostitutes. Am J Epidemiol.

[98] Langley CL, Benga-De E, Critchlow CW, Ndoye I, Mbengue-Ly MD, Kuypers J (1996). HIV-1, HIV-2, human papillomavirus infection and cervical neoplasia in high-risk African women. AIDS.

[99] Laurent C, Seck K, Coumba N, Kane T, Samb N, Wade A (2003). Prevalence of HIV and other sexually transmitted infections, and risk behaviours in unregistered sex workers in Dakar, Senegal. AIDS.

[100] Ndiaye CF, Critchlow CW, Leggott PJ, Kiviat NB, Ndoye I, Robertson PB (1997). Periodontal status of HIV-1 and HIV-2 seropositive and HIV seronegative female commercial sex workers in Senegal. J Periodontol.

[101] Wang C, Hawes SE, Gaye A, Sow PS, Ndoye I, Manhart LE (2007). HIV prevalence, previous HIV testing, and condom use with clients and regular partners among Senegalese commercial sex workers. Sex Transm Infect.

[102] Thior I, Diouf G, Diaw IK, Sarr AD, Hsieh CC, Ndoye I (1997). Sexually transmitted diseases and risk of HIV infection in men attending a sexually transmitted diseases clinic in Dakar, Senegal. Afr J Reprod Health.

[103] do Espirito Santo ME, Etheredge GD (2003). HIV prevalence and sexual behaviour of male clients of brothels’ prostitutes in Dakar, Senegal. AIDS Care.

[104] Wade AS, Larmarange J, Diop AK, Diop O, Gueye K, Marra A (2010). Reduction in risk-taking behaviors among MSM in Senegal between 2004 and 2007 and prevalence of HIV and other STIs. ELIHoS Project, ANRS 12139. AIDS Care.

[105] Wade AS, Kane CT, Diallo PA, Diop AK, Gueye K, Mboup S (2005). HIV infection and sexually transmitted infections among men who have sex with men in Senegal. AIDS.

[106] Stromdahl S, Onigbanjo Williams A, Eziefule B, Emmanuel G, Iwuagwu S, Anene O (2012). Associations of consistent condom use among men who have sex with men in Abuja, Nigeria. AIDS Res Hum Retroviruses.

[107] Ndiaye HD, Toure-Kane C, Vidal N, Niama FR, Niang-Diallo PA, Dieye T (2009). Surprisingly high prevalence of subtype C and specific HIV-1 subtype/CRF distribution in men having sex with men in Senegal. J Acquir Immune Defic Syndr.

[108] Baral S, Kizub D, Masenior NF, Peryskina A, Stachowiak J, Stibich M (2010). Male sex workers in Moscow, Russia: a pilot study of demographics, substance use patterns, and prevalence of HIV-1 and sexually transmitted infections. AIDS Care.

[109] Infante C, Sosa-Rubi SG, Cuadra SM (2009). Sex work in Mexico: vulnerability of male, travesti, transgender and transsexual sex workers. Cult Health Sex.

[110] Ngugi E (2012). Female sex workers in Africa: epidemiology overview, data gaps, ways forward. SAHARA J.

[111] Lawoyin TO (2004). Condom use with sex workers and abstinence behaviour among men in Nigeria. J R Soc Promot Health.

[112] Fourn L, Ducic S (1996). Epidemiological portrait of acquired immunodeficiency syndrome and its implications in Benin. Sante (Montrouge France).

[113] Bamidele JO, Abodunrin OL, Adebimpe WO (2009). Sexual behavior and risk of HIV/AIDS among adolescents in public secondary schools in Osogbo, Osun State, Nigeria. Int J Adolesc Med Health.

[114] Adjei AA, Armah HB, Gbagbo F, Ampofo WK, Quaye IK, Hesse IF (2006). Prevalence of human immunodeficiency virus, hepatitis B virus, hepatitis C virus and syphilis among prison inmates and officers at Nsawam and Accra, Ghana. J Med Microbiol.

[115] Adjei AA, Armah HB, Gbagbo F, Ampofo WK, Boamah I, Adu-Gyamfi C (2008). Correlates of HIV, HBV, HCV and syphilis infections among prison inmates and officers in Ghana: a national multicenter study. BMC Infect Dis.

[116] Alary M, Lowndes CM, Mukenge-Tshibaka L, Gnintoungbe CA, Bedard E, Geraldo N (2003). Sexually transmitted infections in male clients of female sex workers in Benin: risk factors and reassessment of the leucocyte esterase dipstick for screening of urethral infections. Sex Transm Infect.

[117] Henry E, Marcellin F, Yomb Y, Fugon L, Nemande S, Gueboguo C (2010). Factors associated with unprotected anal intercourse among men who have sex with men in Douala, Cameroon. Sex Transm Infect.

[118] Kalamar M, Maharaj P, Gresh A (2011). HIV-prevention interventions targeting men having sex with men in Africa: field experiences from Cameroon. Cult Health Sex.

[119] Beyrer C, Jittiwutikarn J, Teokul W, Razak MH, Suriyanon V, Srirak N (2003). Drug use, increasing incarceration rates, and prison-associated HIV risks in Thailand. AIDS Behav.

[120] Beyrer C, Mehta SH, Baral SD, Pizer H, Mayer K (2006). The international drug epidemic. Social ecology of infectious disease.

[121] Perry A (2012). The cocaine crisis: how the drug trade is ruining West Africa. Time Magazine.

[122] Beyrer C (1998). War in the blood. Sex, politics and HIV/AIDS in SouthEast Asia.

[123] Beyrer C (2002). Fearful symmetry: heroin trafficking and HIV spread. Bull Narc.

[124] Mathers BM, Degenhardt L, Adam P, Toskin I, Nashkhoev M, Lyerla R (2009). Estimating the level of HIV prevention coverage, knowledge and protective behavior among injecting drug users: what does the 2008 UNGASS reporting round tell us?. J Acquir Immune Defic Syndr.

[125] Cote AM, Sobela F, Dzokoto A, Nzambi K, samoah-Adu C, Labbe AC (2004). Transactional sex is the driving force in the dynamics of HIV in Accra, Ghana. AIDS.

[126] Leclerc PM, Garenne M (2008). Commercial sex and HIV transmission in mature epidemics: a study of five African countries. Int J STD AIDS.

[127] Decosas J, Kane F, Anarfi JK, Sodji KD, Wagner HU (1995). Migration and AIDS. Lancet.

[128] Meredith M (2006). The state of Africa: a history of fifty years of independence.

[129] Beyrer C, Baral S, Zenilman J, Holmes KK, Sparling PF, Mardh PA, Lemon SM, Stamm WE, Piot P (2006). STDs, HIV/AIDS and migrant populations.

[130] Poteat TD, Diouf D, Baral S, Ndaw M, Drame F, Traore C, Wirtz A The impact of criminalization of same sex practices on HIV risk among men who have sex with men (MSM) in Senegal: results of a qualitative rapid assessment (TUPE0709).

[131] Munoz J, Adedimeji A, Alawode O (2010). ‘They bring AIDS to us and say we give it to them’: socio-structural context of female sex workers’ vulnerability to HIV infection in Ibadan, Nigeria. SAHARA J.

[132] Millett GA, Flores SA, Peterson JL, Bakeman R (2007). Explaining disparities in HIV infection among black and white men who have sex with men: a meta-analysis of HIV risk behaviors. AIDS.

[133] Baral SD, Wirtz A, Sifakis F, Johns B, Walker D, Beyrer C (2012). The highest attainable standard of evidence (HASTE) for HIV/AIDS interventions: toward a public health approach to defining evidence. Public Health Rep.

[134] Dreazen Y

[135] Beyrer C, Baral S, Kerrigan D, El-Bassel N, Bekker LG, Celentano DD (2011). Expanding the space: inclusion of most-at-risk populations in HIV prevention, treatment, and care services. J Acquir Immune Defic Syndr.

[136] Gardner EM, McLees MP, Steiner JF, Del Rio C, Burman WJ (2011). The spectrum of engagement in HIV care and its relevance to test-and-treat strategies for prevention of HIV infection. Clin Infect Dis.

[137] Gross R, Yip B, Lo Re V, Wood E, Alexander CS, Harrigan PR (2006). A simple, dynamic measure of antiretroviral therapy adherence predicts failure to maintain HIV-1 suppression. J Infect Dis.

[138] Tanser F, Barnighausen T, Grapsa E, Zaidi J, Newell ML (2013). High coverage of ART associated with decline in risk of HIV acquisition in rural KwaZulu-Natal, South Africa. Science.

[139] Auerbach JD, Parkhurst JO, Cáceres CF (2011). Addressing social drivers of HIV/AIDS for the long-term response: conceptual and methodological considerations. Glob Public Health.

[140] Logie C (2012). The case for the World Health Organization's Commission on the Social Determinants of Health to address sexual orientation. Am J Public Health.

[141] Chakrapani V, Newman PA, Shunmugam M, Kurian AK, Dubrow R (2009). Barriers to free antiretroviral treatment access for female sex workers in Chennai, India. AIDS Patient Care and STDs.

[142] Baral S, Semugoma P, Diouf D, Trapence G, Poteat T, Ndaw M, Drame F Criminalization of same sex practices as a structural driver of HIV risk among men who have sex with men (MSM): the cases of Senegal, Malawi, and Uganda (MOPE0951).

# Sub article: HIV prevalence and factors associated with HIV infection among men who have sex with men in Cameroon

**DOI:** 10.7448/IAS.16.4.18752

**Published:** 2013-12-02

**Authors:** Ju Nyeong Park, Erin Papworth, Sethson Kassegne, Laure Moukam, Serge Clotaire Billong, Issac Macauley, Yves Roger Yomb, Nathalie Nkoume, Valentin Mondoleba, Jules Eloundou, Matthew LeBreton, Ubald Tamoufe, Ashley Grosso, Stefan D Baral

**Affiliations:** 1 Center for Public Health and Human Rights, Department of Epidemiology, Johns Hopkins Bloomberg School of Public Health, Baltimore, MD, USA; 2 West and Central Africa Region, Population Services International, Cotonou, Benin; 3 Association Camerounaise pour le Marketing Social (ACMS), Yaoundé, Cameroon; 4 Comité national de lutte contre le sida (CNLS), Ministère de la Sante Publique (MINSANTE), Yaoundé, Cameroon; 5 CARE International-Cameroon, Yaoundé, Cameroon; 6 Alternatives-Cameroun, Douala, Cameroon; 7 CAMNAFAW, Yaoundé, Cameroon; 8 Humanity First, Yaoundé, Cameroon; 9 Global Viral Cameroon, Yaoundé, Cameroon

**Keywords:** Men who have sex with men (MSM), HIV/AIDS, epidemiology, Africa, prevalence, respondent-driven sampling (RDS), homosexuality, prevention, risk factors, sexual behaviour

## Abstract

**Introduction:**

Despite men who have sex with men (MSM) being a key population for HIV programming globally, HIV epidemiologic data on MSM in Central Africa are sparse. We measured HIV and syphilis prevalence and the factors associated with HIV infection among MSM in Cameroon.

**Methods:**

Two hundred and seventy-two and 239 MSM aged ≥18 from Douala and Yaoundé, respectively, were recruited using respondent-driven sampling (RDS) for this cross-sectional surveillance study in 2011. Participants completed a structured questionnaire and HIV and syphilis testing. Statistical analyses, including RDS-weighted proportions, bootstrapped confidence intervals and logistic regressions, were used.

**Results:**

Crude and RDS-weighted HIV prevalence were 28.6% (73/255) and 25.5% (95% CI 19.1–31.9) in Douala, and 47.3% (98/207) and 44.4% (95% CI 35.7–53.2) in Yaoundé. Active syphilis prevalence in total was 0.4% (2/511). Overall, median age was 24 years, 62% (317/511) of MSM identified as bisexual and 28.6% (144/511) identified as gay. Inconsistent condom use with regular male partners (64.1%; 273/426) and casual male and female partners (48.5%; 195/402) was common, as was the inconsistent use of condom-compatible lubricants (CCLs) (26.3%; 124/472). In Douala, preferring a receptive sexual role was associated with prevalent HIV infection [adjusted odds ratio (aOR) 2.33, 95% CI 1.02–5.32]. Compared to MSM without HIV infection, MSM living with HIV were more likely to have ever accessed a health service targeting MSM in Douala (aOR 4.88, 95% CI 1.63–14.63). In Yaoundé, MSM living with HIV were more likely to use CCLs (aOR 2.44, 95% CI 1.19–4.97).

**Conclusions:**

High HIV prevalence were observed and condoms and CCLs were used inconsistently indicating that MSM are a priority population for HIV prevention, treatment and care services in Douala and Yaoundé. Building the capacity of MSM community organizations and improving the delivery and scale-up of multimodal interventions for MSM that are sensitive to concerns about confidentiality and the complex individual, social, community-level and policy challenges are needed to successfully engage young MSM in the continuum of HIV care. In addition to scaling up condom and CCL access, evaluating the feasibility of novel biomedical interventions, including antiretroviral pre-exposure prophylaxis and early antiretroviral therapy for MSM living with HIV in Cameroon, is also warranted.

## Introduction

Globally, it has been observed that HIV prevalence among men who have sex with men (MSM) significantly exceeds HIV prevalence in the general population, even in the context of generalized epidemics [1–3]. Across sub-Saharan Africa, HIV prevalence is estimated to be approximately 5% in the general population and 17.9% among MSM [1]. The few published studies from West Africa consistently report higher HIV prevalence among MSM than in the general population, with HIV prevalence estimates of 13.5% among MSM in Nigeria, 16.3% in Burkina Faso and 21.8% in Senegal [1,2,4–6]. Individual-, network-, community-level and policy-level factors noted to contribute to the higher risk of acquisition and transmission of HIV and other sexually transmitted infections (STIs) among MSM have been found to be prevalent in Central and West Africa [5,7,8].

With over 550,000 people living with HIV in Cameroon, the prevalence of HIV among reproductive-age adults in Cameroon is estimated to be 4.3%, which represents a mature and widespread generalized epidemic [9,10]. In Douala and Yaoundé, the two largest cities of the country, HIV prevalence among reproductive-age adults is estimated to be 4.6% and 6.3%, respectively [10].

MSM were recently listed as a priority group in the Cameroon government's “National Strategic Plan for HIV, AIDS, and STIs: 2011–2015,” along with goals including strengthening HIV-prevention programmes and building capacity for HIV health services that serve MSM [11]. The higher biological risks of HIV acquisition and transmission associated with unprotected anal intercourse (UAI) compared to other forms of sexual intercourse make MSM an important target population for HIV-prevention efforts [12]. However, only one HIV prevalence estimate from programmatic data in Douala is available to date for MSM; in this 2007 study, which used convenience sampling, HIV prevalence was estimated to be 18.4% [13].

Established individual-level risks for HIV acquisition and transmission among MSM in the region that are modifiable include UAI, inconsistent use of condom-compatible lubricants (CCLs), a high number of male partners, drug use and syphilis co-infection [1]. In a recent study, UAI in the past 6 months was frequent among MSM in Douala, as was having one or more female sexual partners [7]. Bisexual concurrency and bisexual partnerships among MSM have been observed in studies in Nigeria, Senegal and southern Africa [5,14,15]. Inconsistent condom use with male and female partners was common among MSM in one Togo study, and in a study conducted in Nigeria, it was associated with prevalent HIV infection, as was having been the receptive partner in anal intercourse in the past 6 months [5,16]. Other factors associated with prevalent HIV infection among MSM in Nigeria and Senegal were older age and having a symptomatic STI [5,15].

Network-level factors that may impact HIV-transmission risk include sexual network size, STI prevalence, levels of peer education, knowledge of HIV status within the population and network tendencies for drug use or transactional sex [1]. Community-level factors that may contribute to HIV risk include high community viral load and suboptimal coverage or uptake of healthcare services [1]. Additionally, the social stigma surrounding HIV, sexual identities and homosexuality in Cameroon may deter MSM from seeking voluntary HIV counselling and testing (VCT) or other health services [17–20]. Perceived stigma, including fear of seeking healthcare and refraining from disclosing same-sex practices to a health professional, and enacted discrimination, including denial of healthcare access based on sexuality, were frequently reported by MSM in Senegal and southern Africa, and were associated with increased sexual risk practices and prevalent HIV infection [21–26]. Similar to most countries in sub-Saharan Africa, sexual relationships between men are both criminalized and highly stigmatized in Cameroon, and prosecution can result in up to 5 years of imprisonment [8]; physical violence from law enforcement is also a reality for some MSM, posing challenges to HIV programming [8,27–29].

In light of the unique needs of MSM within generalized epidemics, and the limited data available on this vulnerable population in Cameroon, we aimed to describe the socio-demographic and behavioural characteristics of MSM in Douala and Yaoundé, determine the age-stratified HIV and syphilis prevalence in both cities, and investigate the individual-, network- and community-level factors associated with HIV infection among this population.

## Methods

### Study population

This cross-sectional study was conducted in August–September 2011 at two community-based organizations (CBOs) that provide targeted services to MSM: Alternatives-Cameroun in Douala and the Cameroon National Association for Family Welfare (CAMNAFAW) in Yaoundé. The interviewers were MSM community volunteers from Alternatives-Cameroun, Humanity First and CAMNAFAW. The MSM sensitivity trainings for interviewers were conducted at the Association Camerounaise pour le Marketing Social (ACMS) conference rooms in Douala and Yaoundé. Men aged 18 years or older who reported engaging in penile–anal or oral intercourse with another man in the past 12 months were eligible for the study. Participants were recruited using respondent-driven sampling (RDS) [30], a sampling technique that enables estimation of proportions and regression modelling while controlling for non-random social network structures that bias peer-based recruitment. Seven seeds heterogeneous in sexual identity and sexual role preference were selected through existing community contacts to begin the recruitment process in each city. Upon enrolment in the study, all individuals were given three uniquely coded coupons to refer other MSM to the study. The CBOs worked with the research team to identify the initial seeds, screen study participants for eligibility and interview participants after receiving informed consent.

Sample-size calculations were based on the ability to detect a 15% change in the prevalence of condom use at last anal intercourse over time from 60% at baseline, with a design effect of 2, a significance level of 0.05 and a power of 80%, yielding 241 men for each city.

All participants provided written informed consent. The study was approved by the Cameroon National Ethics Committee, and the secondary analysis of the study data was approved by the Johns Hopkins Bloomberg School of Public Health.

### Data collection

Participants completed an interviewer-administered structured questionnaire containing questions on: socio-demographics; network size; sexual behaviours, including condom and lubricant use (always vs. often, sometimes or never); experiences of STI symptoms; access to CBO-run MSM centres (which included outreach services); access to free condoms; VCT experiences; knowledge of HIV transmission, prevention and treatment (a composite score from 13 questions); and perceived social support for condom use (a composite score from eight questions, including support from partners, family and peers). Interviews were conducted in French or English, and they were recorded in French.

After participants received pre-test counselling, approximately 4 ml blood specimen was collected from them by a Global Viral Cameroon phlebotomist and tested to confirm HIV and syphilis serostatus, followed by post-test counselling on the same day. Men who screened positive for HIV or syphilis were referred to appropriate health services. All participants were reimbursed 1000 CFA franc (US$2) for completing the questionnaire and an additional 1000 CFA franc (US$2) for each peer referred into the study. All participants received free VCT, condoms and CCLs. Participants were also given access to peer education, support groups and linkage to HIV care.

### Laboratory testing

Specimen processing and testing were conducted by staff from Global Viral Cameroon at the field sites. The national HIV surveillance algorithm for second-generation surveillance of HIV, adopted by the Ministry of Public Health of Cameroon, was used to measure current HIV status, including Determine^®^ HIV-1/2 (Inverness Medical, Chiba, Japan) and Human HEXAGON HIV 1 + 2 (Human GmBh, Wiesbaden, Germany). All indeterminate and positive samples and 15% of the negative samples were transferred to the Global Viral Cameroon Yaoundé laboratory for fourth-generation HIV enzyme-linked immunosorbent assay (ELISA), which detects antibodies to HIV-1/2 and the p24 antigen (whose presence indicates a possible seroconversion). Screening for syphilis was performed according to the national algorithm in Cameroon using Rapid Protein Reagin (RPR; SGM Italia, Roma, Italy) and *Treponema pallidum* haemagglutination assay (TPHA; Fortress Diagnostics Limited, Antrim, UK). Global Viral Cameroon was responsible for blood specimen collection, laboratory testing and serology data management.

### Statistical analysis

ACMS and CARE International-Cameroon managed study data. Questionnaire data were double entered into the CSPro (version 4.0) software, exported into SPSS for data cleaning by ACMS and then exported to Stata/SE (version 11.2) for data analysis.

To minimize biases associated with chain referral sampling, weights were created in Stata/SE version 11.2 using the RDSII estimator to account for the effect of differences in the social network sizes of participants. Weights were based on the transition matrix for the dependent variable, current HIV status. Network size was assessed using the response from the latter of two questions: “How many men who have had oral or anal sex with men in the last 12 months do you know, who also know you and live in this city?” and “among these men that you know personally, how many of them are 18 years and older?” Homophily (range: −1 to +1) was assessed to evaluate the preferences of individuals to recruit MSM with the same HIV status [31].

Bivariate logistic regression models were used to estimate the unadjusted association between HIV infection and covariates selected based on our knowledge and the published literature. RDS-weighted prevalence and bootstrapped confidence intervals were calculated for all variables explored in regression modelling. Multivariate logistic regression models were built to estimate the adjusted association between current HIV status and covariates, with age forced into all models regardless of statistical significance. The Akaike information criterion (AIC) was used to favour the most parsimonious models. Bivariate and multivariate logistic regression models were also built with RDS weighting. *p*-values < 0.05 were used to indicate statistical significance. We further compared the associations between binary covariates using the Pearson chi-square test.

## Results

A total of 295 men were screened in Douala, of whom 272 participated. In Yaoundé, a total of 246 individuals were screened, resulting in 239 participants. The median number of descendants per seed was 32 (range 6–99) in Douala and 31 (range 2–88) in Yaoundé. In Douala, the median number of waves per seed was 6 (range 1–8); homophily for HIV status was −0.04 among the HIV-negative group and 0.06 in the group living with HIV. In Yaoundé, the median number of waves per seed was five (range 1–9); homophily for HIV status was 0.004 for the HIV-negative group and 0.06 for the group living with HIV. In both samples, RDS network homophily was close to 0, which may indicate a close approximation to random recruitment. The majority (77.9%; 398/511) reported that they would have given a coupon to their recruiter (an indicator of the reciprocal ties assumption [32]).

Overall, the median age was 24 years (range 18–51, interquartile range (IQR) 21–28). In both cities, the majority had completed secondary education and were single. Sixty-two percent of MSM in the overall sample identified as bisexual, compared with 28.6% who identified as gay or homosexual and 9.8% as MSM or other. Ninety-eight percent of all participants reported having penile-anal intercourse in the past 12 months. Median age of sexual debut with another man was 19 (IQR 17–22) (Table 1).

**Table 1:** Characteristics of MSM recruited from Douala (*n*=272) and Yaoundé (*n*=239) in Cameroon, 2011

		Douala		Yaoundé	
			
	All *n* (%)	*n* (%)	RDS-weighted % (95% CI)	*n* (%)	RDS-weighted % (95% CI)
Total	511 (100)	272 (100)	–	239 (100)	–
Age, median (IQR) (years)	24 (21–28)	23 (21–27)	–	25 (21–28)	–
18–23	238 (46.6)	142 (52.2)	57.6 (50.6–64.6)	96 (40.2)	42.1 (34.0–50.3)
24–29	185 (36.2)	85 (31.3)	29.5 (23.1–35.9)	100 (41.8)	42.5 (34.7–50.3)
30+	88 (17.2)	45 (16.5)	12.9 (8.2–17.6)	43 (18.0)	15.3 (8.9–21.8)
Education					
Primary or less	26 (5.1)	20 (7.4)	7.6 (4.0–11.2)	6 (2.5)	2.7 (0.1–5.2)
Secondary	341 (66.7)	183 (67.3)	70.1 (64.1–76.1)	158 (66.1)	69.8 (63.5–76.2)
Higher than secondary	144 (28.2)	69 (25.8)	22.3 (16.5–28.0)	75 (31.5)	27.5 (21.5–33.6)
Occupational status					
Student or apprentice	204 (39.9)	116 (42.7)	46.5 (40.0–53.0)	88 (36.8)	36.9 (29.3–44.5)
Employed	248 (48.5)	126 (46.3)	45.0 (38.1–51.8)	122 (51.1)	48.8 (41.0–56.6)
Unemployed	59 (11.6)	30 (11.0)	9.1 (5.3–12.9)	29 (12.1)	14.3 (7.7–20.9)
Christian religion	456 (89.2)	231 (86.2)	87.9 (83.2–92.6)	220 (92.4)	91.6 (87.1–96.1)
Network size, median (IQR)	12 (6–25)	13 (5–25)	–	12 (6–24.5)	–
Sexual identity					
Bisexual	317 (62.0)	171 (62.9)	65.9 (59.3–72.5)	146 (61.1)	62.1 (54.1–70.1)
Gay or homosexual	144 (28.6)	70 (26.3)	22.7 (16.8–28.6)	73 (31.3)	28.7 (21.1–36.4)
MSM	41 (8.0)	26 (9.6)	9.4 (5.6–13.2)	15 (6.3)	8.4 (3.5–13.3)
Other	9 (1.8)	4 (1.5)	–	5 (2.1)	–
Relationship status					
Single	425 (84.2)	230 (84.6)	85.6 (80.5–90.6)	194 (83.3)	87.3 (82.2–92.4)
In a relationship or married	77 (15.2)	39 (14.3)	14.4 (9.4–19.5)	38 (15.9)	12.7 (7.6–17.8)
Separated, widowed or other	3 (0.6)	2 (0.8)	–	1 (0.5)	–
Sexual role preference					
Insertive	223 (45.0)	118 (43.9)	45.4 (37.4–53.5)	110 (46.2)	46.8 (38.7–54.9)
Receptive	160 (31.6)	85 (31.6)	29.8 (23.6–36.0)	75 (31.5)	32.2 (24.7–39.8)
Versatile	119 (23.5)	66 (24.5)	24.7 (17.9–31.6)	53 (22.3)	21.0 (15.0–26.9)
Age of sexual debut with another man, median (IQR) (years)	19 (17–22)	19 (17–22)	–	19 (17–21)	–

CI, confidence interval; IQR, interquartile range; MSM, men who have sex with men; RDS, respondent-driven sampling.

Responses to questions on health service uptake, HIV knowledge, social support and sexual practices are presented in Table 2. Men in Yaoundé were much less likely to access CBO services targeting MSM than men in Douala (33.7% vs. 66.1%, χ^2^(1), *p*<0.001). No difference was observed in ever receiving free condoms (74.2% vs. 68.8%, χ^2^(1), *p*=0.2). In both cities, a large proportion of men reported sex with males and females (46.2%) and experienced STI symptoms in the previous year (34.6%). Inconsistent use of condoms with regular male partners (64.1%; 273/426) and casual male and female partners (48.5%; 195/402) was common, as were condom slippage and breakage (43.7%; 216/494). Ninety percent of MSM who used condoms also reported using lubricant. Of these men, 26.3% (124/472) reported using lotion, saliva, Vaseline or other condom-incompatible lubricants.

**Table 2:** Health service uptake, HIV knowledge, social support and sexual practices among MSM recruited from Douala (*n*=272) and Yaoundé (*n*=239) in Cameroon, 2011

		Douala		Yaoundé	
			
	All *n* (%)	*n* (%)	RDS-weighted % (95% CI)	*n* (%)	RDS-weighted % (95% CI)
Ever accessed CBO service targeting MSM	302 (59.1)	199 (74.3)	66.1 (57.6–74.6)	100 (42.0)	33.7 (26.6–40.8)
Ever received free condoms	355 (71.7)	196 (74.2)	71.6 (64.3–78.9)	159 (68.8)	62.2 (54.1–70.4)
HIV knowledge composite, median score % correct (IQR)	85 (77–92)	85 (77–92)	–	85 (77–92)	–
Social support on condom use, median score % (IQR)	63 (38–88)	63 (38–88)	–	75 (50–88)	–
Ever had sexual intercourse after drinking alcohol	338 (66.1)	156 (57.4)	57.5 (49.6–64.8)	182 (76.2)	73.2 (65.9–80.4)
Ever had sexual intercourse after taking a drug	43 (8.4)	18 (6.6)	5.0 (1.7–8.2)	25 (10.5)	9.7 (5.8–13.5)
In the past 12 months					
Had male and female sexual partners	236 (46.2)	125 (46.1)	48.3 (41.2–55.5)	111 (46.4)	49.6 (42.3–56.9)
Experienced STI symptom(s)	175 (34.5)	80 (29.9)	30.5 (23.6–37.4)	95 (39.9)	38.9 (31.5–46.4)
Number of male partners, median (IQR)	3 (2–5)	3 (2–5)	–	3 (2–5)	–
1–3	304 (59.5)	171 (62.9)	68.1 (61.3–74.8)	133 (55.7)	62.9 (55.5–70.3)
4+	207 (40.5)	101 (37.1)	31.9 (25.2–38.7)	106 (44.3)	37.1 (29.8–44.5)
Inconsistent condom use with regular male partner(s)*	273 (64.1)	123 (56.9)	58.4 (49.1–67.8)	100 (42.2)	42.9 (36.3–49.4)
Inconsistent condom use with casual partner(s)**	195 (48.5)	98 (46.9)	36.2 (29.4–43.1)	96 (50.3)	44.4 (33.6–55.2)
Condom torn or removed involuntarily during sex	216 (43.7)	118 (44.9)	46.3 (39.2–53.5)	98 (42.4)	41.6 (34.4–48.7)
Generally use lubricant with condom	460 (90.0)	235 (89.4)	88.5 (83.8–93.2)	219 (92.4)	93.8 (90.6–97.0)
CCLs	348 (73.7)	186 (75.6)	72.2 (65.3–79.0)	162 (71.7)	66.8 (57.9–75.7)
Lotion, saliva, Vaseline or other	124 (26.3)	60 (24.4)	27.8 (20.9–34.8)	64 (28.3)	33.2 (24.3–42.1)
Gave a woman money or objects in exchange for sexual intercourse	25 (4.9)	14 (5.2)	5.7 (1.8–9.7)	11 (4.6)	4.3 (0.8–7.8)
Gave a man money or objects in exchange for sexual intercourse	30 (5.9)	15 (5.5)	4.2 (1.6–6.8)	15 (6.3)	4.4 (1.8–6.9)

* In Douala, *n*=52 (19.4%) did not have a regular partner. In Yaoundé, *n*=32 (13.4%) did not have a regular partner.

** In Douala, *n*=59 (22.0%) did not have a casual partner; in Yaoundé, *n*=48 (20.1%) did not have a casual partner.

CBO, community-based organization; CCLs, condom-compatible lubricant; CI, confidence interval; IQR, interquartile range; MSM, men who have sex with men; RDS, respondent-driven sampling; STI, sexually transmitted infection.

As presented in Table 3, crude and RDS-weighted HIV prevalence were 28.6% (73/255) and 25.5% (95% CI 19.1–31.9) in Douala and 47.3% (98/207) and 44.4% (95% CI 35.7–53.2) in Yaoundé. Age-stratified prevalence is presented in Figure 1. In Douala, only 17 (6.3%) MSM refused to be tested; in Yaoundé, this number was higher (13.4%, *n*=32). An association between having a history of VCT and refusing testing in the study was observed in Yaoundé, although it did not reach statistical significance (15.2% vs. 4.9%, *p*=0.08). Refusal was not correlated with age, education level, age of sexual debut, condom use, receptive sexual role preference, number of male sexual partners in the past 12 months or perceived social support for condom use (all *p*>0.10). Active syphilis infection was detected in only one participant per city.

**Figure 1:** Unadjusted HIV prevalence stratified by age category among MSM from Douala (*n*=255) and Yaoundé (*n*=207) in Cameroon, 2011.

**Table 3:** HIV and syphilis prevalence among MSM in Douala (*n*=272) and Yaoundé (*n*=239) in Cameroon, 2011

		Douala		Yaoundé	
			
	All *n* (%)	*n* (%)	RDS-weighted % (95% CI)	*n* (%)	RDS-weighted % (95% CI)
HIV prevalence*					
All ages	171 (37.0)	73 (28.6)	25.5 (19.1–31.9)	98 (47.3)	44.4 (35.7–53.2)
Age 18–23	54 (24.9)	27 (20.6)	14.6 (6.7–22.6)	27 (31.4)	20.8 (8.5–33.1)
Age 24–29	79 (47.0)	27 (33.3)	30.0 (16.9–43.1)	52 (60.0)	59.7 (48.8–70.6)
Age 30+	38 (49.4)	19 (44.2)	43.8 (27.9–59.7)	19 (55.9)	55.9 (29.7–82.1)
Refused HIV testing	49 (9.6)	17 (6.3)	4.2 (1.8–6.6)	32 (13.4)	12.7 (7.3–18.2)
Active syphilis infection**	2 (0.4)	1 (0.5)	–	1 (0.4)	–

* HIV status determined by two rapid tests, and confirmation by enzyme-linked immunosorbent assay (ELISA) test.

** Positive in both Rapid Protein Reagin (RPR) and *Treponema pallidum* hemagglutination assay (TPHA) tests.

CI, confidence interval; MSM, men who have sex with men; RDS, respondent-driven sampling.

### Factors associated with HIV infection

#### Douala

Bivariate and multivariate analyses of the individual-, network- and community-level factors associated with HIV infection in Douala are presented in Table 4. The adjusted RDS-weighted odds of having HIV increased for every year rise in age for MSM aged 18–29 (aOR 1.13 per year, 95% CI 1.01–1.27), then plateaued for MSM aged 30 and older (aOR 0.89 per year, 95% CI 0.72–1.09). Preference for the receptive sexual role (aOR 2.33, 95% CI 1.02–5.32) was associated with increased odds of having HIV in both RDS-naïve and RDS-weighted multivariate analyses. Individuals living with HIV were more likely to have ever accessed a CBO service for MSM compared to individuals without HIV (aOR 4.88, 95% CI 1.63–14.63). Individuals who had sex with women (OR 0.50, 95% CI 0.26–0.96) or used condoms inconsistently with casual partners (OR 0.40, 95% CI 0.18–0.89) in the past 12 months were less likely to be living with HIV in bivariate analysis.

**Table 4:** Bivariate and multivariate models of the individual-, network- and community-level factors associated with HIV infection among MSM in Douala (*n*=255) in Cameroon, 2011

	Living with HIV (*n*=73)	HIV-negative (*n*=182)	OR (95% CI)	RDS-weighted OR (95% CI)	aOR (95% CI)	*p*	RDS-weighted aOR (95% CI)	*p*
Age								
Per-year increase for MSM aged 18–29	54 (74.0)	158 (86.8)	**1.13 (1.04–1.23)**	**1.14 (1.04–1.25)**	**1.15 (1.04–1.27)**	**0.005**	**1.13 (1.01–1.27)**	**0.03**
Per-year increase for MSM aged 30+	19 (26.0)	24 (13.2)	0.86 (0.73–1.01)	0.86 (0.72–1.03)	0.86 (0.71–1.04)	0.1	0.89 (0.72–1.09)	0.3
Education: higher than secondary	20 (27.4)	44 (24.2)	1.18 (0.64–2.19)	1.05 (0.52–2.14)	–	–	–	–
Occupational status								
Student or apprentice	17 (23.3)	92 (50.6)	Ref	Ref	–	–	–	–
Employed	47 (64.4)	74 (40.7)	**3.44 (1.82–6.48)**	**3.66 (1.90–7.04)**	–	–	–	–
Unemployed	9 (12.3)	16 (8.8)	**3.04 (1.16–8.00)**	2.09 (0.72–6.10)	–	–	–	–
Christian religion	64 (87.7)	160 (87.9)	0.98 (0.43–2.24)	1.05 (0.40–2.72)	–	–	–	–
Sexual identity: gay	29 (39.7)	39 (21.4)	**2.42 (1.34–4.35)**	**2.53 (1.27–5.03)**				
Relationship status: single	54 (75.0)	160 (87.9)	**0.41 (0.21–0.83)**	0.48 (0.21–1.08)	–	–	–	–
Sexual role preference: receptive	33 (45.2)	48 (26.4)	**2.30 (1.31–4.06)**	**2.23 (1.15–4.31)**	**2.96 (1.50–5.82)**	**0.002**	**2.33 (1.02–5.32)**	**0.045**
Age of sexual debut								
5–17	28 (38.4)	62 (34.1)	Ref	Ref	–	–	–	–
18+	45 (61.6)	120 (65.9)	0.83 (0.47–1.46)	0.66 (0.35–1.26)	–	–	–	–
Ever accessed CBO service targeting MSM	65 (89.0)	125 (68.7)	**3.71 (1.67–8.23)**	**4.33 (1.75–10.75)**	**3.22 (1.17–8.89)**	**0.048**	**4.88 (1.63–14.63)**	**0.005**
Ever received free condoms	60 (84.5)	124 (70.1)	**2.33 (1.14–4.78)**	**2.82 (1.22–6.53)**	–	–	–	–
Generally use CCLs with condoms	59 (81.9)	113 (62.8)	**2.69 (1.37–5.27)**	**2.89 (1.17–7.16)**	**2.32 (1.01–5.34)**	**0.049**	2.29 (0.95–5.53)	0.07
HIV knowledge composite score, per 20% increase	85 (8)	85 (23)	0.85 (0.56–1.29)	0.81 (0.49–1.33)	–	–	–	–
Social support composite score, per 20% increase	75 (50)	63 (50)	1.06 (0.88–1.29)	1.11 (0.88–1.40)	–	–	–	–
In the past 12 months								
Had male and female sexual partners	26 (35.6)	89 (48.9)	0.58 (0.33–1.01)	**0.50 (0.26–0.96)**	–	–	–	–
Any STI symptom	26 (36.1)	49 (27.4)	1.50 (0.84–2.68)	1.38 (0.70–2.74)	–	–	–	–
Number of male partners								
1–3	46 (63.0)	116 (63.7)	Ref	Ref	–	–	–	–
4+	27 (37.0)	66 (36.3)	1.03 (0.59–1.81)	1.40 (0.72–2.70)	–	–	–	–
Inconsistent condom use: regular male partner(s)	36 (60.0)	79 (54.9)	1.23 (0.67–2.28)	1.30 (0.64–2.66)	–	–	–	–
Inconsistent condom use: casual partner(s)	15 (28.9)	76 (52.4)	**0.37 (0.18–0.73)**	**0.40 (0.18–0.89)**	–	–	–	–
Condom slippage or breakage	77 (44.0)	34 (47.2)	1.14 (0.66–1.97)	1.28 (0.68–2.44)	–	–	–	–

Bold indicates *p*-value<0.05. aOR, adjusted odds ratio; CBO, community-based organization; CCL, condom-compatible lubricant; CI, confidence interval; IQR, interquartile range; MSM, men who have sex with men; OR, odds ratio; RDS, respondent-driven sampling; STI, sexually transmitted infection.

#### Yaoundé

In multivariate analysis of the Yaoundé sample (Table 5), factors independently associated with having HIV infection were age (aOR 1.14, 95% CI 1.02–1.26 if aged 18–29; aOR 0.84, 95% CI 0.65–1.07 if aged ≥30) and general use of CCLs with condoms (aOR 2.44, 95% CI 1.19–4.97). Men living with HIV were more likely to have four or more partners in the past 12 months, although this did not reach statistical significance (aOR: 1.88, 95% CI: 0.95–3.71).

**Table 5:** Bivariate and multivariate models of the individual-, network- and community-level factors associated with HIV infection among MSM in Yaoundé (*n*=207) in Cameroon, 2011

	Living with HIV (*n*=98)	HIV-negative (*n*=109)	OR (95% CI)	RDS-weighted OR (95% CI)	aOR (95% CI)	*p*	RDS-weighted aOR (95% CI)	*p*
Age								
Per-year increase for MSM aged 18–29	79 (80.6)	94 (86.2)	**1.15 (1.05–1.25)**	**1.17 (1.06–1.30)**	**1.11 (1.02–1.22)**	**0.02**	1.14 (1.02–1.26)	**0.02**
Per-year increase for MSM aged 30+	19 (19.4)	15 (13.8)	**0.80 (0.65–0.98)**	**0.79 (0.62–1.00)**	0.84 (0.67–1.04)	0.1	0.84 (0.65–1.07)	0.2
Education: higher than secondary	30 (30.6)	35 (32.1)	0.93 (0.52–1.68)	0.93 (0.48–1.83)	–	–	–	–
Occupational status								
Student or apprentice	28 (28.6)	46 (42.2)	Ref	Ref	–	–	–	–
Employed	53 (54.1)	51 (46.8)	1.71 (0.93–3.13)	1.68 (0.83–3.38)	–	–	–	–
Unemployed	17 (17.4)	12 (11.0)	2.33 (0.97–5.59)	1.88 (0.69–5.11)	–	–	–	–
Christian religion	92 (93.9)	98 (89.9)	1.72 (0.61–4.84)	1.23 (0.38–3.97)	–	–	–	–
Sexual identity: gay	38 (38.8)	27 (24.8)	**1.92 (1.06–3.49)**	**2.36 (1.19–4.68)**	–	–	–	–
Relationship status: single	83 (85.6)	92 (87.6)	0.84 (0.37–1.89)	1.36 (0.54–3.46)	–	–	–	–
Sexual role preference: receptive	35 (35.7)	32 (29.4)	1.34 (0.75–2.40)	1.35 (0.69–2.62)	–	–	–	–
Age of sexual debut								
5–17	34 (34.7)	31 (28.4)	Ref	Ref	–	–	–	–
18+	64 (65.3)	78 (71.6)	0.75 (0.42–1.35)	0.67 (0.34–1.31)	–	–	–	–
Ever accessed CBO service targeting MSM	43 (43.9)	44 (40.4)	1.15 (0.66–2.01)	0.95 (0.51–1.78)	–	–	–	–
Ever received free condoms	70 (72.2)	68 (66.7)	1.30 (0.71–2.38)	1.25 (0.62–2.49)	–	–	–	–
Generally use CCLs with condoms	74 (76.3)	59 (54.6)	**2.67 (1.46–4.88)**	**2.42 (1.19–4.91)**	**1.97 (1.04–3.72)**	**0.04**	**2.44 (1.19–4.97)**	**0.02**
HIV knowledge composite score, per 20% increase	85 (15)	85 (15)	0.90 (0.58–1.40)	1.00 (0.60–1.69)	–	–	–	–
Social support composite score, per 20% increase	75 (38)	63 (38)	1.14 (0.91–1.43)	1.18 (0.91–1.53)	–	–	–	–
In the past 12 months					–	–	–	–
Had male and female sexual partners	41 (41.8)	52 (47.1)	0.79 (0.45–1.37)	0.63 (0.34–1.18)				
Any STI symptom	48 (49.0)	43 (39.8)	1.45 (0.84–2.52)	1.81 (0.96–3.42)	–	–	–	–
Number of male partners								
1–3	46 (46.9)	71 (65.1)	Ref	Ref	Ref	–	Ref	–
4+	52 (53.1)	38 (34.9)	**2.11 (1.21–3.69)**	**2.25 (1.19–4.28)**	1.81 (0.99–3.28)	0.05	1.88 (0.95–3.71)	0.07
Inconsistent condom use: regular male partner(s)	59 (67.8)	71 (75.5)	0.68 (0.36–1.31)	0.76 (0.36–1.59)	–	–	–	–
Inconsistent condom use: casual partner(s)	43 (56.6)	43 (49.4)	1.33 (0.72–2.47)	1.55 (0.76–3.15)	–	–	–	–
Condom slippage or breakage	46 (45.1)	41 (42.3)	0.89 (0.51–1.56)	0.69 (0.36–1.32)	–	–	–	–

Bold indicates *p*-value<0.05. aOR, adjusted odds ratio; CCL, condom-compatible lubricant; CI, confidence interval; MSM, men who have sex with men; OR, odds ratio; RDS, respondent-driven sampling; STI, sexually transmitted infection.

## Discussion

The high HIV prevalence and inconsistent use of condoms and CCLs observed in this study highlight that MSM are a priority population for HIV prevention, treatment and care services in Douala and Yaoundé. Furthermore, these data suggest that HIV risks are not evenly distributed given the significant differences in HIV prevalence between cities and between MSM sub-populations [1].

The individual-level factors found to be associated with HIV infection indicate that future HIV programming and interventions in Cameroon should address both behavioural and structural hurdles relevant to MSM. Consistent with data from other countries of sub-Saharan Africa [15,16,24], condom breakage and slippage and inconsistent condom use were common among this sample. CCLs, which decrease the risk of condom breakage, were also used inconsistently [33], suggesting that increased access to quality condoms and CCLs is essential [34,35]. While maximizing the use of condoms and CCLs is necessary in decreasing HIV risks among MSM, likely it will not be sufficient to change the trajectory of the epidemic given the high transmission probability of HIV infection associated with UAI, as observed in other settings [1,34]. The prevalence of active syphilis was low, as observed in other countries in the region [4,5]; however, a high proportion of participants reported experiencing STI symptoms, highlighting another network-level risk factor potentiating HIV transmission within the sexual network. Increasing the capacity for routine STI diagnosis, particularly for genitourinary infections, and linkage to treatment tailored towards MSM should be incorporated to support HIV-prevention programmes [15,36].

MSM in Douala who reported a preference of being the receptive partner during anal intercourse were more likely to identify as gay and be living with HIV. This not only affirms existing data demonstrating the increased HIV acquisition risk associated with unprotected receptive anal intercourse [37] but also echoes previous studies conducted in African settings in which self-reporting as gay was associated with higher odds of living with HIV compared to other MSM in the African setting [24,38]. Given that antiretroviral pre-exposure prophylaxis (PrEP) and rectal microbicides have been identified as research priorities for African MSM [39], and that rectal microbicides are currently in Phase II trials that are enrolling MSM from the African continent [34], evaluating the feasibility of novel biomedical interventions for sub-populations of MSM in Cameroon with significant HIV acquisition risks may be appropriate [34,40,41]. However, the cost-effectiveness of implementationing such biomedical interventions requires further research [42]. In addition, exploring increased antiretroviral therapy (ART) for MSM living with HIV likely represents an important strategy for preventing the transmission of HIV to sexual partners. However, the limited availability of ART for people living with HIV who are currently eligible for treatment, which has been documented in Cameroon, also needs to be addressed in order for ART-based strategies for people at risk for the acquisition or transmission of HIV to be effective [43].

A significant proportion of the MSM in our sample were living with HIV by the age of 18–23, indicating a high risk for HIV acquisition for men under 18 in these settings [24,44]; however, men under 18 have traditionally been excluded from HIV surveillance and prevention programmes [1]. Confidential youth sexuality counselling hotlines, web-based education and social marketing campaigns may be useful in reaching younger MSM with HIV programmes [36,37].

While our study did not include a detailed assessment of social stigma, other studies have demonstrated that stigma limits the provision and uptake of HIV prevention, treatment and care for MSM in the region [18,19,27]. Uptake of services delivered by targeted CBO providers such as Alternatives-Cameroun in Douala was high in our study, suggesting that community-based approaches can spread information-leveraging networks of MSM despite the contextual barriers. There was limited uptake of services in Yaoundé and higher refusal of HIV testing in the study; to the best of our knowledge, MSM-tailored HIV programmes were new and in development at the time of this surveillance project. The historically limited services may partially explain the higher HIV prevalence observed among MSM in Yaoundé as compared to Douala, although these participants also tended to be older and report more male partners, drug and alcohol use, and STI symptoms.

Data on the proportion of MSM living with HIV who were eligible for treatment, or who were actually on treatment, were not collected in this study. However, consistent data highlight the importance of addressing the needs of people living with HIV, including linkage to care, to optimize their own health and prevent onward transmission to other men and to women [45]. In Cameroon, only half of all patients eligible for treatment are estimated to be receiving ART, and ART stock outages at health facilities are frequent [43]. Given the significant stigma and discrimination that have been documented as affecting MSM in Cameroon, MSM living with HIV may be at higher risk of being unaware of their diagnosis or not achieving viral suppression [8,20,46]. MSM community groups have long been known to play essential roles in the HIV response, and the data collected here suggest that community-driven approaches should be scaled up to increase uptake of VCT and support linkage to HIV care, treatment and adherence support for those eligible [47,48].

The cross-sectional design of this study does not allow us to assume causality of the associations present in the data. There are several limitations to the generalizability of the HIV prevalence estimates reported in this study, which included individuals who reported receptive or insertive anal intercourse in the past 12 months. The generalizability of the results for MSM living in smaller urban centres and rural settings is unknown given that recruitment occurred in two large cities. Similarly, as our sample was predominantly young and educated, the results may not pertain to older MSM or individuals with lower educational status. Future studies to address these gaps could be conducted. The modest sample size may have reduced our statistical ability to detect other associations [49]. Due to the high refusal of HIV testing during the study in Yaoundé (13.4%), we were unable to assess the potential for bias in the HIV prevalence estimate from this city. However, RDS network homophily was close to 0, which may indicate minimal recruitment bias based on HIV status. Data on self-reported HIV status and the percentage of undiagnosed men were not available, which limit our interpretation of the association between knowledge of one's own HIV status and behavioural factors such as inconsistent use of condoms and CCLs. This requires further investigation in future studies. Although non-significant, the positive association between having been tested and refusing testing may suggest that individuals who are already aware of their HIV status may be underrepresented in our study.

## Conclusions

These data provide results that can be integrated into HIV programmes for MSM in Cameroon and highlight the importance of targeted HIV prevention, treatment and care services that address all levels of HIV risk. Coordinating behavioural, biomedical and structural interventions, and supporting the work of local CBOs, will be keys to ensuring that HIV-negative MSM receive regular VCT and appropriate prevention services, and that MSM living with HIV are effectively engaged in the continuum of HIV care. Success in the continuum of HIV care necessitates addressing the barriers to the uptake of care, such as concerns about confidentiality and healthcare-related enacted and perceived stigmas [8,20,36]. Protecting the dignity and rights of MSM in healthcare settings and beyond allows for a safe environment for individuals to receive optimal care to protect themselves and their partners [27,29]. Monitoring the success of the next generation of HIV-prevention approaches will require innovative implementation science exploring changes not only in individual-level risks, community viral load and HIV incidence, but also in social and policy-level factors including stigma, discrimination, violence and criminalization.

## References

[1] Beyrer C, Baral SD, van Griensven F, Goodreau SM, Chariyalertsak S, Wirtz AL (2012). Global epidemiology of HIV infection in men who have sex with men. Lancet.

[2] Baral S, Sifakis F, Cleghorn F, Beyrer C (2007). Elevated risk for HIV infection among men who have sex with men in low- and middle-income countries 2000–2006: a systematic review. PLoS Med.

[3] van Griensven F, de Lind van Wijngaarden JW, Baral S, Grulich A (2009). The global epidemic of HIV infection among men who have sex with men. Curr Opin HIV AIDS.

[4] Wade AS, Larmarange J, Diop AK, Diop O, Gueye K, Marra A (2010). Reduction in risk-taking behaviors among MSM in Senegal between 2004 and 2007 and prevalence of HIV and other STIs. ELIHoS Project, ANRS 12139. AIDS Care.

[5] Merrigan M, Azeez A, Afolabi B, Chabikuli ON, Onyekwena O, Eluwa G (2011). HIV prevalence and risk behaviours among men having sex with men in Nigeria. Sex Transm Infect.

[6] National AIDS Control Commitee (2012). UNGASS country progress report: HIV/AIDS Report for Burkina Faso, 2012.

[7] Henry E, Marcellin F, Yomb Y, Fugon L, Nemande S, Gueboguo C (2010). Factors associated with unprotected anal intercourse among men who have sex with men in Douala, Cameroon. Sex Transm Infect.

[8] Human Rights Watch (2010). Criminalizing identities: rights abuses in Cameroon based on sexual orientation and gender identity. http://www.hrw.org/reports/2010/11/04/criminalizing-identities-0.

[9] UNAIDS (2011). HIV and AIDS Estimates. http://www.unaids.org/en/regionscountries/countries/cameroon/.

[10] Cameroon National Institute of Statistics (2012). 2011 Cameroon Demographic and Health Survey and Multiple Indicators Cluster Survey (DHS-MICS): HIV prevalence.

[11] National AIDS Control Committee National strategic plan against HIV, AIDS, and STIs: 2011–2015.

[12] Grulich AE, Zablotska I (2010). Commentary: probability of HIV transmission through anal intercourse. Int J Epidemiol.

[13] (2007). Alternatives-Cameroun.

[14] Beyrer C, Trapence G, Motimedi F, Umar E, Iipinge S, Dausab F (2010). Bisexual concurrency, bisexual partnerships, and HIV among Southern African men who have sex with men. Sex Transm Infect.

[15] Wade AS, Kane CT, Diallo PA, Diop AK, Gueye K, Mboup S (2005). HIV infection and sexually transmitted infections among men who have sex with men in Senegal. AIDS.

[16] Koumagnanou K, Kassegne S, Dodzro KC (2011). HIV prevention among marginal groups: the case of homosexuals in Togo. Glob Health Promot.

[17] Mahajan AP, Sayles JN, Patel VA, Remien RH, Sawires SR, Ortiz DJ (2008). Stigma in the HIV/AIDS epidemic: a review of the literature and recommendations for the way forward. AIDS.

[18] Kalichman SC, Simbayi LC (2003). HIV testing attitudes, AIDS stigma, and voluntary HIV counselling and testing in a black township in Cape Town, South Africa. Sex Transm Infect.

[19] Rankin WW, Brennan S, Schell E, Laviwa J, Rankin SH (2005). The stigma of being HIV-positive in Africa. PLoS Med.

[20] Kalamar M, Maharaj P, Gresh A (2011). HIV-prevention interventions targeting men having sex with men in Africa: field experiences from Cameroon. Cult Health Sex.

[21] Fay H, Baral SD, Trapence G, Motimedi F, Umar E, Iipinge S (2011). Stigma, health care access, and HIV knowledge among men who have sex with men in Malawi, Namibia, and Botswana. AIDS Behav.

[22] Preston DB, D'Augelli AR, Kassab CD, Cain RE, Schulze FW, Starks MT (2004). The influence of stigma on the sexual risk behavior of rural men who have sex with men. AIDS Educ Prev.

[23] Poteat T, Diouf D, Drame FM, Ndaw M, Traore C, Dhaliwal M (2011). HIV risk among MSM in Senegal: a qualitative rapid assessment of the impact of enforcing laws that criminalize same sex practices. PLoS One.

[24] Baral S, Trapence G, Motimedi F, Umar E, Iipinge S, Dausab F (2009). HIV prevalence, risks for HIV infection, and human rights among men who have sex with men (MSM) in Malawi, Namibia, and Botswana. PLoS One.

[25] Baral S, Burrell E, Scheibe A, Brown B, Beyrer C, Bekker LG (2011). HIV risk and associations of HIV infection among men who have sex with men in peri-urban Cape Town, South Africa. BMC Public Health.

[26] Hladik W, Barker J, Ssenkusu JM, Opio A, Tappero JW, Hakim A (2012). HIV infection among men who have sex with men in Kampala, Uganda – a respondent driven sampling survey. PLoS One.

[27] Altman D, Aggleton P, Williams M, Kong T, Reddy V, Harrad D (2012). Men who have sex with men: stigma and discrimination. Lancet.

[28] International Lesbian GB, Trans and Intersex Association (2012). State sponsored homophobia report 2011: a world survey of laws criminalising same-sex sexual acts between consenting adults. http://old.ilga.org/Statehomophobia/ILGA_State_Sponsored_Homophobia_2012.pdf.

[29] Betron M, Gonzalez-Figueroa E (2009). Gender identity, violence, and HIV among MSM and TG: a literature review and a call for screening.

[30] Heckathorn DD (1997). Respondent-driven sampling: a new approach to the study of hidden populations. Soc Probl.

[31] Schonlau M, Liebau E (2012). Respondent driven sampling. Stata J.

[32] Heckathorn DD (2007). Extensions of respondent-driven sampling: analyzing continuous variables and controlling for differential recruitment. Socio Meth.

[33] Stone E, Heagerty P, Vittinghoff E, Douglas JM, Koblin BA, Mayer KH (1999). Correlates of condom failure in a sexually active cohort of men who have sex with men. J Acquir Immune Defic Syndr Hum Retrovirol.

[34] Sullivan PS, Carballo-Dieguez A, Coates T, Goodreau SM, McGowan I, Sanders EJ (2012). Successes and challenges of HIV prevention in men who have sex with men. Lancet.

[35] Charania MR, Crepaz N, Guenther-Gray C, Henny K, Liau A, Willis LA (2011). Efficacy of structural-level condom distribution interventions: a meta-analysis of U.S. and international studies, 1998–2007. AIDS Behav.

[36] World Health Organization (2011). Prevention and treatment of HIV and other sexually transmitted infections among men who have sex with men and transgender people: recommendations for the public health approach 2011.

[37] Beyrer C, Wirtz AL, Walker D, Johns B, Sifakis F, Baral SD (2011). Scenario 3 country studies: Kenya, Malawi, and Senegal. The global HIV epidemics among men who have sex with men.

[38] Lane T, Raymond HF, Dladla S, Rasethe J, Struthers H, McFarland W (2011). High HIV prevalence among men who have sex with men in Soweto, South Africa: results from the Soweto Men's Study. AIDS Behav.

[39] Baral S, Scheibe A, Sullivan P, Trapence G, Lambert A, Bekker LG (2013). Assessing priorities for combination HIV prevention research for men who have sex with men (MSM) in Africa. AIDS Behav.

[40] Grant RM, Lama JR, Anderson PL, McMahan V, Liu AY, Vargas L (2010). Preexposure chemoprophylaxis for HIV prevention in men who have sex with men. N Engl J Med.

[41] World Health Organization (2012). Guidance on pre-exposure oral prophylaxis (PrEP) for serodiscordant couples, men and transgender women who have sex with men at high risk of HIV: recommendations for use in the context of demonstration projects.

[42] Gomez GB, Borquez A, Case KK, Wheelock A, Vassall A, Hankins C (2013). The cost and impact of scaling up pre-exposure prophylaxis for HIV prevention: a systematic review of cost-effectiveness modelling studies. PLoS Med.

[43] National AIDS Control Commitee (2012). UNGASS country progress report: National Monitoring Report on Political Declarations regarding HIV/AIDS in Cameroon.

[44] Sanders EJ, Graham SM, Okuku HS, van der Elst EM, Muhaari A, Davies A (2007). HIV-1 infection in high risk men who have sex with men in Mombasa, Kenya. AIDS.

[45] Cohen MS, Chen YQ, McCauley M, Gamble T, Hosseinipour MC, Kumarasamy N (2011). Prevention of HIV-1 infection with early antiretroviral therapy. N Engl J Med.

[46] Henry E, Awondo P, Fugon L, Yomb Y, Spire B (2012). Coming out of the Nkuta: disclosure of sexual orientation associated with reduced risk behavior among MSM in Cameroon. Arch Sex Behav.

[47] Trapence G, Collins C, Avrett S, Carr R, Sanchez H, Ayala G (2012). From personal survival to public health: community leadership by men who have sex with men in the response to HIV. Lancet.

[48] Beyrer C, Sullivan PS, Sanchez J, Dowdy D, Altman D, Trapence G (2012). A call to action for comprehensive HIV services for men who have sex with men. Lancet.

[49] Johnston LG, Chen YH, Silva-Santisteban A, Raymond HF (2013). An empirical examination of respondent driven sampling design effects among HIV risk groups from studies conducted around the world. AIDS Behav.

# Sub article: A pilot cohort study to assess the feasibility of HIV prevention science research among men who have sex with men in Dakar, Senegal

**DOI:** 10.7448/IAS.16.4.18753

**Published:** 2013-12-02

**Authors:** Fatou Maria Dramé, Emily E Crawford, Daouda Diouf, Chris Beyrer, Stefan D Baral

**Affiliations:** 1 Université Gaston Berger/Enda Santé, Dakar, Senegal; 2 Department of Epidemiology, Center for Public Health and Human Rights, John Hopkins Bloomberg School of Public Health, Baltimore, MD, United States; 3 Enda Santé, Dakar, Senegal

**Keywords:** HIV, socio-economic status, men who have sex with men, Africa, prevention

## Abstract

**Introduction:**

Men who have sex with men (MSM) are disproportionately burdened by HIV in Senegal, across sub-Saharan Africa and throughout the world. This is driven in part by stigma, and limits health achievements and social capital among these populations. To date, there is a limited understanding of the feasibility of prospective HIV prevention studies among MSM in Senegal, including HIV incidence and cohort retention rates.

**Methods:**

One hundred and nineteen men who reported having anal sex with another man in the past 12 months were randomly selected from a sampling frame of 450 unique members of community groups serving MSM in Dakar. These men were enrolled in a 15-month pilot cohort study implemented by a community-based partner. The study included a structured survey instrument and biological testing for HIV, syphilis and hepatitis B virus at two time points.

**Results:**

Baseline HIV prevalence was 36.0% (43/114), with cumulative HIV prevalence at study end being 47.2% (51/108). The annualized incidence rate was 16% (8/40 at risk for seroconversion over 15 months of follow-up, 95% confidence interval 4.6–27.4%). Thirty-seven men were lost to follow up, including at least four deaths. Men who were able to confide in someone about health, emotional distress and sex were less likely to be HIV positive (OR 0.36, *p <* 0.05, 95% CI 0.13, 0.97).

**Conclusions:**

High HIV prevalence and incidence, as well as mortality in this young population of Senegalese MSM indicate a public health emergency. Moreover, given the high burden of HIV and rate of incident HIV infections, this population appears to be appropriate for the evaluation of novel HIV prevention, treatment and care approaches. Using a study implemented by community-based organizations, there appears to be feasibility in implementing interventions addressing the multiple levels of HIV risk among MSM in this setting. However, low retention across arms of this pilot intervention, and in the cohort, will need to be addressed for larger-scale efficacy trials to be feasible.

## Introduction

The HIV epidemic in Senegal has followed a pattern distinct from the epidemics observed in Southern and Eastern African countries such as Kenya and Malawi, with a far more concentrated epidemic among key populations such as men who have sex with men (MSM) and female sex workers [1]. The Senegalese government launched an early and comprehensive effort to prevent HIV infection in the general population [2]. This campaign was deemed a success by many and is, in part, likely responsible for the limited HIV epidemic in the country, which reports an HIV prevalence of 0.8% among reproductive age women and 0.5% among men ages 15–49 [3,4]. More recently, there has been increased study of social factors such as unregulated sex work, stigma and discrimination targeting those at high risk of HIV acquisition and transmission, as well as HIV transmission related to same-sex practices among men [5–11].

MSM have multiple, intersecting drivers of risk and have had a consistently higher risk of HIV acquisition and transmission since the first cases of HIV were discovered [1,12]. This disproportionate burden of HIV in MSM has also been observed in Senegal. Studies dating back nearly ten years have highlighted this disproportionate burden with HIV prevalence among MSM reported to be 22.4% in 2004 and 21.8% in 2007 [11]. Based on these and other data, Dramé reported that HIV prevalence among MSM is approximately 50 times higher than the prevalence observed among reproductive age adults in Senegal according to the most recent demographic and health survey [13]. Finally, the attributable fraction of HIV infections among MSM in Senegal is high; Van Griensven *et al*. estimated that nearly a fifth of prevalent HIV infections among men in Senegal are among MSM. Collectively, these data reinforce the need to address the HIV prevention, treatment and care needs of MSM in Senegal [14].

The definition for social capital is “institutions, relationships, attitudes and values that govern interactions among people and contribute to economic and social development” [15]. The importance of social capital has been increasingly recognized as a major social determinant of health because of its association with health outcomes including chronic disease-related morbidity and mortality and, more recently, sexually transmitted infections [16–26]. Specifically, limited social capital has been associated with higher rates of exchange, survival and commercial sex, and associated with a higher burden of HIV among MSM in Africa [27–30]. HIV infection has also been associated with low social capital; this may be particularly relevant for stigmatized groups such as MSM [31–33]. Development of social capital among MSM has been shown to be limited by enacted stigma [34–36]. And discrimination and stigma affecting MSM are well-documented, not only in Senegal, but throughout sub-Saharan Africa and more broadly around the world [6,7]. Niang *et al*. describe the effect that stigma and discrimination can have on health care-seeking behaviours among MSM in Senegal [6]. When men perceive or experience stigma and discrimination in a health care setting, they are less likely to access health services for STI, resulting in higher rates of untreated STI within sexual networks, thereby mediating HIV transmission [37,38].

MSM face additional challenges in countries where sex between men is criminalized [3]. In Senegal, in 2008, several health promoters working in HIV prevention were arrested under suspicion of being homosexual. These arrests, and the fear of further arrests, had wide-ranging effects on HIV in the community of MSM in Senegal [39]. In response, many non-governmental organizations who had been working in the area of HIV prevention among MSM went into hiding for their own safety. Those who continued distributing prevention materials such as condoms and water-based lubricants saw a marked decrease in the number of men accessing their services. The decreased numbers of men accessing services aimed at the community of MSM also resulted in a lesser availability of social support among MSM. Furthermore, and importantly, health care providers began to fear providing services to MSM following the arrests. This had grave implications for many HIV-positive MSM, who were no longer able to access treatment, either because their provider would no longer see them, or because they feared arrest if they left their home. Some have said that these arrests set HIV prevention efforts back ten years [39].

Stigma and discrimination affect HIV risk and social capital not only by affecting how MSM access prevention and treatment, but also by curbing the presence of research and prevention projects targeting this group in Senegal. A small number of research projects in West Africa has resulted in a limited understanding of what interventions work in communities of MSM in this region [37]. Interventions are difficult to implement, particularly given the constrained legal environment [39]. Community-based organizations of MSM are those with the closest ties to the community and the greatest ability to facilitate interventions [40]. However, these organizations are often not legally registered [13]. Despite these significant challenges, HIV prevention interventions have been effectively implemented for MSM in Senegal [11].

For a population where so much information is left unknown, a cohort study can provide relevant data including prospectively measured HIV incidence [41]. This research project had two primary foci. The first was to assess the feasibility of implementing and retaining participants in a community-driven HIV prevention study in Senegal. The second focus was to describe the study participants in terms of HIV and STI prevalence and incidence, risk behaviours and indicators of social capital at baseline.

## Methods

A prospective cohort study of MSM was conducted from June 2011 to October 2012 in Dakar, Senegal, by members of a community-based organization. Researchers worked with MSM Community Organizations to develop a sampling frame composed of 450 unique individual members of all known MSM organizations in Dakar. Men eligible for the study were at least 18 years of age, members of one of the known MSM organizations in Dakar, had lived in Dakar for at least six months and reported having anal sex with another man in the past 12 months.

Ultimately, 119 men were enrolled in the feasibility cohort study. At baseline, all participants completed an informed consent process, a structured survey instrument and a medical examination conducted by an infectious disease physician. The medical examination included a physical exam and syndromic diagnosis and treatment of STI, or a referral for treatment and follow-up if necessary or preferred by the participants. Participants also provided 10 ml of plasma for testing for HIV, hepatitis B and syphilis, according to the Senegalese national testing algorithm [42]. A subset of the participants also received an exploratory intervention. Because of the small sample size and high loss to follow up, the outcomes of this intervention are not statistically relevant and will not be discussed in this paper.

### Follow up

Thirty-seven participants were lost to follow up between T1 (baseline) and T2 (15 months); 14 of these were HIV positive. At the end of the planned implementation period, the remaining participants (*n=*82) again underwent a process of informed consent and completed the same structured survey instrument. At this time period, T2, 60 participants presented for a second session of biological testing for HIV, hepatitis B and syphilis. Whereas at T1, a partner organization was able to perform biological testing on-site immediately following participant surveys, this coordination was not possible at T2. Participants were required to make an additional visit to a clinic for collection of biological samples; 22 participants were unable or unwilling to conduct this additional visit because of inability to pay transportation costs or other competing issues. Retention support was provided by Enda Santé staff through regular visits or phone calls throughout the follow up period, depending on the wishes of the individual.

### Ethics

All human subjects’ research conducted in accordance with this study has been reviewed and approved by the Senegalese National Ethics Committee for Health Research.

### Analytic approaches

The collected data were linked using anonymous codes. Survey data were entered into SPSS, and monitoring data were collected utilizing Microsoft Excel. All data collected were cleaned and merged into a single database. Inconsistencies found during the data cleaning were reconciled to the original questionnaires or laboratory forms.

These data were analyzed using STATA Version 12 (College Station, Texas). Preliminary analysis was conducted using chi square analysis to determine potential associations of social capital at baseline. HIV incidence was calculated by dividing the number of people who seroconverted between T1 and T2 by the number of participants at risk of HIV acquisition (tested negative at T1 and returned for testing at T2), and multiplying this number by person-time. Because of the small sample size and high rate of loss to follow-up, multivariate regression models were not used.

## Results

The cohort consisted of 119 male participants who reported having anal sex with another man in the past 12 months, with ages ranging from 18 to 42 years. The mean age for all participants was 28 years, with half of the participants between the ages of 23 and 32 years. Those who were found to be HIV-infected were older than those who tested HIV negative (*p=*0.05), with an average age of 28.8 (interquartile range: 25, 32), compared to HIV-uninfected MSM who had an average age of 26.5 (interquartile range: 22, 29). All had had some contact with community groups of MSM in Dakar, Senegal. One-third of the participants had a primary school education or less (*n=*43, 36.4%), one-third had attended secondary school (*n=*39, 33.1%), 15.3% (*n=*18) had attended university and an equal percentage (*n=*18, 15.3%) had attended Islamic or Arab schools. A large majority of participants were single (*n=*104, 88.1%), and 77.3% reported living with their family (*n=*92). Table 1 summarizes the demographic, behavioural, social and financial characteristics of the cohort.

**Table 1:** Baseline demographics and other cohort characteristics

	T1	
	
	*n*	%
*N*	119	–
Demographic variables		
Ethnicity		
Wolof	61	48.7
Other	58	48.7
Education		
Primary or less	43	36.4
Secondary	39	33.1
University	18	15.3
Islamic/Arab School	18	15.3
Marital status		
Single	104	88.1
Married (one wife)	9	7.6
Divorced/ separated	5	4.2
Lives with family	92	77.3
Risk variables		
Always uses condom	102	87.1
Always uses condom and water-based lubricant	75	65.2
Has concurrent partnerships with women	91	76.5
Has ever paid for sex	33	27.7
Has had sex for money	60	50.0
Health variables		
Previously tested HIV-positive	20	17.1
Received STI diagnosis at T1	59	49.2
Ever consulted for an STI	54	46.2
Ever tested for HIV	103	88.0
Ever tested for HIV and received results	91	76.5
Social variables		
Relationship with family		
Excellent	51	42.9
Good	41	34.5
OK	20	16.8
Bad	4	3.4
Very bad	3	2.5
Involved in family decisions	92	78.0
Age of first sex with another man		
12 or younger	35	29.4
13–16	26	21.9
17–19	33	27.7
20 or older	25	21.0
Has a confidant	70	60.8
Financial variables		
Can meet all expenses	28	27.2
Has previously received financial support from HIV project	18	15.1
HIV project		
Has professional qualification	57	71.3
Currently has a job	55	68.8

### Retention results

Thirty-seven of 119 participants were lost to follow-up (31.1%), meaning they were unable or unwilling to participate in the study at T2. Fourteen of those lost to follow up were known to be HIV positive. HIV-positive participants were not lost at a significantly different rate than HIV-negative participants (*p=*0.43). No statistically significant differences were found between those lost to follow-up and those retained to Time 2 comparing any of the variables listed in Table 1.

Reasons for loss to follow up include participant death, participants being unreachable via contact information and social networks, or participants having moved outside of Dakar. Of the participants lost to follow up, four are known to have died (4/119, 0.03). Cause of death was not recorded in this study.

### Social capital results

Analysis of social capital was completed using baseline data. Participants reported having contact with a median of two family members. On average, study participants reported being able to meet about half of their living expenses. Seventy participants (60.8%) reported having a confidant, someone to confide in about matters concerning health, emotional distress and sex.

Men who reported having a confidant were less likely to be HIV positive (OR 0.36, *p <* 0.05, 95% confidence interval [CI] 0.13, 0.97). These men were also less likely to report alcohol use (OR 0.22, *p <* 0.01, 95% CI 0.078, 0.64). Men who reported having a confidant and men who are able to meet their expenses were more likely to report using a condom and water-based lubricant at each anal sex act (for confidant: OR 2.50, *p <* 0.05, 95% CI 1.13, 5.51; for expenses: OR 5.11, *p <* 0.05, 95% CI 1.4–19.2). Further results are reported in Table 2.

**Table 2:** Odds of reporting consistent use of condom and water-based lubricant by social capital indicator

	Condom + lubricant use		
	
Social capital indicator	OR	95% CI	*p*
Three or more sex partners each month	0.44	(0.20, 0.98)	0.04
Believes in MSM collective efficacy	0.42	(0.19, 0.91)	0.03
Current job satisfies needs	7.40	(2.69, 20.37)	0.00
Can meet all expenses (compared with those who can meet no expenses)	5.11	(1.36, 19.16)	0.02
Above median ability to meet expenses	2.83	(1.28, 6.24)	0.01
Ever consulted medical care for an STI	0.32	(0.14, 0.75)	0.01

### Biological results

At the baseline medical examination, 49.2% (*n=*59) of participants were diagnosed with an STI. In the biological testing, three cases of syphilis were diagnosed at baseline (prevalence=2.6%), and two cases were diagnosed at follow-up (prevalence=3.3%). Forty-one participants tested HIV-positive at baseline (36.0%). All participants returning for biological testing at T2 were tested for HIV, regardless of prior test results. Sixty-one participants were tested for at T2, 40 of whom had tested negative at baseline. Eight new infections were observed at T2 (15 months follow up), equating to an annualized incidence of 16 cases per 100 person-years (95% CI 4.6–27.4%) (Table 3).

**Table 3:** Observed prevalence and incidence of HIV and STI

	HIV			Syphilis			Hepatitis B		
			
Time	*N*	Frequency	%	*N*	Frequency	%	*N*	Frequency	%
Prevalence									
T1	114	41	36.0	115	3	2.6	115	16	13.9
T2	61	28	45.9	61	2	3.3	61	11	18.0
Cumulative	108	51	47.2	115	5	4.4	115	18	15.7
Incidence (per person-year)									
–	40	8	0.16	61	2	0.03			

## Discussion

This study attempted to use a community based approach to accrue and retain MSM in Senegal for 15 months while implementing a pilot intervention. Although this study was focused on assessing the feasibility of HIV prevention studies, these data also highlight HIV among MSM as an ongoing public health emergency in Senegal. The high incidence of HIV suggests that this is an ideal population in which to assess novel approaches to prevent HIV acquisition. Moreover, the high prevalence of HIV indicates that this is also an ideal population in which to assess the effectiveness of approaches that address the needs of people living with HIV. These approaches would aim to reduce viral load as a means of improving the health of PLHIV, as well as decreasing the risk of onward HIV transmission.

Loss to follow up in this study was significant, which poses a challenge to the success of future HIV prevention research among MSM in Senegal. Reasons for the loss to follow up were likely multifactorial, including the fact that limited resources were appropriated for enhanced retention approaches in this study. In addition, there was a surprisingly high mortality among this group of men that, with a mean age of 28, was relatively young. Although cause of death was not recorded, anecdotal discussions with community members suggested that these deaths were HIV-related. This pilot cohort study leveraged community groups to implement the study rather than academic teams with significant experience in managing cohorts. Thus, the study demonstrates that cohorts are possible using this approach, but that participant retention strategies should be more thoroughly incorporated into the research protocol. Further research, including qualitative research, is needed to better understand characteristics associated with being retained in the study, and there is a need to explore appropriate retention strategies, for example, using linked peer navigators or SMS-based appointment reminder systems.

Traditional HIV prevention interventions, including condom promotion and HIV testing are necessary. But data on the high force of HIV acquisition and transmission among MSM, as well as the high incidence presented here, suggest that these interventions alone are not enough [43]. Addressing the needs of people at high risk for HIV acquisition could be achieved by assessing the feasibility of antiviral-driven measures such as topical or oral chemoprophylaxis. There are currently Phase II rectal microbicide studies for MSM which include a site in South Africa, and these may eventually represent an important strategy [44]. Separately, oral pre-exposure prophylaxis has been shown to be effective among MSM and may represent a relevant strategy for particularly high-risk men with limited condom usage despite exposure to condom promotion programmes [45]. The proportion of participants in this cohort who had previously been tested for HIV was high, 88%, though many had not received their results. This suggests the need to optimize the continuum of HIV care in this population; this should include ensuring that people are first aware of their HIV status, then assessed for treatment eligibility, actively linked to treatment services and provided with adherence support to achieve viral suppression [46].

Given the high prevalence and incidence of HIV, these data suggest the need to evaluate active linkage to care interventions for MSM in Senegal [47]. A recent systematic review of linkage and utilization of HIV medical care among PLHIV in the United States reported several approaches for linkage to care may be efficacious, including counseling, education and health system navigators [48]. This study was comprised of a highly selected and relatively small sample of MSM already linked into community based organizations in Dakar. However, these men are subject to multiple levels of stigma and discrimination, including exclusion from social activities, isolation from broader social networks and a society that has criminalized their behaviour. Thus, effective HIV intervention packages should address the individual biological and behavioural facilitators of HIV acquisition and transmission, but also address the broader structural determinants of HIV affecting these men.

The baseline data suggests a relationship between social capital and HIV risk including sexual practices and, potentially, prevalent HIV infections. Men who had less financial need were significantly more likely to report use of condoms and water-based lubricant. These results are consistent with earlier data from Senegal noting the importance of financial stability integration of social services as part of health services in the country [49]. These data link social capital to HIV-related risks and suggest that addressing sexual risk practices without addressing the social contexts in which they are taking place may have limited benefit [43]. Documentation and anecdotal reports from the past two decades have suggested that the implementation of interventions that address social capital among MSM can potentially effectively decrease marginalization, stigma and the risk for HIV infection [16,17,50–53]. Although the relationship between social capital and HIV risk is complex, increasing trust and community involvement among this vulnerable population may lead to positive changes in social norms and self-efficacy, and can ultimately lead to lower HIV acquisition and transmission risks [16,17,22,23,26,54,55].

The generalizability of this study to the general population of MSM in Senegal is limited for several reasons. Because of the difficulty of contacting MSM, recruitment was conducted using existing community networks allowing for a representative sample of MSM who are members of community organizations in Dakar. However, this approach potentially excluded those who are the most isolated or those who feel the least desire to become involved in the community of MSM. Thus, using a sampling frame derived from members of community based organizations serving MSM potentially selects for a population with higher social capital than average MSM in Senegal. As earlier mentioned, retention in the study was limited, which did not allow for a statistically powered assessment of the benefit of the intervention. Future studies will need to put a heavy focus on participant retention to facilitate evaluation of the tested packages of interventions.

## Conclusions

Moving forward, cohorts of MSM will be needed to characterize the effectiveness of combination HIV prevention approaches in the West African context. The experience of conducting this feasibility cohort study with a pilot financial intervention illustrates the potential feasibility of such studies among MSM in a region where they are known to be at among the highest risk for the acquisition and transmission of HIV.

## References

[1] Chris Beyrer ALW, Walker D, Johns B, Sifakis F, Baral SD (2011). The global HIV epidemics among men who have sex with men.

[2] Pisani E, Caraël M (1999). Acting early to prevent AIDS: the case of Senegal, in UNAIDS best practice collection.

[3] UNAIDS (2012). Report on the global AIDS epidemic.

[4] Measure DHS (2011). Senegal: standard demographic and health survey, 2010–11.

[5] Foley EE, Nguer R (2010). Courting success in HIV/AIDS prevention: the challenges of addressing a concentrated epidemic in Senegal. Afr J AIDS Res.

[6] Niang CI, Tapsoba P, Weiss E, Diagne M, Niang Y, Moreau AM (2003). ‘It's raining stones’: stigma, violence and HIV vulnerability among men who have sex with men in Dakar, Senegal. Cult Health Sex.

[7] Fay H, Baral SD, Trapence G, Motimedi F, Umar E, Iipinge S (2011). Stigma, health care access, and HIV knowledge among men who have sex with men in Malawi, Namibia, and Botswana. AIDS Behav.

[8] Ansari DA, Gaestel A (2010). Senegalese religious leaders’ perceptions of HIV/AIDS and implications for challenging stigma and discrimination. Cult Health Sex.

[9] Preston DB, D'Augelli AR, Cain RE, Kassab CD, Schulze FW, Starks MT (2004). The influence of stigma on the sexual risk behavior of rural men who have sex with men. AIDS Educ Prev.

[10] Wang C, Hawes SE, Gaye A, Sow PS, Ndoye I, Manhart LE (2007). HIV prevalence, previous HIV testing, and condom use with clients and regular partners among Senegalese commercial sex workers. Sex Transm Infect.

[11] Wade AS, Larmarange J, Diop AK, Diop O, Gueye K, Marra A (2010). Reduction in risk-taking behaviors among MSM in Senegal between 2004 and 2007 and prevalence of HIV and other STIs. ELIHoS Project, ANRS 12139. AIDS Care.

[12] Baral S, Burrell E, Scheibe A, Brown B, Beyrer C, Bekker LG (2011). HIV risk and associations of HIV infection among men who have sex with men in Peri-Urban Cape Town, South Africa. BMC Public Health.

[13] Dramé FM, Peitzmeier S, Lopes M, Ndaw M, Sow A, Diouf D (2012). Gay men and other men who have sex with men in West Africa: evidence from the field. Cult Health Sex.

[14] Griensven Fv (2007). Men who have sex with men and their HIV epidemics in Africa. AIDS.

[15] Krishna AS, Shrader E (1999). Social capital assessment tool. Conference on Social Capital and Poverty Reduction.

[16] Bhattacharya G (2005). Social capital and HIV risks among acculturating Asian Indian men in New York City. AIDS Educ Prev.

[17] Campbell C, Williams B, Gilgen D (2002). Is social capital a useful conceptual tool for exploring community level influences on HIV infection? An exploratory case study from South Africa. AIDS Care.

[18] Friedman SR, Mateu-Gelabert P, Curtis R, Maslow C, Bolyard M, Sandoval M (2007). Social capital or networks, negotiations, and norms? A neighborhood case study. Am J Prev Med.

[19] Lindstrom M, Axen E (2004). Social capital, the miniaturization of community and assessment of patient satisfaction in primary healthcare: a population-based study. Scand J Public Health.

[20] Lovell AM (2002). Risking risk: the influence of types of capital and social networks on the injection practices of drug users. Soc Sci Med.

[21] Reid E (2000). Incorporating social capital into development practice: the HIV epidemic. Dev Bull.

[22] Sivaram S, Zelaya C, Srikrishnan AK, Latkin C, Go VF, Solomon S (2009). Associations between social capital and HIV stigma in Chennai, India: considerations for prevention intervention design. AIDS Educ Prev.

[23] Smith RA, Rimal R (2009). The impact of social capital on HIV – related actions as mediated by personal and proxy efficacies in Namibia. AIDS Behav.

[24] Sundquist K, Yang M (2007). Linking social capital and self-rated health: a multilevel analysis of 11,175 men and women in Sweden. Health Place.

[25] Takahashi LM, Magalong MG (2008). Disruptive social capital: (un)healthy socio-spatial interactions among Filipino men living with HIV/AIDS. Health Place.

[26] Wouters E, Meulemans H, van Rensburg HCJ (2009). Slow to share: social capital and its role in public HIV disclosure among public sector ART patients in the Free State province of South Africa. AIDS Care.

[27] Sanders EJ, Graham SM, Okuku HS, van der Elst EM, Muhaari A, Davies A (2007). HIV-1 infection in high risk men who have sex with men in Mombasa, Kenya. AIDS.

[28] Baral S, Trapence G, Motimedi F, Umar E, Iipinge S, Dausab F (2009). HIV prevalence, risks for HIV infection, and human rights among men who have sex with men (MSM) in Malawi, Namibia, and Botswana. PLoS One.

[29] Kelly JA, Amirkhanian YA, McAuliffe TL, Dyatlov RV, Granskaya J, Borodkina OI (2001). HIV risk behavior and risk-related characteristics of young Russian men who exchange sex for money or valuables from other men. AIDS Educ Prev.

[30] Baral S, Kizub D, Masenior NF, Peryskina A, Stachowiak J, Stibich M (2009). Male sex workers in Moscow, Russia: a pilot study of demographics, substance use patterns, and prevalence of HIV-1 and sexually transmitted infections. AIDS Care.

[31] Pronyk PM, Harpham T, Morison LA, Hargreaves JR, Kim JC, Phetla G (2008). Is social capital associated with HIV risk in rural South Africa?. Soc Sci Med.

[32] Cené CW, Akers AY, Lloyd SW, Albritton T, Hammond WP (2011). Understanding social capital and HIV risk in rural African American communities. J Gen Intern Med.

[33] David AC, Li CA (2010). Exploring the links between HIV/AIDS, social capital and development. J Int Dev.

[34] Safren SA, Traeger L, Skeer MR, O'Cleirigh C, Meade CS, Covahey C (2010). Testing a social-cognitive model of HIV transmission risk behaviors in HIV-infected MSM with and without depression. Health Psychol.

[35] Mimiaga MJ, Noonan E, Donnell D, Safren SA, Koenen KC, Gortmaker S (2009). Childhood sexual abuse is highly associated with HIV risk-taking behavior and infection among MSM in the EXPLORE Study. J Acquir Immune Defic Syndr.

[36] Safren SA, Reisner SL, Herrick A, Mimiaga MJ, Stall R (2010). Mental health and HIV risk in men who have sex with men. J Acquir Immune Defic Syndr.

[37] Johnson CA (2007). Off the map how HIV/AIDS programming is failing same-sex practicing people in Africa.

[38] Moreau A (2007). Implementing STI/HIV prevention and care interventions for men who have sex with men in Dakar, Senegal.

[39] Poteat T, Diouf D, Drame FM, Ndaw M, Traore C, Dhaliwal M (2011). HIV risk among MSM in Senegal: a qualitative rapid assessment of the impact of enforcing laws that criminalize same sex practices. PLoS One.

[40] Trapence G, Collins C, Avrett S, Carr R, Sanchez H, Ayala G (2012). From personal survival to public health: community leadership by men who have sex with men in the response to HIV. Lancet.

[41] Gordis L (2009). Epidemiology.

[42] CNLS (2009). Surveillance sentinelle du VIH et de la syphilis auprès des femmes enceintes au Sénégal. Bulletin épidémiologique.

[43] Sullivan PS, Carballo-Diéguez A, Coates T, Goodreau SM, McGowan I, Sanders EJ (2012). Successes and challenges of HIV prevention in men who have sex with men. Lancet.

[44] McGowan I (2011). Rectal microbicides: can we make them and will people use them?. AIDS Behav.

[45] Grant RM, Lama JR, Anderson PL, McMahan V, Liu AY, Vargas L (2010). Preexposure chemoprophylaxis for HIV prevention in men who have sex with men. N Engl J Med.

[46] Gardner EM, McLees MP, Steiner JF, del Rio C, Burman WJ (2011). The spectrum of engagement in HIV care and its relevance to test-and-treat strategies for prevention of HIV infection. Clin Infect Dis.

[47] Christopoulos KA, Das M, Colfax GN (2011). Linkage and retention in HIV care among men who have sex with men in the United States. Clin Infect Dis.

[48] Liau A, Crepaz N, Lyles CM, Higa DH, Mullins MM, DeLuca J (2013). Interventions to promote linkage to and utilization of HIV medical care among HIV-diagnosed persons: a qualitative systematic review, 1996–2011. AIDS Behav.

[49] Enel C, Larmarange J, Desgrées du LA, Wade AS (2009). À propos des partenaires féminines des hommes ayant des pratiques homosexuelles au Sénégal [Regarding feminine partners of Men having homosexuals practices in Senegal]. Autrepart.

[50] Swendeman D, Basu I, Das S, Jana S, Rotheram-Borus MJ (2009). Empowering sex workers in India to reduce vulnerability to HIV and sexually transmitted diseases. Soc Sci Med.

[51] Pinto RM, Melendez RM, Spector AY (2008). Male-to-female transgender individuals building social support and capital from within a gender-focused network. J Gay Lesbian Soc Serv.

[52] Macinko J, Starfield B (2001). The utility of social capital in research on health determinants. Milbank Q.

[53] Amirkhanian YA, Kelly JA, Kabakchieva E, Kirsanova AV, Vassileva S, Takacs J (2005). A randomized social network HIV prevention trial with young men who have sex with men in Russia and Bulgaria. AIDS.

[54] Campbell C, Mzaidume Z (2001). Grassroots participation, peer education, and HIV prevention by sex workers in South Africa. Am J Public Health.

[55] Chiu J, Grobbelaar J, Sikkema K, Vandormoel A, Bomela N, Kershaw T (2008). HIV-related stigma and social capital in South Africa. AIDS Educ Prev.

# Sub article: Outcomes of a community-based HIV-prevention pilot programme for township men who have sex with men in Cape Town, South Africa

**DOI:** 10.7448/IAS.16.4.18754

**Published:** 2013-12-02

**Authors:** Elizabeth Batist, Benjamin Brown, Andrew Scheibe, Stefan D Baral, Linda-Gail Bekker

**Affiliations:** 1 Desmond Tutu HIV Foundation, Cape Town, South Africa; 2 Department of Epidemiology, Johns Hopkins School of Public Health Baltimore, MD, USA

**Keywords:** community-based, self-esteem, stigma, African men who have sex with men (MSM), social network, outreach

## Abstract

**Introduction:**

Men who have sex with men (MSM) in Cape Town's townships remain in need of targeted HIV-prevention services. In 2012, a pilot community-based HIV-prevention programme was implemented that aimed to reach MSM in five Cape Town townships, disseminate HIV-prevention information and supplies, and promote the use of condoms and HIV services.

**Methods:**

Convenience sampling was used to recruit self-identified MSM who were 18 years old or older in five Cape Town townships. The six-month pilot programme trained five community leaders who, along with staff, provided HIV-prevention information and supplies to MSM through small-group meetings, community-based social activities and inter-community events. After the completion of the pilot programme, in-depth interviews and focus group discussions (FGDs) were conducted with a subset of conveniently sampled participants and with each of the community leaders. Qualitative data were then analyzed thematically.

**Results:**

Overall, 98 mostly gay-identified black MSM consented to participate, 57 community-based activities were facilitated and 9 inter-community events were conducted. Following their enrolment, 60% (59/98) of participants attended at least one pilot activity. Of those participants, 47% (28/59) attended at least half of the scheduled activities. A total of 36 participants took part in FGDs, and five in-depth interviews were completed with community leaders. Participants reported gaining access to MSM-specific HIV-prevention information, condoms and water-based lubricant through the small-group meetings. Some participants described how their feelings of loneliness, social isolation, self-esteem and self-efficacy were improved after taking part.

**Conclusions:**

The social activities and group meetings were viable strategies for disseminating HIV-prevention information, condoms and water-based lubricant to MSM in this setting. Many MSM were also able to receive social support, reduce social isolation and improve their self-esteem. Further research is needed to explore factors affecting attendance and the sustainability of these activities. Perspectives of MSM who did not attend pilot activities regularly were not equally represented in the final qualitative interviews, which could bias the findings. The use of community-based activities and small-group meetings should be explored further as components to ongoing HIV-prevention interventions for MSM in this setting.

## Introduction

Men who have sex with men (MSM) remain significantly affected by HIV in South Africa, with a reported HIV prevalence between 10 and 50% [1–6]. MSM risk is affected by many individual and structural factors, including unprotected anal intercourse (UAI), substance use and discriminatory healthcare [2–13]. Although MSM-competent HIV-prevention services are expanding across the country, there are still several gaps [1,14–17]. Reaching MSM with these services is critical for their well-being and is considered essential in addressing the broader HIV epidemic in South Africa [1,6,10,18].

Community-based approaches have been used to reach MSM and other marginalized populations with HIV-prevention services in many settings [18–21]. These programmes utilize peer education and the facilitation of safe social spaces to provide HIV education, address stigma, address behavioural risks and link individuals into HIV testing or care [18–25]. Similar strategies have been used to reach Southern African MSM with HIV research, HIV-prevention information, and HIV counselling and testing [26–32].

In South Africa, MSM-specific service providers and non-governmental organizations (NGOs), including the Desmond Tutu HIV Foundation (DTHF), engage MSM through both peer education and the use of safe spaces within township communities [13,16,30,33,34]. In 2008, the DTHF used several of these strategies to recruit MSM in Cape Town for the Global iPrEX study, a biomedical HIV-prevention clinical trial [29,35]. MSM social networks in Cape Town's townships have been described as including key individuals who establish spaces where other MSM are able to socialize safely [36]. As a result of the iPrEX study, links with these individuals and multiple MSM social networks were formed. This led the DTHF to design and conduct a pilot community-based HIV-prevention programme with MSM in these networks.

This pilot programme aimed to reach MSM in various townships through the use of community-based social activities and meeting groups. The programme was designed to disseminate HIV-prevention information and supplies, and promote the use of condoms and HIV service uptake. This article presents an overview of the project methods, a description of the participants and results from follow-up interviews and focus group discussions (FGDs) conducted with a subset of participants.

## Methods

The pilot HIV prevention programme was implemented with MSM in five predominantly black African townships in greater Cape Town. Three structured components were included: (i) group meetings were held regularly with small gatherings of MSM to facilitate knowledge exchange and disseminate prevention supplies, (ii) community-based activities were facilitated to provide opportunities for MSM group bonding and (iii) inter-community activities were conducted to promote integration and diversity. All pilot activities took place over a six-month period between May and October 2012.

### Community leader selection and participant recruitment

Townships were selected based on high HIV prevalence, which was identified through previous HIV surveillance data, and on the presence of MSM social networks identified through recruitment for the Global iPrEX study.

From each township, one MSM community leader was identified from previous research [30]. Community leaders participated in the planning and facilitation of all activities, disseminated HIV-prevention information and provided healthcare referrals to MSM in their community. They were at least 18 years old; had demonstrated leadership qualities; were respected, trusted and socially prominent among their MSM peers; and lived in a township where pilot activities were planned. The initial community leader team was selected and trained between January and April 2012.

Self-identified MSM were then recruited to take part in the pilot programme using convenience sampling through peer outreach workers and venue-based contact. All participants were 18 years old or older, were born male, were reported to have sex with men and lived in a township where the pilot was taking place. Each participant completed a self-administered paper questionnaire that collected baseline data on their demographic characteristics, sexual practices, health-seeking behaviour and access to services. Participants were offered voluntary HIV counselling and testing by trained staff and were provided with information about MSM-competent healthcare facilities. Participants who tested HIV positive were provided counselling and referrals. Participant recruitment was completed in 57 days between May and July 2012.

### Implementation of the pilot programme

Community leaders received initial two-day training and completed follow-up trainings throughout the pilot. Trainings included education on sexually transmitted infections and HIV but primarily focused on developing leadership skills such as effective communication, managing complicated social situations, strategic planning and goal setting, and encouraging healthy social norms.

Group meetings took place every 1–2 weeks and were held in private and safe venues in each township. Meetings were semi-structured and included both social and educational components such as debates about current events, training on condoms and water-based lubricant, and discussions on HIV-prevention strategies. Meetings were facilitated by a community leader and staff member but guided mostly by the participants, who were encouraged to take ownership and direction of each meeting. Condoms, water-based lubricant and HIV-prevention information were disseminated during these meetings.

Community-based activities were designed based on participant feedback and used to supplement group meetings in each township. Community-based activities included sports (hiking, netball and soccer), dance competitions, drag pageants and debates. Similar to group meetings, HIV-prevention discussions were integrated into each of the activities. Light refreshments were provided to participants at all meetings and activities.

Finally, inter-community activities, which brought together at least two different MSM groups, were conducted at least once a month. These activities were similar in scope to the community-based activities but were organized to promote knowledge sharing and socializing between MSM from different townships. MSM participants were provided with transport to attend inter-community activities.

## Data collection and analysis

### Quantitative methods

Quantitative data from the baseline questionnaires were analyzed using STATA version 11.0 (StataCorp LP, College Station, TX). Numerical variables were explored using measures of central tendency and distribution [medians and interquartile ranges (IQRs)], and categorical variables were explored using proportions and frequency tables.

Participants were requested to sign an attendance register at each activity. Registers were entered into a secure Excel spreadsheet and linked to the participant's ID. Attendance was measured for each participant and defined as the total number of events attended by the total offered to that participant.

### Qualitative methods

After completion of the pilot activities, IDIs with each of the community leaders and FGDs with a subset of participants were conducted in December 2012. A purposive sampling strategy was initially used to equally represent MSM who attended regularly and those who did not. However, many participants who did not attend regularly were unable to be contacted, resulting in the remaining FGD slots being filled by participants who attended more frequently.

All FGDs and one IDI were conducted in private facilities within each community, and four IDIs were conducted at the research offices of the DTHF. All FGDs and IDIs were conducted by one of the two trained facilitators and supported by a research assistant who took notes. The FGDs and IDIs were conducted predominantly in English, but participants were also encouraged to use the language they felt most comfortable speaking. A semi-structured interview guide was used to explore participants’ perceptions and experiences with community life, project activities, stigma, healthcare services and HIV.

Audio recordings from each FGD and IDI were transcribed, and all participant-identifying information was removed. Qualitative data were analyzed using the framework approach. Predetermined themes based on the interview guide questions were used to structure the initial framework, and a coding scheme was developed to identify emerging themes. Two analysts reviewed transcripts from one FGD and IDI together to establish consistency in coding. After this, the analysts each reviewed the remainder of the transcripts individually. Comparisons and discussion between analysts were used to reach consensus on final themes.

#### Ethical consideration

Written informed consent was obtained from all participants, who were reminded that they would be able to take part in any community-based activities regardless of their decision to participate in this pilot study. Participants taking part in the follow-up FGDs and IDIs were informed that their responses would remain anonymous and would not affect their involvement in future initiatives from the DTHF or other organizations. They received R50 (approximately US$5.00) as reimbursement for their time and transport. Community leaders were provided with a monthly stipend of R800 (approximately US$90.00) as compensation for transport costs and their time spent in project activity planning and implementation. Ethical approval for this project was obtained from the University of Cape Town's Faculty of Health Sciences Human Research Ethics Committee.

## Results

### Participant baseline characteristics

In total, 98 MSM consented to participate and completed a baseline questionnaire. The majority of participants were black African (95%, 93/98) and gay identified (82.3%, 79/96). The median age of participants was 24.5 with an IQR of 21–29. Over half of the participants had received secondary education (64.3%, 63/98), and less than one-third (28.6%, 28/98) reported current employment. High-risk sexual behaviours including UAI and transactional sex were reported by MSM in each community. In total, 26% (25/98) of participants reported having had at least one female sexual partner in the last six months. A summary of participant baseline characteristics is presented in Table 1.

**Table 1:** Participant baseline characteristics

Variable	Community A	Community B	Community C	Community D	Community E	Total
Total enrolled	33	14	24	17	10	98
Age median (IQR)	22 (19–25)	23 (20–27)	29.5 (24.5–36)	26 (24–28)	25 (21–31)	24.5 (21–29)
Race						
Black	97% (32/33)	100% (14/14)	83% (20/22)	100% (17/17)	80% (8/10)	95% (93/98)
Coloured	3% (1/33)	0%	17% (4/22)	0%	10% (1/10)	6% (6/98)
White	0%	0%	0%	0%	10% (1/10)	1% (1/98)
Sexual orientation						
Gay	78.8% (26/33)	100% (14/14)	77% (17/22)	77% (13/17)	90% (9/10)	82.3% (79/96)
Bisexual	3.0% (1/33)	0%	22% (5/22)	17.6% (3/17)	10% (1/10)	10.4% (10/96)
Straight	18.2% (6/33)	0%	0%	5.8% (1/17)	0%	7.3% (7/96)
Currently employed	24.2% (8/33)	28.6% (4/14)	20.8% (5/24)	35.2% (6/17)	50% (5/10)	28.6% (28/98)
Education						
Primary	3% (1/33)	0%	4% (1/24)	0%	10% (1/10)	3% (3/98)
Secondary	60.6% (20/33)	50% (7/14)	79% (19/24)	53% (9/17)	80% (8/10)	64.3% (63/98)
Tertiary	36.4% (12/33)	50% (7/14)	17% (4/24)	47% (8/17)	10% (1/10)	33% (32/98)
Number of male partners in last year, median (IQR)	9 (2–15)	5.5 (3–6)	6 (2.5–15)	3 (2–5)	1 (1–3)	5 (2–10)
Number of female partners in last six months, median (IQR)	2 (1–2)	1 (1–1)	2.5 (2–13)	1.5 (1–3.5)	4.5 (2–7)	2 (1–3)
Number of male partners had UAI with in the last six months, median (IQR)	2.5 (1–6)	1 (1–2)	2 (1–6)	1 (1–2)	1.5 (1–2)	2 (1–4)
Ever-reported STI	18.8% (6/32)	61.5% (8/13)	37.5% (9/25)	35.3% (6/17)	20% (2/10)	32.3% (31/96)
Transactional sex						
Paid for	24.2% (8/33)	0%	20.8% (5/24)	23.5% (4/17)	20% (2/10)	19% (19/98)
Received	24.2% (8/33)	15.4% (2/13)	33% (8/24)	19% (3/16)	20% (2/10)	24% (23/98)
Age of sexual debut, median (IQR)	15 (14–16)	17 (14–18)	17 (15–19)	17.5 (16–20)	18 (16–19)	16 (15–19)
Ever tested for HIV	90.9% (30/33)	92.8% (13/14)	100% (23/23)	94.4% (16/17)	90.0% (9/10)	93.8% (91/97)
Months since last HIV test, median (IQR)	5 (2–12)	3 (1–6)	6 (2–12)	2 (0.88–6)	7 (7–10)	4 (2–12)
Months since last visit to local clinic, median (IQR)	3 (2–6.5)	2 (1–3)	2 (1–4)	2 (0.75–5.5)	4 (.25–12)	2 (1–6)
Disclosed orientation to a healthcare worker	45.5% (15/33)	84.6% (11/13)	65.2% (15/23)	70.6% (12/17)	60% (6/10)	61.5% (59/96)
Ever visited an MSM-friendly clinic	21.2% (7/33)	100% (14/14)	70.8% (17/24)	47.1% (8/17)	30% (3/10)	50% (49/98)
Communication						
Owns a cell phone	93.9% (31/33)	92.9% (13/14)	83.3% (20/24)	100% (17/17)	90% (9/10)	91.8% (90/98)
Regular access to the internet	69.7% (23/33)	85.7% (12/14)	41.7% (10/24)	82.4% (14/17)	80% (8/10)	68.4% (67/98)
Sexual partner contact						
Via a cell phone chat programme	63.6% (21/33)	57.1% (8/14)	68% (17/25)	72.2% (13/18)	70% (7/10)	66% (66/98)
Via the internet	57.9% (19/33)	64.3% (9/14)	58.3% (14/24)	64.7% (11/17)	40% (4/10)	58% (57/98)

IQR: interquartile range; UAI: unprotected anal intercourse; STI: sexually transmitted infection; MSM: men who have sex with men.

### Community activities

MSM community groups were established in 5 townships, and 57 community-based activities including group meetings and 9 inter-community activities were conducted between May and October 2012. Participant enrolment varied between communities, with 33 participants enrolled from Community A, 24 from Community C, 17 from Community D, 14 from Community B and 10 from Community E. Less than half of the participants (44%, 43/98) had previously engaged in other MSM-focused activities or research prior to the pilot.

Attendance registers were not collected from 7 of the 57 community meetings and from one of the inter-community events due to an administrative error. A median of eight (IQR 6–9) MSM attended the 50 community meetings with attendance registers, and the eight inter-community activities were attended by a median of 20 (IQR 19.25–21.5) MSM. Condoms and lubricants were distributed during 23 activities and were available on request throughout the duration of the project. Following their enrolment, 60% (59/98) of participants attended at least one pilot activity. Of those participants, 47% (28/59) attended at least one-half of the scheduled activities. A summary of attendance is shown in Table 2.

**Table 2:** Event attendance per community

Percentage of events attended	Community 1 participants	Community 2 participants	Community 3 participants	Community 4 participants	Community 5 participants	Total
0	16	4	9	5	5	39
1–25	5	4	5	4	0	28
26–50	4	2	5	2	0	13
51–75	3	1	4	2	5	15
76–100	5	3	1	4	0	13

#### Follow-up interviews and focus group discussions

Of the 100 MSM who took part in the pilot activities, 36 also participated in follow-up FGDs, and each of the five community leaders completed an IDI. Efforts were made to include participants with varying degrees of attendance; however, there was substantial loss to follow-up of the participants who had lower attendance. Overall, more than half of the participants from the FGDs attended 50% or more of the scheduled activities.

#### HIV knowledge, testing and services

Many participants described the benefits of receiving MSM-specific HIV-prevention knowledge through the meeting groups, while others reported having already received this information elsewhere:

I didn't know everything about preventing HIV or AIDS but once I joined the group I've got more information and then that information I used it …. I was worried at first you know until I joined the group and then it influenced me in a kinda way to be strong don't have to be worried since you know that mhm theres so many things which can protect you from getting HIV. (FGD1)

Participant attitudes towards HIV testing at local health clinics remained consistently negative because of the insensitive or discriminatory care many had previously received. Despite reactions to local healthcare clinics, participants were aware and made use of MSM-competent healthcare services throughout the duration of the pilot project.

#### Use of water-based lubricants

Prior to the pilot, participants reported limited access to free water-based lubricant and described using petroleum-based lubricant during anal sex. Many participants described that their use of condoms remained inconsistent, particularly with regular sexual partners. Other participants continually referred to an improved knowledge and use of water-based lubricant as a result of taking part in the pilot activities, specifically the group meetings:

But we came to the group and they taught us that you have to use specific lube … before we can have sex. (FGD 2)

#### Social support and personal development

Participants explained how their feelings of loneliness and social isolation were improved after taking part in the pilot because it created opportunities to socialize with other MSM. This seemed particularly true in communities with little existing MSM activities.

… You are able to, you know, be yourself and the sense of getting to be yourself and also giving the feeling that you are not alone …. (FGD 1)

… It's nice when we had events, especially in our communities, because there's nothing happening so much in our community. Especially for gay people […] So the moment there is an event some people think that's where they come out to explore, “okay, I'm not the only one who's gay” …. (FGD 4)

Participants detailed how they gained meaningful social support from their peers during group meetings in each township.

When you are having this, such groups, we encourage each other, we talk to each other, we giving each other advice. So it's quite good. Whatever problem you experience in life, if you share with someone, it does help. (FGD 4)

So sometime getting together as township gay moffies, it builds us … sometimes you need to share your story with someone. That is, who will understand who you are, you understand? Because sometimes you talk to a person who's, who's a stranger in LGBTI. It doesn't work. There's no use of that because that person will look at you as you are out of your mind. (FGD 4)

Many participants shared how their self-esteem and self-efficacy improved during the meeting groups and community-based activities. For example, participants in multiple townships noted that they were able to explore and understand their sexuality, some for the first time:

I have also grown and became quite content with who I am. Cause at first, before I joined the group, I was one of those people who were in the closet as many of us would know. And […] as the time went by, I began [to be] interested in to finding out more about who I am, and why am I gay. (FGD 3)

It really did help, like for me or like, for me my family. They're religious people and they hated the fact that am gay but then I've started coming to the meetings and then I … I had the guts to tell them that now am gay …. (FGD 1)

#### Stigma

Overall, participant opinions about the role that the pilot programme played in addressing stigma were mixed, but some participants felt that the pilot activities allowed MSM to gain greater visibility in their communities.

There were outdoor events. So everybody who was even passing when we doing these kinds of activities [outdoor sporting event] were like “oh my god, this is quite interesting.” Guys playing netball, you know? […] we are trying to show the community that we are there. (FGD 3)

Participants also described how group meetings helped them to better prepare for and mitigate the effects of stigma and prejudice.

… People would like to ask question “why I'm gay?” and “why I'm doing this?” … So I'm sure this meeting helped me a lot, I mean, to get through - those kind of answers, you know what I mean? (FGD2)

If you tell your story, you talk to people then … [exhales] … it's a burden that you take off your shoulders …. (FGD 2)

#### Suggestions to improve HIV programme implementation

Participants offered suggestions for improving the implementation of the pilot activities. Specifically, they felt that staff changes should be kept minimal since it was challenging to develop relationships with new outreach staff. Some participants also expressed the need for improved efficiency with inter-community activities, specifically highlighting the transport and timeliness of other MSM as key barriers. Overall, participants also shared a willingness to engage their broader community to address stigma and expressed a need for activities to do so by targeting other community members beyond MSM.

## Discussion

This article presents the outcomes of a pilot community-based HIV-prevention programme for township MSM. It is important to note that while participants described changes in their behaviour as a result of the pilot, its aim was not to measure behaviour change. Many factors, including concurrent programmes, may have influenced participants’ behaviour [14,15]. Taking this into consideration, participant responses do suggest that this pilot was successful in achieving some of its primary objectives.

First, the pilot programme successfully engaged MSM from high-risk networks in five Cape Town townships. Attendance data suggest that social activities and group meetings were a feasible method for reaching certain MSM in this pilot; however, overall attendance varied greatly and included a large percentage of participants who attended no activities. This variability may suggest that the pilot activities did not cater to the interests or needs of all participants, particularly the unique needs of MSM [37]. Other factors that may influence attendance have been described and should be explored further in this context, including feelings of mistrust and community stigma [33,38].

Second, HIV-prevention information and supplies were successfully disseminated to MSM during this pilot. Other studies have described how facilitated social spaces can result in knowledge gain by encouraging the exchange and processing of information between peers [39]. Similarly, MSM in this pilot felt that group meetings created safe environments to learn about HIV prevention with their peers. In addition to improving knowledge, increasing access to water-based lubricants and condoms is also essential for MSM, particularly in communities where limited or incorrect lubricant use has been reported [2]. These findings support previous suggestions to explore the use of small community-based spaces for lubricant dissemination [40]. Small meeting groups and social activities should be further explored as strategies to supplement current lubricant dissemination strategies for MSM in this setting.

Third, participants reported other meaningful benefits to this pilot, including improvements in their self-efficacy, self-esteem and social isolation. Social isolation, poor self-efficacy and limited social support may play important roles in the individual risk of MSM, specifically condom negotiation and lubricant use [10,33,41,42]. Since this study did not aim to address social isolation or self-efficacy directly, it remains to be seen if any risk reduction occurred through this pilot as a result of diminished social isolation or improvements in self-efficacy. However, these results do support previous recommendations to further explore self-efficacy with township MSM in HIV-prevention programmes, and they suggest that community-based group meetings and social activities warrant further investigation as feasible methods to do so [41].

Additional research is needed to explore community-based approaches for condom use and HIV testing in this setting. HIV testing and condom use are complex behaviours affected by a multitude of factors, including stigma [10,43]. MSM in this pilot were supportive of broader community interventions to reduce stigma, lending further support to current recommendations for future community-based HIV-prevention interventions to explore methods that empower MSM to safely and appropriately address stigma within their communities [40].

There are limitations to this pilot study. This pilot targeted black African townships; therefore, these findings cannot be extrapolated to other groups. MSM who did not attend pilot activities were not equally represented in the final qualitative interviews. Their reasons for non-participation may not be adequately included in these findings. Even though participants openly shared suggestions for improving the programme, their responses may have been biased towards discussing positive benefits of the programme in general. The timeframe of this pilot was brief and cannot address the sustainability of these activities in the long term.

Taking these limitations into consideration, this community-based HIV-prevention pilot programme provides useful insights for MSM-specific HIV-prevention programming that warrant further research. Specifically, small meeting groups and social activities promoted an enabling environment, within the context of larger stigmatizing communities, where MSM were able to receive social support, improve their self-esteem and gain access to relevant HIV-prevention information and supplies.

## Conclusions

Results from this pilot programme describe how township-based MSM can benefit from facilitated social activities and meeting groups. Results from this pilot programme suggest that these strategies are a viable method for disseminating HIV-prevention information, condoms and water-based lubricant. Furthermore, these groups create a supportive environment in which MSM can learn from each other, explore their sexual identities and overcome potential barriers to HIV prevention such as social isolation and low self-esteem. The use of community-based social activities and facilitated small-group meetings should be furthered explored as components to ongoing HIV-prevention interventions for MSM in this setting.

## References

[1] Council SANA (2012). National strategic plan on HIV, STIs and TB 2012–2016.

[2] Baral S, Burrell E, Scheibe A, Brown B, Beyrer C, Bekker LG (2011). HIV risk and associations of HIV infection among men who have sex with men in peri-urban Cape Town, South Africa. BMC Public Health.

[3] Burrell E, Mark D, Grant R, Wood R, Bekker L-G (2010). Sexual risk behaviours and HIV-1 prevalence among urban men who have sex with men in Cape Town, South Africa. Sex Health.

[4] Lane T, Raymond HF, Dladla S, Rasethe J, Struthers H, McFarland W (2011). High HIV prevalence among men who have sex with men in Soweto, South Africa: results from the Soweto Men's Study. AIDS Behav.

[5] Rispel LC (2011). HIV prevalence and risk practices among men who have sex with men in two South African cities. J Acquir Immune Defic Syndr.

[6] Sandfort TG, Nel J, Rich E, Reddy V, Yi H (2008). HIV testing and self-reported HIV status in South African men who have sex with men: results from a community-based survey. Sex Transm Infect.

[7] Lane T, Shade SB, McIntyre J, Morin SF (2008). Alcohol and sexual risk behavior among men who have sex with men in South African township communities. AIDS Behav.

[8] Rispel LC, Metcalf CA, Cloete A, Reddy V, Lombard C (2011). HIV prevalence and risk practices among men who have sex with men in two South African cities. J Acquir Immune Defic Syndr.

[9] Dunkle KL, Jewkes RK, Murdock DW, Sikweyiya Y, Morrell R (2013). Prevalence of consensual male-male sex and sexual violence, and associations with HIV in South Africa: a population-based cross-sectional study. PLoS Med.

[10] Jobson G, de Swardt G, Rebe K, Struthers H, McIntyre J (2013). HIV risk and prevention among men who have sex with men (MSM) in peri-urban townships in Cape Town, South Africa. AIDS Behav.

[11] Knox J, Sandfort T, Yi H, Reddy V, Maimane S (2011). Social vulnerability and HIV testing among South African men who have sex with men. Int J STD AIDS.

[12] Lane T, Mogale T, Struthers H, McIntyre J, Kegeles SM (2008). “They see you as a different thing”: the experiences of men who have sex with men with healthcare workers in South African township communities. Sex Transm Infect.

[13] Scheibe A, Brown B, Duby Z, Bekker LG (2011). Key populations, key solutions: a gap analysis for key populations and HIV in South Africa, and recommendations for the National Strategic Plan for HIV/AIDS, STIs and TB (2012–2016). Desmond Tutu HIV Foundation, Joint UN Team on HIV and AIDS.

[14] McIntyre JA, Struthers H (2013). HIV and men who have sex with men in South Africa. AIDS Behav.

[15] Rebe K, Swardt GD, Struthers H, McIntyre J (2012). Towards ‘men who have sex with men- appropriate’ health services in South Africa. Southern African Journal of HIV Medicine.

[16] Africa IS (2012). Rapid situational assessment report: HIV prevention services for men who have sex with men and capacity building assistance for high risk, underserved populations in South Africa: The Eastern Cape, Free State, Kwazulu-Natal, Limpopo & Northern Cape Provinces.

[17] (2012). Technical Working group. Operational guidelines for HIV, STI, and TB programmes for key populations in South Africa.

[18] Trapence G, Collins C, Avrett S, Carr R, Sanchez H, Ayala G (2012). From personal survival to public health: community leadership by men who have sex with men in the response to HIV. Lancet.

[19] Kegeles SM, Hays RB, Coates TJ (1996). The Mpowerment Project: a community-level HIV prevention intervention for young gay men. Am J Public Health.

[20] Jana S, Basu I, Rotheram-Borus MJ, Newman PA (2004). The Sonagachi Project: a sustainable community intervention program. AIDS Educ Prev.

[21] Campbell C, Scott K, Nhamo M, Nyamukapa C, Madanhire C, Skovdal M (2013). Social capital and HIV competent communities: the role of community groups in managing HIV/AIDS in rural Zimbabwe. AIDS Care.

[22] Beyrer C (2010). Global prevention of HIV infection for neglected populations: men who have sex with men. Clin Infect Dis.

[23] amfAR M (2010). Lessons from the front lines: effective community-led responses to HIV and AIDS among MSM and transgender populations.

[24] Campbell C, Nhamo M, Scott K, Madanhire C, Nyamukapa C, Skovdal M (2013). The role of community conversations in facilitating local HIV competence: case study from rural Zimbabwe. BMC Public Health.

[25] Cornish F, Campbell C (2009). The social conditions for successful peer education: a comparison of two HIV prevention programs run by sex workers in India and South Africa. Am J Community Psychol.

[26] Beyrer C, Wirtz A, Walker D, Johns B, Sifakis F, Baral S (2011). The global HIV epidemics among men who have sex with men.

[27] Henry E, Marcellin F, Yomb Y, Fugon L, Nemande S, Gueboguo C (2010). Factors associated with unprotected anal intercourse among men who have sex with men in Douala, Cameroon. Sex Transm Infect.

[28] Sanders EJ, Graham S, Okuku H, van der Elst E, Muhaari A, Davies A (2008). HIV-1 infection in high risk men who have sex with men in Mombasa, Kenya. AIDS.

[29] Brown B, Burrell E, Scheibe A, Wood R, Bekker LG Recruitment methods for enrolling high-risk men who have sex with men into an HIV prevention clinical trial.

[30] Batist E, Brown B, Baral S, Bekker LG Community engagement programming and HIV prevention: perspectives from men who have sex with men (MSM) in Cape Town's township communities.

[31] Moreau A, Tapsoba P, Ly A, Niang C, Diop A (2007). Implementing STI/HIV prevention and care interventions for men who have sex with men in Dakar, Senegal. Horizons research summary.

[32] Johnson CA, Cameron E (2007). Off the map: how HIV/AIDS programming is failing same-sex practicing people in Africa.

[33] Tucker A, de Swardt G, Struthers H, McIntyre J (2013). Understanding the needs of township men who have sex with men (MSM) health outreach workers: exploring the interplay between volunteer training, social capital and critical consciousness. AIDS Behavior.

[34] SANAC (2011). LGBT sector report.

[35] Grant RM, Lama JR, Anderson PL, McMahan V, Liu AY, Vargas L (2010). Preexposure chemoprophylaxis for HIV prevention in men who have sex with men. N Engl J Med.

[36] Tucker A (2009). Queer visibilities: space, identity and interaction in Cape Town.

[37] Eaton LA, Pitpitan EV, Kalichman SC, Sikkema KJ, Skinner D, Watt MH (2008). Men who report recent male and female sex partners in Cape Town, South Africa: an understudied and underserved population. South Africa: an understudied and underserved population.

[38] Campbell C, Gibbs A, Maimane S, Nair Y (2008). Hearing community voices: grassroots perceptions of an intervention to support health volunteers in South Africa. SAHARA J.

[39] Campbell C, Nair Y, Maimane S (2007). Building contexts that support effective community responses to HIV/AIDS: a South African case study. Am J Community Psychol.

[40] Jobson G (2010). HIV Prevention for MSM in Cape Town, South Africa: context, dynamics, and recommendations.

[41] Tucker A, Liht J, de Swardt G, Jobson G, Rebe K, McIntyre J (2013). An exploration into the role of depression and self-efficacy on township men who have sex with men's ability to engage in safer sexual practices. AIDS Care.

[42] Sandfort T, Yi H, Knox J, Reddy V (2013). Sexual partnership types as determinant of HIV risk in South African MSM: an event-level cluster analysis. AIDS Behav.

[43] Kaighobadi F, Konx J, Reddy V, Sandfort T (2013). Age and sexual risk among black men who have sex with men in South Africa: the mediating role of attitudes towards condoms. J Health Psychol.

# Sub article: A cross-sectional assessment of the burden of HIV and associated individual- and structural-level characteristics among men who have sex with men in Swaziland

**DOI:** 10.7448/IAS.16.4.18768

**Published:** 2013-12-02

**Authors:** Stefan D Baral, Sosthenes Ketende, Zandile Mnisi, Xolile Mabuza, Ashley Grosso, Bhekie Sithole, Sibusiso Maziya, Deanna L Kerrigan, Jessica L Green, Caitlin E Kennedy, Darrin Adams

**Affiliations:** 1 Key Populations Program, Department of Epidemiology, Center for Public Health and Human Rights, Johns Hopkins Bloomberg School of Public Health, Baltimore, MD, USA; 2 Swaziland National AIDS Program (SNAP), Ministry of Health and Social Welfare, Mbabane, Swaziland; 3 Rock of Hope, Manzini, Swaziland; 4 Department of Health Sciences, University of Stellenbosch, Stellenbosch, South Africa; 5 Department of Health, Behavior and Society, Johns Hopkins Bloomberg School of Public Health, Baltimore, MD, USA; 6 Population Services International, Swaziland; 7 Department of International Health, Johns Hopkins Bloomberg School of Public Health, Baltimore, MD, USA

**Keywords:** public health, men who have sex with men (MSM), Africa, HIV, Swaziland, epidemiology

## Abstract

**Introduction:**

Similar to other Southern African countries, Swaziland has been severely affected by HIV, with over a quarter of its reproductive-age adults estimated to be living with the virus, equating to an estimate of 170,000 people living with HIV. The last several years have witnessed an increase in the understanding of the potential vulnerabilities among men who have sex with men (MSM) in neighbouring countries with similarly widespread HIV epidemics. To date, there are no data characterizing the burden of HIV and the HIV prevention, treatment and care needs of MSM in Swaziland.

**Methods:**

In 2011, 324 men who reported sex with another man in the last 12 months were accrued using respondent-driven sampling (RDS). Participants completed HIV testing using Swazi national guidelines as well as structured survey instruments administered by trained staff, including modules on demographics, individual-level behavioural and biological risk factors, social and structural characteristics and uptake of HIV services. Population and individual weights were computed separately for each variable with a data-smoothing algorithm. The weights were used to estimate RDS-adjusted univariate estimates with 95% bootstrapped confidence intervals (BCIs). Crude and RDS-adjusted bivariate and multivariate analyses were completed with HIV as the dependent variable.

**Results:**

Overall, HIV prevalence was 17.6% (*n=*50/284), although it was strongly correlated with age in bivariate- [odds ratio (OR) 1.2, 95% BCI 1.15–1.21] and multivariate-adjusted analyses (adjusted OR 1.24, 95% BCI 1.14–1.35) for each additional year of age. Nearly, 70.8% (*n=*34/48) were unaware of their status of living with HIV. Condom use with all sexual partners and condom-compatible-lubricant use with men were reported by 1.3% (95% CI 0.0–9.7).

**Conclusions:**

Although the epidemic in Swaziland is driven by high-risk heterosexual transmission, the burden of HIV and the HIV prevention, treatment and care needs of MSM have been understudied. The data presented here suggest that these men have specific HIV acquisition and transmission risks that differ from those of other reproductive-age adults. The scale-up in HIV services over the past decade has likely had limited benefit for MSM, potentially resulting in a scenario where epidemics of HIV among MSM expand in the context of slowing epidemics in the general population, a reality observed in most of the world.

## Introduction

Swaziland is a small, land-locked, lower-middle-income country that is surrounded by South Africa and Mozambique; it has a population of approximately 1.1 million people and a life expectancy at birth of approximately 48 years [1]. Similar to other Southern African countries, Swaziland has been severely affected by HIV, with over a quarter of its reproductive-age adults (15–49) estimated to be living with the virus, equating to an estimate of 170,000 people living with HIV [2]. Moreover, the incidence of HIV appears to have peaked in 1998–1999 at 4.6% [95% confidence interval (CI) 4.27–4.95], according to estimates by the Joint United Nations Programme on HIV/AIDS (UNAIDS), while in 2009 it was estimated to be 2.7% (95% CI 2.2–3.1%) [3–6]. There appear to have been further declines in incidence according to 6054 person-years of follow-up data from 18,154 people followed from December 2010 to June 2011 as part of the Swaziland HIV Incidence Measurement Survey (SHIMS) longitudinal cohort. Overall incidence was approximately 2.4% (95% CI 2.1–2.7%), with incidence estimated to be 3.1% (95% CI 2.6–3.7) among women as compared to 1.7% (95% CI 1.3–2.1) among men [7]. Indeed, women and girls have been more burdened with HIV than men throughout the history of the HIV epidemic in Swaziland, with the HIV prevalence among women 15–24 in 2006 being estimated to be 22.6% compared to 5.9% among age-matched men and boys [5].

The 2009 Swaziland Modes of Transmission study characterized major drivers of incident HIV infections to be multiple concurrent partnerships before and during marriage as well as low levels of male circumcision [8]. These risk factors were confirmed in the SHIMS study, with risk factors for incident HIV infections among both men and women including not being married or living alone, having higher numbers of sex partners and having serodiscordant or unknown HIV status partners [7]. There are no known HIV prevalence estimates for key populations in Swaziland, including female sex workers (FSW) or men who have sex with men (MSM) [9,10]. The 2009 Swazi Modes of Transmission Study indicates that both sex work and male–male sexual practices are reportedly infrequent and assumed to be minor drivers of HIV risks in the setting of a broadly generalized HIV epidemic. However, the prevalence of these risk factors has not been measured in the HIV surveillance systems that are used to inform the Modes of Transmission Surveys [11]. The last several years have witnessed an increase in the understanding of the potential vulnerabilities among these same key populations through targeted studies including MSM in neighbouring countries with similarly widespread HIV epidemics [12,13].

The largest body of data is available from South Africa, where the first study completed in 1983 of 250 MSM demonstrated a high prevalence of HIV, syphilis and hepatitis B virus [14]. More recently, a study of rural South African men found that approximately 3.6% of men studied (*n=*46) reported a history of having sex with another man [15]. Among these men, HIV prevalence was 3.6 times higher than among men not reporting male partners (95% CI 1.0–13.0, *p=*0.05) [16]. There have also been several targeted studies of MSM in urban centres across South Africa that consistently highlight a population of men who have specific risk factors for HIV acquisition and transmission and limited engagement in the continuum of HIV care [17–19]. Relatively recent studies from other countries, including Lesotho, Malawi, Namibia and Botswana, have shown similar diverse populations of MSM [16,20,21]. Diversity among populations of MSM across Southern Africa manifests through diverse sexual orientations and practices ranging from those who are gay identified, with primarily male sexual partners, to those who are straight identified, with both male and female sexual partners [22]. Diversity has also been measured in the range of HIV-related risk practices among MSM, including understanding of the HIV acquisition and transmission risks associated with unprotected anal intercourse and of the levels of use of condoms and condom-compatible lubricants (CCLs) [23].

To better characterize vulnerabilities and HIV prevention, treatment and care needs among MSM in Swaziland, a cross-sectional assessment was completed to provide an unbiased estimate of the prevalence of HIV and syphilis among adult MSM in Swaziland. This study was completed in equal collaboration with the Swaziland National AIDS Program (SNAP) in the Ministry of Health. This study further sought to describe the significant correlates of prevalent infections, including individual behavioural characteristics, and describe social and structural HIV-related factors and risks for HIV infection among MSM.

## Methods

### Sampling

MSM in Swaziland were recruited via respondent-driven sampling (RDS), a peer referral sampling method designed for data collection among hard-to-reach populations [24]. Potential participants were required to be at least 18 years of age, report anal sex with another man in the previous 12 months, be able to provide informed consent in either English or siSwati, be willing to undergo HIV and syphilis testing and possess a valid recruitment coupon.

### Survey administration and HIV testing

All participants completed face-to-face surveys and received HIV and syphilis tests on site. Surveys were administered by trained members of the research staff and lasted approximately one hour. The study was completely anonymous and did not collect any identifiable information; we used verbal rather than signed consent to further ensure anonymity. Questions on socio-demographics (e.g., age, marital status and education), behavioural HIV-related risk factors (e.g., HIV-related knowledge, attitudes and risk behaviours) and structural factors (e.g., stigma, discrimination and social cohesion) were included [25]. HIV and syphilis tests were conducted by trained phlebotomists or nurses, according to official Swazi guidelines. Test results, counselling and any necessary treatment (for syphilis) and/or referrals (for HIV) were provided on site. Participant surveys and test results were linked using reproducible, yet anonymous, 10-digit codes.

### Analytical methods

Population and individual weights were computed separately for each variable by the data-smoothing algorithm using RDS for Stata [26]. The weights were used to estimate RDS-adjusted univariate estimates with 95% bootstrapped confidence intervals (BCIs). Crude bivariate regression analyses were also conducted to assess the association of HIV status with demographic variables as well as a selection of variables either expected or shown to be associated with HIV status in the literature. All demographic variables were then included in the initial multivariate logistic regression model regardless of the estimated strength of their crude bivariate association with HIV status. Non-demographic variables were included in the initial multivariate model if the chi-square *p* value of association with HIV status was ≤0.25 in the bivariate analyses. Most of the demographics variables, however, dropped out of the final model after controlling for other independent variables.

Because regression analyses of RDS data using sample weights are complicated due to the fact that weights are variable-specific [27], RDS-adjusted bivariate and multivariate analyses were conducted using individualized weights that were specific to the outcome variable (i.e., HIV status) [27]. The adjusted odds ratio (aOR) estimates were not statistically different from the unadjusted estimates in the bivariate analyses, although some slight differences were observed in the multivariate analyses. Thus, only the unadjusted odds ratios (ORs) are reported for bivariate analyses, while both are presented in Table 1 for multivariate analyses. All data processing and analyses were conducted using Stata 12.1 [28].

### Missing data

Eleven out of the 324 participants were excluded from this analysis due to missing data on key RDS-related variables. There were 29 out of 313 participants with missing data on at least one variable used in the multivariate analyses. Only two variables had data missing for more than three participants: age at first sex with another man (*n* missing=4) and knowledge about the type of anal sex position that puts you most at risk of HIV infection (*n* missing=6). Two of the 29 participants with missing data were living with HIV; thus, the effective crude HIV prevalence used in the multivariate model was 17.6% (50/284) versus 16.6% (52/313) without missing data, and RDS-adjusted 13.4% (95% BCI: 7.9–19.7; homophily: HIV−=−0.0991, HIV+=0.134) versus 12.7% (BCI: 7.3–18.1; homophily: HIV−=−0.0899, HIV+=0.1358) Although the total number of cases with missing data is not very small (9.3%: 29/313), the number missing by variable is very small. Due to the small change in HIV prevalence in the analysis sample compared to the complete sample as shown in this article, no effort was made to impute missing data. The 29 cases were excluded in the multivariate regression models.

### Sample size calculation

The sample size was calculated based on the ability to detect significant differences in condom use among MSM living with HIV and those not living with HIV. There were no known estimates of condom use among MSM in Swaziland, but previous studies of MSM from nearby countries estimated that consistent condom use during anal sex with other men among MSM is approximately 50% [19]. In addition, a systematic review and meta-analysis of the literature on behavioural interventions targeting MSM have demonstrated that behavioural interventions can increase reported condom use by approximately 16.5% in all risk categories of MSM [29,30]. Thus, this study was powered on the assumption that those who have received information about preventing HIV infection from other men would have a 16.5% increase in reported consistent condom use. A power analysis demonstrated that with 80% power, we would require 160 participants. Estimates of appropriate design effects for RDS have varied in the literature, and we used a design effect of 2, planning for the accrual of 324 MSM [31]. This sample size facilitates the detection of significant differences in HIV-related protective practices, such as consistent condom use, and targeted HIV-prevention measures, and is sufficient for key social factors such as experiences with stigma and discrimination.

### Ethics

The study received approval for research on human participants from both the National Ethics Committee of Swaziland as well as the Institutional Review Board of the Johns Hopkins Bloomberg School of Public Health.

## Results

Three hundred and twenty-four men were accrued from six seeds over a range of between 1 and 14 waves of accrual, with the largest recruitment chain including 123 participants. As shown in Table 2, the majority of men sampled were under 30 years of age, with a mean age of 23.1 and a mode of 22. The crude sample was relatively educated, although highly educated men were oversampled in this study when comparing the crude results to RDS-adjusted results (unadjusted 23.0% and adjusted 14.8%). Most of the study participants had never been married (98.2%, 95% CI 96.5–99.9), with only 13 men reporting either cohabitating with a woman or being married to a woman. Similarly, only about one in 10 men reported having children (10.5%, 95% CI 5.8–15.3). Notably, the majority of the sample of participants did not self-identify as straight or heterosexual, with approximately two-thirds reporting being gay and one-third reporting being bisexual. When asked about gender identification, nearly a quarter of the sample reported identifying as a woman, although the adjusted proportion was 15.7% (95% CI 10.4–20.9). More than one-tenth of men reported having been to jail or prison (13.2%, 95% CI 7.9–18.6). Among 71 men aged 18–19, the HIV prevalence was 0%, compared to 8.8% (*n=*6/68) among participants aged 20–21, 15% (*n=*9/60) among participants aged 22–23, 21.4% (*n=*12/56) among participants aged 24–26, and finally 43.1% (*n=*25/58) among participants aged 27–43 (data not shown). In total, 29.2% (*n=*14/48) of participants living with HIV reported previously being told that they had HIV, although four participants not found to be living with HIV reported being given this diagnosis.

**Table 1:** Sociodemographic characteristics of a sample of men who have sex with men in Swaziland in 2011

Variable	Categories	*N*	Crude percentage	RDS-adjusted percentage	95% confidence interval		Homophily (−1 to +1)
Age in years	Under 21	94	30.0	36.3	27.4	45.2	0.199
	21–25	142	45.4	45.1	36.3	53.8	0.143
	26–30	56	17.9	12.0	7.2	16.7	0.148
	31 and older	21	6.7	6.7	2.9	10.4	0.026
	Some secondary, high school or lower	108	34.5	44.8	35.6	53.9	0.104
Education level	Completed secondary or high school	133	42.5	40.4	32.4	48.4	0.119
	Post-high-school vocational training or higher	72	23.0	14.8	9.4	20.2	0.180
	Unemployed	97	32.3	30.7	22.5	38.9	0.189
Employment status	Employed	101	33.7	27.5	19.5	35.5	0.203
	Student	102	34.0	41.8	32.6	51.0	−0.001
Marital status with a woman	Married or cohabitating	13	4.2	1.8	0.1	3.5	−0.018
	Single, never married	298	95.8	98.2	96.5	99.9	−1.423
Current housing tenure	Renting place	92	29.4	27.7	20.6	34.9	0.046
	Own place	51	16.3	18.3	12.0	24.6	−0.126
	Staying with someone	101	32.3	34.8	27.1	42.5	0.119
	Family	42	13.4	10.6	5.6	15.5	0.201
	Other	27	8.6	8.6	4.5	12.8	−0.095
Urban or rural origin	Urban	192	61.5	61.0	52.6	69.5	0.101
	Rural	120	38.5	39.0	30.5	47.4	0.172
Number of children	Zero	274	87.8	89.5	84.7	94.2	−0.174
	One or more	38	12.2	10.5	5.8	15.3	0.115
Gender Identification	Man	225	72.6	82.5	76.9	88.1	−0.29
	Woman	79	25.5	15.7	10.4	20.9	0.17
	Both	6	1.9	1.8	0.0	3.9	−0.018
Sexual orientation identification	Gay or homosexual	198	63.5	56.3	48.0	64.6	0.242
	Bisexual	109	34.9	40.5	32.3	48.6	0.062
	Heterosexual or straight	5	1.6	3.2	0.0	7.4	0.096
Age at first sex with a man	Under 21 years	238	77.0	77.6	70.7	84.6	0.110
	21 and above	71	23.0	22.4	15.4	29.3	0.083
Ever been to jail or prison?	No	276	88.2	86.8	81.4	92.1	0.216
	Yes	37	11.8	13.2	7.9	18.6	0.157

**Table 2:** HIV-related sexual and drug risk factors among MSM in Swaziland

Variable	Categories	*N*	Crude percentage	RDS-adjusted percentage	95% CI	
Number of male sexual partners in the past 12 months	1	103	33.0	42.1	34.0	50.2
	2	68	21.8	20.3	14.7	26.0
	3	70	22.4	20.3	14.2	26.5
	4+	71	22.8	17.2	11.7	22.7
Number of main male partners in the past 12 months	1	37	11.8	17.1	10.7	23.6
	2	183	58.5	57.2	49.7	64.7
	3	61	19.5	18.5	13.0	24.1
	4+	32	10.2	7.1	3.9	10.4
Number of male casual sexual partners in the past 12 months	None	132	42.4	46.6	39.0	54.2
	1–2	127	40.8	41.1	33.8	48.5
	3+	52	16.7	12.3	7.7	16.8
Number of female sexual partners in the past 12 months	None	198	64.3	53.6	44.9	62.4
	1	52	16.9	19.5	12.5	26.5
	2	29	9.4	15.1	8.7	21.6
	3+	29	9.4	11.8	6.2	17.4
Number of both male and female sex partners in the past 12 months	Only male	221	70.6	64.3	56.4	72.3
	Male and female	92	29.4	35.7	27.7	43.6
In general, how often have you used a condom in the past six months?	Never or almost never	30	9.7	11.5	6.0	17.0
	Sometimes	79	25.6	27.0	19.7	34.4
	Almost always	58	18.8	18.9	12.9	24.9
	Always	141	45.8	42.5	34.7	50.3
Had unprotected insertive anal sex in the past 12 months	No	190	60.9	55.5	46.8	64.2
	Yes	122	39.1	44.5	35.8	53.2
Had unprotected receptive anal sex in the past 12 months	No	211	68.7	69.8	62.7	76.8
	Yes	96	31.3	30.2	23.2	37.3
Condom use with main male partners in the past 12 months	Not always	137	47.2	51.9	41.8	62.0
	Always	153	52.8	48.1	38.0	58.2
Condom use with casual male partners in the past 12 months	Not always	54	17.3	16.6	10.2	23.0
	Always	150	47.9	46.1	38.6	53.6
	No casual partner	109	34.8	37.3	29.8	44.9
Condom use with regular female partners in the past 12 months	Not always	47	49.5	61.8	41.3	82.3
	Always	48	50.5	38.2	17.7	58.7
Condom use with casual female partners in the past 12 months	Not always	31	45.6	55.6	25.2	86.0
	Always	37	54.4	44.4	14.0	74.8
Used water-based lubricant (WBL) in the past 12 months	No	203	64.9	76.3	69.8	82.8
	Uses WBL	110	35.1	23.7	17.2	30.2
Safe sex with men (condoms and water-based lubricant) in the past 12 months	Does not	257	82.1	87.4	82.4	92.4
	Does	56	17.9	12.6	7.6	17.6
Safe sex with women (condoms) in the past 12 months	Does not	66	60.0	75.3	60.5	90.0
	Does	44	40.0	24.7	10.0	39.5
Safe sex with both men and women in the past 12 months	Does not	104	95.7	98.7	90.3	100.0
	Does	6	4.3	1.3	0.0	9.7
Injected illicit drugs in the past 12 months	No	304	97.1	97.7	96.1	99.3
	Yes	9	2.9	2.3	0.7	3.9
Used non-injection illicit drugs in the past 12 months	No	203	65.1	66.4	58.5	74.3
	Yes	109	34.9	33.6	25.7	41.5
Used alcohol in the last month	None	121	39.0	36.1	28.7	43.5
	At least one day	189	61.0	63.9	56.5	71.3
Which is the safest lubricant to use during anal sex?	Non-WBL	128	49.8	63.5	53.6	73.4
	WBL	129	50.2	36.5	26.6	46.4
Can you get HIV from sharing a needle to inject illegal drugs?	No	3	1.0	1.0	−0.2	2.2
	Yes	303	99.0	99.0	97.8	100.2
What type of sex puts you most at risk for HIV infection?	Vaginal	110	35.1	43.7	36.1	51.2
	Anal	75	24.0	18.2	12.4	24.0
	Oral	25	8.0	8.6	3.8	13.5
Which type of anal sex position puts you most at risk for HIV infection?	Insertive (top)	63	20.5	24.0	17.1	30.9
	Receptive (bottom)	95	30.9	31.1	23.8	38.4
	Insertive and receptive anal sex carry equal risk	149	48.5	44.9	36.6	53.2
Answered all of the above correctly	No	278	88.8	90.9	87.0	94.8
	Yes	35	11.2	9.1	5.2	13.0

The majority of men had multiple male sexual partners over the past 12 months (57.9%, 95% CI 49.8–66.0). Moreover, most study participants had multiple main sexual partners, or boyfriends, over the past 12 months (82.9%, 95% CI 76.4–89.3) (Table 3). About one-third of participants reported having had both male and female sexual partners in the previous 12 months (35.7%, 95% CI 27.7–43.6). Approximately one-half of the participants reported always using condoms during sex, although significant numbers of men reported both unprotected insertive and receptive anal intercourse in the past 12 months. Condom use was not significantly different between main and casual male or female partners. Overall, safe sex with other men, defined as always using condoms and water-based lubricants over the last 12 months, was not common, with 12.6% (95% CI 7.6–12.6) measured to report this behaviour. Safe sex, defined as condom use with all sexual partners over the last 12 months, was significantly higher with female partners (at 40.0% in the crude assessment) than with male partners (*p<*0.05). Overall, safe sex with all sexual partners was uncommon and was reported by 4.3% (RDS-adjusted 1.3%, 95% CI 0.0–9.7). Knowledge of basic questions related to safe sex for MSM, including sexual positioning, type of sexual act and lubricant use, was low, with 11.2% (RDS-adjusted 9.1%, 95% CI 5.2–13.0) of participants providing correct answers.

**Table 3:** Service uptake and structural HIV risks among MSM in Swaziland

Variable	Categories	*N*	Crude percentage	RDS-adjusted percentage	95% CI	
Participated in any meetings related to HIV/AIDS in the past 12 months	No	175	55.9	58.5	51.1	65.8
	Yes	138	44.1	41.5	34.2	48.9
Participated in any meetings related to HIV/AIDS in the past 12 months related to MSM	No	243	78.4	83.5	78.1	88.8
	Yes	67	21.6	16.5	11.2	21.9
Received information about preventing HIV from sex with women in last 12 months	No	60	19.4	20.9	14.5	27.2
	Yes	250	80.6	79.1	72.8	85.5
Received information about preventing HIV from sex with other men in last 12 months	No	226	72.4	78.5	72.9	84.1
	Yes	86	27.6	21.5	15.9	27.1
Level of concern related to HIV in the last 12 months	Not worried	86	27.6	31.8	24.9	38.8
	Not very worried	61	19.6	18.2	12.1	24.2
	Somewhat worried	52	16.7	16.8	10.0	23.6
	Very worried	113	36.2	33.2	26.0	40.3
Access to condoms: do you have them when you need them?	No access	3	1.0	1.0	-0.4	2.3
	Difficult or little access	58	18.6	16.8	11.2	22.4
	Some access	36	11.6	12.6	7.2	18.0
	Very easy access	214	68.8	69.6	61.9	77.4
Symptoms of sexually transmitted infection (STI) in the past 12 months	No	247	79.2	78.5	72.4	84.6
	Yes	65	20.8	21.5	15.4	27.6
Tested for STI in the past 12 months	No	266	87.2	86.1	80.5	91.7
	Yes	39	12.8	13.9	8.3	19.5
Diagnosis of STI in the past 12 months	No	287	92.6	92.2	88.3	96.1
	Yes	23	7.4	7.8	3.9	11.7
Been tested for HIV in the past 12 months	No	144	46.0	49.3	41.8	56.8
	Yes, once	94	30.0	31.2	24.2	38.2
	Yes, >1	75	24.0	19.5	13.5	25.4
Ever been told that you have HIV?	No	284	94.0	95.7	92.5	98.9
	Yes	18	6.0	4.3	1.1	7.5
Perceived human rights violations	No	63	20.1	20.4	14.5	26.3
	Yes	250	79.9	79.6	73.7	85.5
Experienced human rights violations	No	152	48.6	48.9	40.5	57.2
	Yes	161	51.4	51.1	42.8	59.5
Disclosure to healthcare workers	No	218	69.6	75.0	69.0	81.0
	Yes	95	30.4	25.0	19.0	31.0
Disclosure to family	No	146	46.6	56.0	48.3	63.6
	Yes	167	53.4	44.0	36.4	51.7

Table 4 demonstrates levels of service uptake, with evidence of statistically significantly lower levels of access to targeted services focused on preventing HIV transmission via sex between men as compared to sex between men and women (*p<*0.05 for both). Notably, only about half of the sample was somewhat or very worried about HIV. Just under half of the men who had symptoms of a sexually transmitted infection (STI) were tested in the previous 12 months, with 7.8% (95% CI 3.9–11.7) diagnosed in this same time frame. About half of the sample had been tested for HIV in the previous 12 months (50.7%, 95% CI 43.2–59.2), including some who were tested more than one time. Reports of any experienced rights violations related to sexual practices, including denial of care, police-mediated violence and physical or verbal harassment, were reported by about half of the sample, although perceived rights violations related to sexual orientation (fear of seeking healthcare and fear of walking in the community) were more common, with 79.6% (95% CI 73.7–85.5) calculated to report this. Disclosure of sexual practices to healthcare workers was reported by one-quarter of the sample (25.0%, 95% CI 19.0–31.0), whereas about half of the participants (44.0%, 95% CI 36.4–51.7) had reported disclosure of sexual practices to a family member.

**Table 4:** Bivariate and multivariate associations with HIV status among men who have sex with men (MSM) in Swaziland

		Bivariate		Multivariate – crude		Multivariate – RDS weighted	
				
Variable	Categories	Estimate	[95% CI]	Estimate	[95% CI]	Weighted estimate	Weighted estimate 95% CI
Current age	Years	1.23	[1.15–1.31]	1.24***	[1.14–1.35]	1.28***	[1.15–1.43]
	Man	1		1		1	
Gender	Woman	2.14	[0.90–5.05]	3.96**	[1.66–9.43]	3.23*	[1.07–9.71]
	Both	–		–		–	
Education level	Some secondary, high school or lower	1		1		1	
	Completed secondary or high school	1.06	[0.44–2.56]	1.32	[0.54–3.18]	1.51	[0.46–5.00]
	Post-high-school vocational training or higher	1.34	[0.47–3.77]	0.56	[0.20–1.57]	0.62	[0.18–2.16]
Age at first sex with another man	Under 21 years	1		1		1	
	21 and above	2.38	[0.99–5.72]	1.24	[0.49–3.14]	0.71	[0.18–2.75]
Urban or rural origin	Urban	1		1		1	
	Rural	1.99	[0.91–4.35]	0.79	[0.34–1.79]	1.33	[0.45–3.93]
Ever been to jail or prison?	No	1		1		1	
	Yes	2.75*	[1.08–7.00]	3.00*	[1.01–8.85]	4.37*	[1.38–13.84]
Diagnosis with an STI other than HIV in last 12 months	No	1		1		1	
	Yes	1.57	[0.49–5.07]	6.26**	[1.68–23.39]	4.30*	[1.04–17.72]
Number of casual male partners in the last 12 months	None	1		1		1	
	1–2	0.51	[0.20–1.26]	0.33*	[0.13–0.85]	0.26*	[0.08–0.85]
	3+	1.12	[0.42–2.98]	1.04	[0.37–2.95]	0.50	[0.13–1.97]
Which type of anal sex position puts you most at risk for HIV infection?	Insertive (top)	1		1		1	
	Receptive (bottom)	0.91	[0.33–2.54]	0.49	[0.17–1.42]	0.53	[0.14–2.08]
	Insertive and receptive anal sex carry equal risk	0.96	[0.37–2.54]	0.39	[0.14–1.06]	1.43	[0.32–6.41]
In the past 12 months, have you used any non-injectable drug that was not prescribed?	No	1		1		1	
	Yes	0.84	[0.375–1.865]	0.356*	[0.136–0.935]	0.366	[0.12–1.11]
What kind of access to condoms do you have when you need them?	No access	1		1		1	
	Difficult or little access	0.13	[0.007–2.380]	0.008**	[0.000–0.224]	0.031	[0.001–1.23]
	Some access	0.36	[0.021–6.115]	0.043	[0.002–1.022]	0.170	[0.01–5.35]
	Very easy access	0.49	[0.034–7.054]	0.043*	[0.002–0.893]	0.264	[0.007–10.020]
In the past 30 days, how many days did you drink at least one drink of alcohol?	Zero	1		1		1	
	At least one day	1.30	[0.55–3.07]	1.81	[0.74–4.41]	2.19	[0.60–7.96]
Analysis sample						284	284

Exponentiated coefficients; 95% CI=95% confidence intervals

* *p*<0.05

** *p*<0.01

*** *p*<0.001.

HIV prevalence was strongly correlated with age in both bivariate analyses (OR 1.23, 95% BCI 1.15–1.21) for each year of age and multivariate-adjusted analyses (aOR 1.24, 95% BCI 1.14–1.35) (Table 1). Other statistically significant associations with HIV in adjusted analyses included identifying as the female gender, having ever been to jail or prison, having lower numbers of casual partners, being diagnosed with an STI in the last 12 months and having easier access to condoms.

## Discussion

In the country with the highest HIV prevalence in the world, this study describes the burden of HIV and associated characteristics among MSM who were accrued using RDS. Interpreting the prevalence of HIV among MSM and its relationship with the widespread and generalized female-predominant epidemic in Swaziland is challenging on a number of levels. The significant association between HIV and age suggests that the expanding epidemic among MSM in Swaziland is not new and represents cumulative HIV acquisition risk exposures. The burden of HIV among all men aged 15–19 is approximately 2% in Swaziland, increasing to 12.4% among those aged 20–24 and up to 44.9% among those aged 35–39. While the participants in our study were relatively young, the HIV prevalence was consistent with that of general reproductive-age men until age 24–26, when the prevalence of HIV among age-matched MSM appears to be higher than that of other men sampled as part of the Swazi DHS study (Figure 1) [2]. Given that relatively few men in our sample reported female sexual partners, their HIV acquisition and transmission risks are likely different from those of other men in Swaziland and potentially more related to anal intercourse. Conversely, Swaziland may be among a small number of countries where even the low acquisition risks associated with insertive penile-vaginal intercourse is counterbalanced by the significantly higher HIV prevalence among women, resulting in significant acquisition risks associated with sex with women. However, the idea that acquisition risk for MSM primarily related to sex with other men is reinforced by the results that condom use was lower with male sexual partners than with female sexual partners. Condoms being used more frequently during sex with women as compared to sex with other men have been observed in other studies of MSM across Sub-Saharan Africa and provide an argument against MSM being a population that bridges the HIV epidemic from within their sexual networks to lower risk heterosexual networks [19,20,32,33]. However, to answer this question, phylogenetic studies and the characterization of sexual networks are needed to better describe patterns of HIV transmission.

**Figure 1:** Prevalence of HIV by age among Swazi men who have sex with men, 2011.

Participants were far more likely to have received information about preventing HIV infection during sex with women as compared to sex with other men. This lack of access to or uptake of information, education and communication services has resulted in participants in this study having a limited knowledge base of the sexual risks associated with same-sex practices. Primarily, participants incorrectly believed that unprotected penile-vaginal intercourse was associated with the highest risk of HIV transmission, consistent with earlier studies of MSM across Sub-Saharan Africa. Numerous studies have shown the opposite: HIV is far more efficiently transmitted during anal intercourse as compared to vaginal intercourse [13,34]. There was also limited knowledge related to the importance of water-based lubricants being CCLs, which is especially important during anal intercourse given the absence of physiological lubrication in the anal canal. The importance of CCL was underscored as ultimately being the determining factor in just six study participants reporting safe sex with all partners in this study. Thus, while there is significant provision of general HIV-prevention messaging across Swaziland, there has been limited information focused on educating MSM on how to prevent HIV acquisition and transmission during sex with other men. Data suggest that starting with simple and proven approaches, including peer education programmes, is necessary to educate these men about their risks and protective behavioural strategies [35]. However, these approaches will likely not be sufficient to change the trajectory of HIV epidemics given the high risk of infection associated with unprotected anal intercourse with non-virally suppressed HIV serodiscordant partners. Thus, moving forward necessitates assessing the feasibility of combination approaches that integrate advances such as antiretroviral-mediated pre-exposure prophylaxis and universal access to antiretroviral therapy for people living with HIV [13]. However, the success or failure in achieving coverage with these HIV prevention, treatment and care approaches among MSM will, in part, be determined by the level of stigma affecting MSM.

It is now broadly accepted that addressing the needs of people living with HIV is vital to protect their own health as well as prevent onward transmission of HIV [36]. In addition, mean and total viral loads in a population have been linked to population-level transmission rates of HIV [37]. Only a quarter of the men living with HIV in this study were aware of their diagnosis, demonstrating the need to increase HIV testing, linkage to CD4 testing, and antiretroviral treatment and adherence support for those who are eligible. A recent systematic review and meta-analysis of self-testing for HIV in both low- and high-risk populations demonstrated that self-testing was both appropriate and associated with increased uptake of HIV tests [38]. This may be especially relevant in the Swazi context, where fear of seeking healthcare was prevalent, suggesting the need to study new strategies to overcome barriers to HIV testing among MSM in Swaziland, including leveraging community networks and potentially self-testing [39]. In this study, being a person living with HIV was associated with lower numbers of casual male partners in the last 12 months. This relationship appeared to be stronger among those who were aware of their status, although it was not statistically significant because of limited numbers. In addition, these data are consistent with earlier research findings that simply being made aware of one's status of living with HIV can change one's sexual practices to decrease onward transmission [40]. This further argues for implementation science research focused on optimal strategies to scale-up HIV testing for MSM in Swaziland [41].

Over one-quarter of participants in this study self-identified as women, and this was independently associated with living with HIV. There is nearly a complete dearth of information related to HIV among transgender people across Sub-Saharan Africa [42,43]. However, where transgender people have been studied, they have been found to be the most vulnerable to HIV acquisition because of increased structural barriers to HIV prevention, treatment and care services and because of increased sexual risks, including unprotected receptive anal intercourse [43]. Given the limited information available about transgender people, transgender was assessed in this study as both a sexual orientation and a gender identity. There was a significant disconnect between these two as no participants self-identified as being transgender. Ultimately, further ethnographic research is needed to better understand the HIV-prevention needs of transgender people in Swaziland.

Having been to jail was also independently associated with living with HIV among MSM in this study. Globally, incarceration has been shown to be an important risk factor for HIV, given the limited access to HIV-prevention services such as condoms and CCLs, the interruption of HIV treatment as well as exposure to higher risk sexual partners [44–47]. While further research is needed on same-sex practices within jails, there is likely a need to provide HIV-prevention services for men in Swazi prison settings [47].

The methods employed in this study have several limitations. While RDS is an effective approach to characterize asymptotically unbiased estimates intended to approximate population-based estimates of characteristics in the absence of a meaningful sampling frame, there are still several uncertainties in the most appropriate tools for interpretation of these data [48]. Moreover, the sample of men accrued here was relatively young, consistent with recruitment challenges observed in other studies of MSM across sub-Saharan Africa. While we conducted significant engagement with older MSM, fear associated with inadvertent disclosure limited their participation in the study. Only with improved social environments will more information about the needs of older MSM become available in difficult contexts [49]. In addition, while RDS was used to accrue a diverse sample, all of the seeds were connected with Rock of Hope, a newly registered organization serving the needs of lesbian, gay, bisexual and transgender populations in Swaziland. We thus may have overestimated actual service uptake among MSM in Swaziland.

## Conclusions

The implementation of the research project was guided by recent guidelines to inform HIV-related research with MSM in rights-constrained environments [50]. While these men had not been previously engaged in research on HIV prevention, treatment and care, the success of this study highlights the fact that accrual of this population is both feasible and informative for the HIV response in Swaziland. Moreover, the interconnected social and sexual networks leveraged for accrual can likely serve to disseminate HIV-prevention approaches via MSM throughout the country. While the epidemic in Swaziland is one driven by heterosexual transmission, the burden of HIV and the HIV prevention, treatment and care needs of MSM have been understudied, and these men have been underserved in the context of large-scale programmes [51]. The data presented here suggest that these men have specific HIV acquisition and transmission risks that differ from those of other reproductive-age adults. Encouragingly, Swaziland has seen declines in the rate of new HIV infections over the last seven years, and these declines are related to HIV testing and treatment scale-up [5]. However, the increase in HIV services likely has had limited benefit for MSM, which may result in a scenario where epidemics of MSM expand in the context of slowing epidemics in the general population – a reality observed in most of the world [13].

## References

[1] World B (2009). Gross national income per capita 2008. Atlas method and PPP.

[2] Macro I, Swaziland Central Statistics Office (2008). Swaziland: demographic and health survey 2006–2007.

[3] UNAIDS (2009). AIDS epidemic update 2009.

[4] UNAIDS (2012). Report on the global AIDS epidemic.

[5] UNAIDS, NERCHA, Ministry of Health S, UNICED (2010). Monitoring the declaration of the commitment on HIV and AIDS (UNGASS): Swaziland country report.

[6] Ghys PD, Saidel T, Vu HT, Savtchenko I, Erasilova I, Mashologu YS (2003). Growing in silence: selected regions and countries with expanding HIV/AIDS epidemics. AIDS.

[7] Reed JB, Justman J, Bicego G, Donnell D, Bock N, Ginindza H (2012). Estimating national HIV incidence from directly observed seroconversions in the Swaziland HIV Incidence Measurement Survey (SHIMS) longitudinal cohort (FRLBX02).

[8] Swaziland National Emergency Response Council on HIV, AID (2009). Swaziland: HIV prevention response and modes of transmission analysis.

[9] UNFPA, UNAIDS, Swaziland M, Swaziland National Emergency Response Council on HIV, AIDS (2007).

[10] Baral S, Sifakis F, Cleghorn F, Beyrer C (2007). Elevated risk for HIV infection among men who have sex with men in low- and middle-income countries 2000–2006: a systematic review. PLoS Med.

[11] Baral S, Phaswana-Mafuya N (2012). Rewriting the narrative of the epidemiology of HIV in sub-Saharan Africa. Sahara J.

[12] Baral S, Beyrer C, Muessig K, Poteat T, Wirtz AL, Decker MR (2012). Burden of HIV among female sex workers in low-income and middle-income countries: a systematic review and meta-analysis. Lancet Infect Dis.

[13] Beyrer C, Baral SD, van Griensven F, Goodreau SM, Chariyalertsak S, Wirtz AL (2012). Global epidemiology of HIV infection in men who have sex with men. Lancet.

[14] Sher R (1989). HIV infection in South Africa, 1982–1988 – a review. S Afr Med J.

[15] Jewkes R, Dunkle K, Nduna M, Levin J, Jama N, Khuzwayo N (2006). Factors associated with HIV sero-positivity in young, rural South African men. Int J Epidemiol.

[16] Baral S, Adams D, Lebona J, Kaibe B, Letsie P, Tshehlo R (2011). A cross-sectional assessment of population demographics, HIV risks and human rights contexts among men who have sex with men in Lesotho. J Int AIDS Soc.

[17] Baral S, Burrell E, Scheibe A, Brown B, Beyrer C, Bekker LG (2011). HIV risk and associations of HIV infection among men who have sex with men in peri-urban Cape Town, South Africa. BMC Public Health.

[18] Lane T, Raymond HF, Dladla S, Rasethe J, Struthers H, McFarland W (2011). High HIV prevalence among men who have sex with men in Soweto, South Africa: results from the Soweto Men's study. AIDS Behav.

[19] Rispel LC, Metcalf CA (2009). Breaking the silence: South African HIV policies and the needs of men who have sex with men. Reprod Health Matters.

[20] Beyrer C, Trapence G, Motimedi F, Umar E, Iipinge S, Dausab F (2010). Bisexual concurrency, bisexual partnerships, and HIV among Southern African men who have sex with men. Sex Transm Infect.

[21] Baral S, Trapence G, Motimedi F, Umar E, Iipinge S, Dausab F (2009). HIV prevalence, risks for HIV infection, and human rights among men who have sex with men (MSM) in Malawi, Namibia, and Botswana. PLoS One.

[22] Reddy V, Sandfort T, Rispel L (2009). From social silence to social science: same-sex sexuality, HIV & AIDS and gender in South Africa.

[23] Baral S, Scheibe A, Sullivan P, Trapence G, Lambert A, Bekker LG (2013). Assessing priorities for combination HIV prevention research for men who have sex with men (MSM) in Africa. AIDS Behav.

[24] Heckathorn DD (1997). Respondent-driven sampling: a new approach to the study of hidden populations. Soc Probl.

[25] Baral S, Logie CH, Grosso A, Wirtz AL, Beyrer C (2013). Modified social ecological model: a tool to guide the assessment of the risks and risk contexts of HIV epidemics. BMC Public Health.

[26] Schonlau M, Liebau E (2012). Respondent-driven sampling. Stata J.

[27] Johnston L, O'Bra H, Chopra M, Mathews C, Townsend L, Sabin K (2010). The associations of voluntary counseling and testing acceptance and the perceived likelihood of being HIV-infected among men with multiple sex partners in a South African township. AIDS Behav.

[28] StataCorp (2010). Stata statistical software: release 11.1. College Station.

[29] Beyrer C, Wirtz A, Walker D, Johns B, Sifakis F, Baral S, Bank W (2011). The global HIV epidemics among men who have sex with men (MSM): epidemiology, prevention, access to care, costs, and human rights.

[30] Johnson WD, Diaz RM, Flanders WD, Goodman M, Hill AN, Holtgrave D (2008). Behavioral interventions to reduce risk for sexual transmission of HIV among men who have sex with men. Cochrane Database Syst Rev.

[31] Salganik MJ (2006). Variance estimation, design effects, and sample size calculations for respondent-driven sampling. J Urban Health.

[32] Onyango-Ouma W, Birungi H, Geibel S (2005). Understanding the HIV/STI risks and prevention needs of men who have sex with men in Nairobi, Kenya.

[33] Onyango-Ouma W, Birungi H, Geibel S (2009). Engaging men who have sex with men in operations research in Kenya. Cult Health Sex.

[34] Baggaley RF, White RG, Boily MC (2010). HIV transmission risk through anal intercourse: systematic review, meta-analysis and implications for HIV prevention. Int J Epidemiol.

[35] Sullivan PS, Carballo-Dieguez A, Coates T, Goodreau SM, McGowan I, Sanders EJ (2012). Successes and challenges of HIV prevention in men who have sex with men. Lancet.

[36] Gardner EM, McLees MP, Steiner JF, del Rio C, Burman WJ (2011). The spectrum of engagement in HIV care and its relevance to test-and-treat strategies for prevention of HIV infection. Clin Infect Dis.

[37] Das M, Chu PL, Santos GM, Scheer S, Vittinghoff E, McFarland W (2010). Decreases in community viral load are accompanied by reductions in new HIV infections in San Francisco. PLoS One.

[38] Pant Pai N, Sharma J, Shivkumar S, Pillay S, Vadnais C, Joseph L (2013). Supervised and unsupervised self-testing for HIV in high- and low-risk populations: a systematic review. PLoS Med.

[39] Trapence G, Collins C, Avrett S, Carr R, Sanchez H, Ayala G (2012). From personal survival to public health: community leadership by men who have sex with men in the response to HIV. Lancet.

[40] Marks G, Crepaz N, Senterfitt JW, Janssen RS (2005). Meta-analysis of high-risk sexual behavior in persons aware and unaware they are infected with HIV in the United States: implications for HIV prevention programs. J Acquir Immune Defic Syndr.

[41] Padian NS, Holmes CB, McCoy SI, Lyerla R, Bouey PD, Goosby EP (2011). Implementation science for the US President's Emergency Plan for AIDS Relief (PEPFAR). J Acquir Immune Defic Syndr.

[42] Jobson G (2012). Transgender in Africa: invisible, inaccessible, or ignored?. SAHARA J.

[43] Baral SD, Poteat T, Stromdahl S, Wirtz AL, Guadamuz TE, Beyrer C (2012). Worldwide burden of HIV in transgender women: a systematic review and meta-analysis. Lancet Infect Dis.

[44] Wolfe MI, Xu F, Patel P, O'Cain M, Schillinger JA, St Louis ME (2001). An outbreak of syphilis in Alabama prisons: correctional health policy and communicable disease control. Am J Public Health.

[45] Beyrer C, Jittiwutikarn J, Teokul W, Razak MH, Suriyanon V, Srirak N (2003). Drug use, increasing incarceration rates, and prison-associated HIV risks in Thailand. AIDS Behav.

[46] Harris RM, Sharps PW, Allen K, Anderson EH, Soeken K, Rohatas A (2003). The interrelationship between violence, HIV/AIDS, and drug use in incarcerated women. J Assoc Nurses AIDS Care.

[47] Kyomya M, Todyrs KW, Amon JJ (2012). Laws against sodomy and the HIV epidemic in African prisons. Lancet.

[48] Salganik MJ (2012). Commentary: respondent-driven sampling in the real world. Epidemiology.

[49] Semugoma P, Nemande S, Baral SD (2012). The irony of homophobia in Africa. Lancet.

[50] AMFAR, Center for Public Health and Human Rights JHSoPH (2011). Respect, protect, fulfill: best practices guidance in conducting HIV research with gay, bisexual, and other men who have sex with men (MSM) in rights-constrained environments.

[51] AMFAR, Center for Public Health and Human Rights, JHSPH (2013). Achieving an AIDS-free generation for gay men and other MSM in Southern Africa.

# Sub article: Building the evidence base for urgent action: HIV epidemiology and innovative programming for men who have sex with men in sub-Saharan Africa

**DOI:** 10.7448/IAS.16.4.18903

**Published:** 2013-12-02

**Authors:** R Cameron Wolf, Alison Surdo Cheng, Laurent Kapesa, Delivette Castor

**Affiliations:** 1 United States Agency for International Development, Office of HIV/AIDS, Washington, DC, USA; 2 United States Agency for International Development, West Africa, Accra, Ghana

**Keywords:** men who have sex with men, Sub-Saharan Africa, epidemiology, HIV programmes, stigma and discrimination

## Abstract

While still an understudied area, there is a growing body of studies highlighting epidemiologic data on men who have sex with men (MSM) in sub-Saharan Africa (SSA) which challenge the attitudes of complacency and irrelevancy among donors and country governments that are uncomfortable in addressing key populations (KPs). While some of the past inaction may be explained by ignorance, new data document highly elevated and sustained HIV prevalence that is seemingly isolated from recent overall declines in prevalence. The articles in this series highlight new studies which focus on the stark epidemiologic burden in countries from concentrated, mixed and generalized epidemic settings. The issue includes research from West, Central, East and Southern Africa and explores the pervasive impact of stigma and discrimination as critical barriers to confronting the HIV epidemic among MSM and the intersecting stigma and marginalization found between living with HIV and sexual minority status. Interventions to remove barriers to service access, including those aimed at training providers and mobilizing communities even within stigmatized peri-urban settings, are featured in this issue, which further demonstrates the immediate need for comprehensive action to address HIV among MSM in all countries in the region, regardless of epidemic classification.

The HIV epidemic in sub-Saharan Africa (SSA) is dynamic with regional and temporal variation. The 2012 Report on the Global AIDS Epidemic reports a decline in HIV incidence among the general population (GP) by 25%, a decrease by 40% in HIV-related mortality, and that more than half of people living with HIV (PLHIV) who are eligible for treatment were on treatment in SSA [1]. The UN classifies countries as *low level* when HIV prevalence in the GP, as measured by HIV surveillance data, is under 1% and key population (KP) prevalence does not exceed 5%, where KPs are defined as female sex workers (FSWs), men who have sex with men (MSM) and people who inject drugs (PWID); *concentrated* epidemics are where HIV prevalence is under 1% in the GP, but any KP (e.g., FSW) prevalence consistently exceeds 5%; and *generalized* epidemics are where HIV prevalence exceeds 1% in the GP regardless of HIV status among KPs [1,2].

An epidemic appraisal proposed by Wilson and Halperin and others that characterizes the typology of HIV transmission within the country, rather than simply crude estimates of incidence and prevalence at the national level, called for reclassification of countries based on transmission dynamics [3,4]. They posited that *concentrated* epidemics are driven by KPs, and they added *mixed* epidemic settings, where both the GP and KPs play a role in HIV transmission, and *generalized* epidemics, in which they argued that contribution to new infections from KPs is insignificant. But this is debatable.

UNAIDS estimates HIV prevalence to be 17.9% among MSM in SSA. Yet there are limited epidemiological data for MSM or KPs in general in this region. For programmes to be well aligned, we need to better understand the epidemiologic and behavioural burden and social drivers of HIV within KP groups in all epidemic settings. The articles in this series focus on building the literature on epidemiology, social drivers of transmission and programmatic innovations for MSM in four regions of SSA: West, Central, East and Southern Africa. Respondent-driven sampling (RDS) methods were used to engage networks of MSM in these pioneering studies, which characterize the HIV epidemiology among MSM in Cameroon, Senegal, Malawi and Swaziland as elevated and sustained when compared to men in the GP. This supplement also includes a meta-analysis of prevalence studies from KPs in Central and West Africa, which helps to ground our understanding of MSM within the broader context of KPs.

The extraordinary burden of internalized and external stigma and discrimination creates the paralyzing barriers to MSM access for prevention, care and treatment services that are notable across these studies. Key dynamics of intersecting stigmas of HIV and sexual orientation among HIV-positive MSM are also explored in Swaziland [5]. Structural interventions to address the pervasive stigma in healthcare settings have been called for by the public health community, and this supplement also addresses this issue with findings from sensitivity trainings in coastal Kenya, which can influence and support clinical work with MSM who come to healthcare settings for sexually transmitted infection (STI) and HIV-related care and treatment. Additionally, innovative strategies for community mobilization engaging peer leaders in small-group safe spaces within stigmatized peri-urban townships are addressed through the article from South Africa.

While the role of sex among men is increasingly described in concentrated epidemic settings, studies from Southern Africa within *generalized* epidemics, where KPs are conventionally not thought to play a significant role (e.g., South Africa, Swaziland, Lesotho, Malawi, Namibia, Botswana and Zimbabwe), have also shown MSM to have high prevalence of HIV, syphilis and hepatitis B virus, with disease burdens equal to or greater than those of men in the GP. Still, because of the conception of KPs as insignificant in these generalized epidemic settings, any data collection or targeted response is limited [6].

Swaziland has been documented to have the highest HIV prevalence globally. The incidence of HIV appears to have peaked in 1998–1999 at 4.6% according to UNAIDS estimates, while in 2009 it was estimated to be 2.7%. Recent data from the Swaziland HIV Incidence Measurement Survey (SHIMS) estimated HIV incidence at 2.4% in the total population: 3.1% among women and 1.7% among men [1,7,8]. The 2009 Swaziland Modes of Transmission (MOT) study characterized major drivers of incident HIV infections to be multiple concurrent partnerships before and during marriage as well as low levels of male circumcision [9]. While these drivers were validated through the SHIMS, it is critical to note that like many MOT studies in generalized settings in Africa, there was no known prevalence among FSWs or MSM, so the MOT analysis assumed a low frequency of both practices and therefore assigned them as minor drivers of the epidemic.

In this issue, Baral and colleagues conducted the first cross-sectional study to estimate HIV prevalence and its risk factors among MSM in Swaziland [10]. The HIV prevalence in the RDS sample of 324 MSM was 17.6%, and the odds of HIV prevalence increased by 20% for each year of age. The vast majority (70%) of the sample reported being unaware of their HIV status, and consistent condom use with lubricants with male partners was reported by 12.6% of respondents. The authors note that within their MSM sample, HIV prevalence is consistent with that of an age-matched sample from the GP until age 24–26 years, when the prevalence of HIV among MSM rises *higher* than that of other men in the GP – with HIV prevalence of 43.1% among MSM older than 27 years [10].

These data as well as other recent data showing alarming rates (70.3%) among FSWs have called researchers to rethink the prevention, care and treatment response in Swaziland [11]. Similarly, in Malawi, a high-HIV-burden country in East Africa, Wirtz *et al*. conducted the most comprehensive MSM study to date in Malawi, where the HIV response has largely focussed exclusively on heterosexual and vertical transmission of HIV and where an estimated 8% of GP men have HIV [12,13]. A sample of 338 Malawi MSM had prevalence of HIV and active syphilis of 12.5 and 4.4%, respectively, after adjusting for RDS approaches. Ninety percent of HIV infections were previously undiagnosed, and about half reported consistent condom use with casual male partners. Among MSM 26 years and older, prevalence of HIV was 28.1%.

West and Central Africa, the most populous regions of Africa, have a mixture of HIV epidemics, and KPs are better understood to play an important role in the overall transmission dynamics in Nigeria, Senegal and Burkina Faso, where KPs consistently show elevated HIV prevalence in comparison to the GP, as reported by Papworth *et al*. in a meta-analysis of KPs from West and Central Africa [14]. In Cameroon, where the GP HIV prevalence for men is 2.9%, Park and colleagues sampled 511 MSM in Douala and Yaoundé through RDS and estimated the HIV prevalence to be 37% [15]. In Douala and Yaoundé, respectively, HIV prevalence was 25.5 and 44.4% [16]. Like in the other studies, the sample was young, with a median age of 24 years, and as age increased, HIV rates increased at staggering levels. HIV among MSM aged 24–29 years was 47%, and it was 49.4% for those over 30. About half of respondents did not use condoms consistently with casual partners (48.5%), and even more did not use them consistently with regular partners (64.1%). Similarly striking in Senegal, where GP prevalence among reproductive age men is 0.5%, the article included by Drame *et al*. reports baseline HIV prevalence among a sample of MSM at 36.0% (43/114) with cumulative HIV prevalence after 15 months at 47.2% (51/108) [17].

Due to the criminalized nature of male-to-male sex in all countries where studies from this issue took place, with the notable exception of South Africa (which still experiences high stigma), MSM are often afraid to visit healthcare services; and when they do go, they are reluctant to disclose their sexual histories to healthcare providers for fear of rejection, derision, or other negative reactions. Continued work is needed to develop violence screening, reporting and mitigation approaches within these settings [18]. The authors in this issue demonstrate that access to and coverage of quality HIV services for MSM are still marginal and not sufficient to reverse the epidemic's trend among MSM, and this is aggravated by several factors, primarily stigma, discrimination and limited domestic investment in programmes focussed on MSM [19–21]. Lessons learned from mature programmes targeting FSWs in the region show that with limited coverage, poor dosage and an inadequate combination of approaches, even proven interventions will be ineffective [22].

Research focussing on MSM sexual risk behaviour in SSA is scant, and even less is known about the stigmatization and discrimination of HIV-positive MSM [23]. Kennedy *et al*. conducted 40 in-depth interviews from 20 HIV-positive MSM, 16 interviews with key informants and three focus group discussions with MSM community members. Internalized and experienced stigma was high among the men living with HIV, who report that they conceal their HIV status from others. MSM living with HIV reported experiencing greater social isolation and lack of support for care-seeking and medication adherence. Perceived and experienced stigma from healthcare settings led to delayed care seeking and travel to more distant clinics to retain anonymity at home. The authors argue that mental health interventions, training for healthcare providers and better protection against discrimination are needed for Swazi MSM living with HIV, which corroborates previous findings from South Africa [24].

The association between HIV prevalence and the existence of community-based HIV interventions targeting MSM and other KPs has been well described [25]. Tailored community-based programmes that provide MSM with high-quality, sensitive services that are socially and economically acceptable and led by the beneficiaries themselves is a promising approach. The current article by Batist *et al*. expands on this association with the use of safe spaces to remove barriers to service access, including those aimed at training providers and mobilizing communities even within stigmatized peri-urban settings, which led to greater feelings of connection, social support and self-esteem among MSM community members and also led to these spaces becoming distribution points for condoms, lubricants and HIV education.

Also in this supplement, two articles look at a sensitivity training for healthcare workers providing services to MSM [21,26], highlighting the pre-existing attitudes that can manifest during clinical encounters with MSM. Healthcare workers in SSA generally do not receive specific training in working with MSM or other KPs, and they may not be aware of risk factors for HIV transmission or appropriate care and treatment needs. Healthcare worker training has been identified as a priority intervention to support a minimum package of essential services for MSM [27]. In van der Elst *et al*., the researchers implemented a novel approach to sensitivity training for healthcare workers providing services to MSM [21]. The training consisted of self-directed, publically available online modules followed by group discussions focussed on MSM sexual risks and healthcare needs. Knowledge and homophobia were assessed prior to training, immediately after training and three months post training. There was a statistically significant decline in homophobia sustained after three months post training, with greater reductions for males and those in clinical roles (doctors and nurses), who were also more likely to have higher homophobia scores pre-training. However, it remains to be seen whether these attitudes can be maintained over time without ongoing support [21].

In a subsequent article in this supplement, van der Elst *et al*. explored topics including the sexual identification of subcategories of MSM, sexual practices and risks for HIV and STI transmission, practices for sexual history taking and sexual health examinations for MSM [26]. Stigma was also a concern for healthcare workers, such as negative judgements from peers or community members for being associated with MSM, and was an ongoing challenge after the training. After completing the programme, healthcare workers expressed greater acknowledgement of MSM patients in their clinics, empowerment to address their needs, and a better understanding of the biological, behavioural and social influences that lead to HIV and STI risk for MSM.

The term “MSM” is meant to address all MSM, regardless of their gender or sexual identities. Some MSM self-identify as heterosexual rather than gay, homosexual, or bisexual, especially if they also have sex with women, are married, only take the penetrative role in anal sex, or have sex with men for money or convenience [28]. They may not consider their sexual encounters with other men in terms of gender identity or sexual orientation, or they may more aptly self-identify using local social terms which reference sexual identities, masculinity and femininity, and behaviours. One noteworthy finding within the articles presented in this issue from Malawi and Swaziland was the disconnection between gender identity and sexual orientation [10,12,20]. While nearly all the respondents of both surveys reported that they were either gay or bisexual and had anal sex with men as criteria for eligibility in the study, a sizeable number reported that they were not male. In Malawi, 17.0% reported they were female, and another 2.8% said they were transgender. In Swaziland, 15.7% reported being female, and 1.8% said they were both male and female. It is not clear whether participants actually considered themselves to be women or whether their sexual behaviour caused them to consider themselves not to be men. There is a need for further study to better understand how these terminologies translate into risk and sexual identity profiles while not singling out these individuals for further stigma.

As more data become available for MSM in SSA, including in Southern African generalized settings, MSM needs should be identified and addressed throughout the continuum of HIV prevention, care and treatment. We continue to need evidence-based interventions to identify, create and train healthcare providers as well as community champions, including lawyers, media owners, journalists and religious leaders sensitive to MSM programming, and establish community-driven programmes while expanding integration within the health system as appropriate.

The articles in this series have shown that throughout SSA, there is a significant and sustained epidemic among MSM, fear of discrimination from healthcare settings, and provider-based and self-stigma which impede prevention, care and treatment [29–31]. The findings highlight the need to focus on MSM as a critical KP in “mainstream” approaches as well as MSM-targeted models. Countries in Africa characterized as having generalized epidemics, where KPs are not considered relevant, must be re-conceptualized based on these findings. All “generalized” epidemics are in reality *mixed* epidemics with ongoing transmission among KPs, and this becomes increasingly clear as GP prevalence rates decline while MSM experience expanding epidemics.

While it is not clear what proportion of new HIV infections are linked to MSM directly and via second-order transmission among their partners, it is clear that without addressing this underserved, stigmatized population, HIV transmission will be impossible to abate. Therefore, the benefits of targeted structural, behavioural and biomedical services for MSM go beyond the individuals to benefit the larger public welfare and security of all countries within SSA as well as globally. After three decades of the fight against HIV, plans to end the HIV epidemic through goals such as the AIDS-free generation and the US President's Emergency Plan for AIDS Relief's (PEPFAR) Blueprint seem possible. Substantial progress has been made, and more will come through vigilance, courage, tolerance and commitment.

## References

[1] UNAIDS (2012). Report on the global AIDS epidemic.

[2] UNAIDS (2000). UNAIDS/WHO working group on global HIV/AIDS and STI surveillance. Guidelines for second generation HIV surveillance.

[3] Mishra S, Sgaier SK, Thompson LH, Moses S, Ramesh BM, Alary M (2012). HIV epidemic appraisals for assisting in the design of effective prevention programmes: shifting the paradigm back to basics. PLoS One.

[4] Wilson D, Halperin DT (2008). “Know your epidemic, know your response”: a useful approach, if we get it right. Lancet.

[5] Mayer KH, Bekker LG, Stall R, Grulich AE, Colfax G, Lama JR (2012). Comprehensive clinical care for men who have sex with men: an integrated approach. Lancet.

[6] Beyrer C (2012). LGBT Africa: a social justice movement emerges in the era of HIV. SAHARA J.

[7] Reed JB, Justman J, Bicego G, Donnell D, Bock N, Ginindza H (2012). Estimating national HIV incidence from directly observed seroconversions in the Swaziland HIV Incidence Measurement Survey (SHIMS) longitudinal cohort (FRLBX02).

[8] UNAIDS N (2010). Ministry of Health, Swaziland. Monitoring the declaration of the commitment on HIV and AIDS (UNGASS): Swaziland country report.

[9] HIV/AIDS (2009). UWBSNERCo. Swaziland: HIV prevention, response and modes of transmission analysis.

[10] Baral SD, Ketende S, Mnisi Z, Mabuza X, Grosso A, Sithole B (2013). A cross-sectional assessment of the burden of HIV and associated individual- and structural-level characteristics among men who have sex with men in Swaziland. J Int AIDS Soc.

[11] Baral L, Ketende S, Chen PA, Kennedy C, Mnisi Z, Adams D (2013). Reconceptualizing HIV prevention needs in a generalized epidemic: the case of female sex workers (FSW) in Swaziland.

[12] Wirtz AL, Jumbe V, Trapence G, Kamba D, Umar E, Ketende S (2013). HIV among men who have sex with men in Malawi: elucidating HIV prevalence and correlates of infection to inform HIV prevention. J Int AIDS Soc.

[13] Government of Malawi (2012). Global AIDS response progress report: Malawi country report for 2010 and 2011.

[14] Papworth E, Ceesay N, An L, Thiam-Niangoin M, Ky-Zerbo O, Holland C (2013). Epidemiology of HIV among female sex workers, their clients, men who have sex with men, and people who inject drugs in West and Central Africa. J Int AIDS Soc.

[15] Statistics CNIo (2012). 2011 Cameroon Demographic and Health Survey and Multiple Indicators Cluster Survey (DHS-MICS): HIV prevalence.

[16] Park JN, Papworth E, Kassegne S, Moukam L, Billong SC, Macauley I (2013). HIV prevalence and factors associated with HIV infection among men who have sex with men in Cameroon. J Int AIDS Soc.

[17] Dramé FM, Crawford EE, Diouf D, Beyrer C, Baral SD (2013). A pilot cohort study to assess the feasibility of HIV prevention science research among men who have sex with men in Dakar, Senegal. J Int AIDS Soc.

[18] Betron M (2009). Screening for violence against MSM and transgenders: report on a pilot project in Mexico and Thailand.

[19] Batist E, Brown B, Scheibe A, Baral SD, Bekker L (2013). Outcomes of a community-based HIV-prevention pilot programme for township men who have sex with men in Cape Town, South Africa. J Int AIDS Soc.

[20] Kennedy CE, Baral SD, Fielding-Miller R, Adams D, Dludlu P, Sithole B (2013). “They are human beings, they are Swazi”: intersecting stigmas and the positive health, dignity and prevention needs of HIV-positive men who have sex with men in Swaziland. J Int AIDS Soc.

[21] van der Elst EM, Smith AD, Gichuru E, Wahome E, Musyoki H, Muraguri N (2013). Men who have sex with men sensitivity training reduces homoprejudice
and increases knowledge among Kenyan healthcare providers in coastal Kenya. J Int AIDS Soc.

[22] Semini I, Batona G, Lafrance C, Kessou L, Gbedji E, Anani H (2013). Implementing for results: program analysis of the HIV/STI interventions for sex workers in Benin. AIDS Care.

[23] Nyblade L, Stangl A, Weiss E, Ashburn K (2009). Combating HIV stigma in health care settings: what works?. J Int AIDS Soc.

[24] Cloete A, Simbayi LC, Kalichman SC, Strebel A, Henda N (2008). Stigma and discrimination experiences of HIV-positive men who have sex with men in Cape Town, South Africa. AIDS Care.

[25] Gurung A, Narayanan P, Prabhakar P, Das A, Ranebennur V, Tucker S (2011). Large-scale STI services in Avahan improve utilization and treatment seeking behaviour amongst high-risk groups in India: an analysis of clinical records from six states. BMC Public Health.

[26] van der Elst EM, Gichuru E, Omar A, Kanungi J, Duby Z, Midoun M (2013). Experiences of Kenyan healthcare workers providing services to men who have sex with men: qualitative findings from a sensitivity training programme. J Int AIDS Soc.

[27] Beyrer C, Wirtz A, Walker D, Johns B, Sifakis F, Baral S (2011). The global HIV epidemics among men who have sex with men (MSM): Epidemiology, prevention, access to care and human rights.

[28] AMFAR (2009). Ensuring universal access to comprehensive HIV services for MSM in Asia and the Pacific: determining operations research priorities to improve HIV prevention, treatment, care, and support among men who have sex with men.

[29] Baral S, Adams D, Lebona J, Kaibe B, Letsie P, Tshehlo R (2011). A cross-sectional assessment of population demographics, HIV risks and human rights contexts among men who have sex with men in Lesotho. J Int AIDS Soc.

[30] Baral S, Burrell E, Scheibe A, Brown B, Beyrer C, Bekker LG (2011). HIV risk and associations of HIV infection among men who have sex with men in peri-urban Cape Town, South Africa. BMC Public Health.

[31] Fay H, Baral SD, Trapence G, Motimedi F, Umar E, Iipinge S (2011). Stigma, health care access, and HIV knowledge among men who have sex with men in Malawi, Namibia, and Botswana. AIDS Behav.
